# Targeting BCL11B-mediated JAK2/STAT3 activation: A novel therapeutic strategy for Parkinson’s disease

**DOI:** 10.1016/j.isci.2026.117833

**Published:** 2026-10-08

**Authors:** Wenwen Chen, Yejing Zhang, Xinrui Zhou, Jianan Yan, Yiming Song, Jiabing Shen, Xiaosu Gu

**Affiliations:** 1 Department of Neurology, Affiliated Hospital of Nantong University, Nantong 226001, P.R. China; 2 Research Center of Clinical Medicine, Affiliated Hospital of Nantong University, Nantong 226001, P.R. China; 3 Medical School of Nantong University, Nantong 226001, P.R. China; 4 Department of Neurology, Yancheng No. 1 People’s Hospital, Yancheng 224001, P.R. China

**Keywords:** Parkinson’s disease, BCL11B, RNA-Seq, JAK2/STAT3, neuroprotection

## Abstract

Parkinson’s disease (PD) is driven in part by neuroinflammation. We investigated whether the transcription factor BCL11B contributes to dopaminergic neurodegeneration in this context. Transcriptome sequencing of MPTP-induced PD mice revealed BCL11B upregulation, which was confirmed in MPP^+^-treated MN9D cells, MPTP-lesioned mice, and dopaminergic neurons exposed to inflammatory conditioned medium. BCL11B knockdown attenuated dopaminergic neuron loss, reduced apoptosis, improved motor deficits, and partially blocked JAK2/STAT3 pathway activation in both inflammatory and MPTP-induced PD models. Mechanistically, BCL11B mediated neurotoxicity through the JAK2/STAT3 pathway, with acetylation modifications potentially modulating its activity. These findings establish BCL11B as a key regulator of neuroinflammation and dopaminergic survival in PD, and suggest that targeting BCL11B may offer a therapeutic approach for this neurodegenerative disease.

## Highlights

• BCL11B is upregulated in Parkinson’s disease models
• BCL11B inhibition protects dopaminergic neurons from loss
• BCL11B promotes neurotoxicity via JAK2/STAT3 activation
• BCL11B is a potential therapeutic target for Parkinson’s disease

## Graphical abstract

**Figure:**
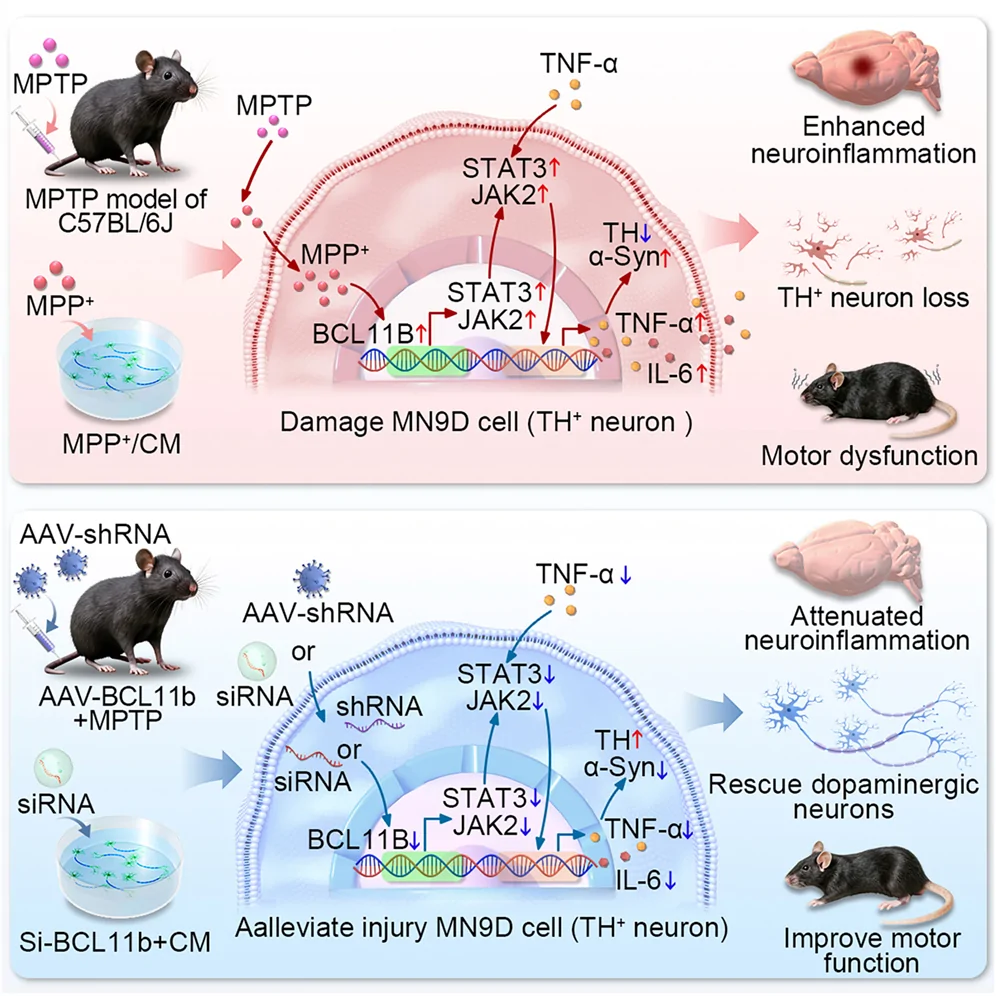

## Teaser

Neurology; Therapy; Molecular biology

## Introduction

PD is the second most common neurodegenerative disorder after Alzheimer’s disease (AD), significantly impacting patients’ quality of life.^1^ The hallmark pathological feature of PD is the degeneration of dopaminergic neurons in the substantia nigra pars compacta of the midbrain. This neuronal loss leads to reduced dopamine levels, resulting in motor symptoms—including bradykinesia, resting tremor, and muscle rigidity—as well as non-motor manifestations such as anxiety and depression.^2^ The pathogenesis of PD is multifactorial, with neuroinflammation recognized as a key driver of disease onset and progression.^3^ Under inflammatory conditions, overactivated microglia release abundant pro-inflammatory factors—including TNF-α, IL-1β, IL-2, IL-4, and IL-6—that impair the function of normal neurons and glial cells.^4^

B-cell lymphoma/leukemia 11B (BCL11B) is a transcription factor that encodes a C2H2-type zinc finger protein.^5^ In adult mice, BCL11B is expressed in multiple brain regions, including the cerebral cortex, limbic system, olfactory bulb, basal ganglia, and hippocampus, and serves as a key regulator of neuronal development.^6^ A genome-wide study involving 41 patients with sporadic late-onset AD demonstrated that BCL11B negatively regulates the BDNF signaling pathway, resulting in reduced levels of brain-derived neurotrophic factor (BDNF) and accelerated disease progression.^7^ Additionally, studies have reported decreased BCL11B expression in the cerebrospinal fluid and brain tissues of patients with Huntington’s disease. Overexpression of BCL11B in the Q111 cell model significantly attenuated mutant huntingtin-induced neuronal damage and exerted neuroprotective effects.^8^

BCL11B also plays a critical role in the immune system, where it regulates T cell differentiation and proliferation, thereby modulating immune responses. In models of allergic asthma and helminth infection, deletion of BCL11B in T cells markedly attenuates the Th2 cell-mediated immune response.^9^ Meanwhile, studies have shown that the traditional Chinese herbal formula Bimin Kang can alleviate minimal persistent inflammation in allergic rhinitis by downregulating BCL11B expression.^10^ In the context of neurological disorders, BCL11B expression was found to be significantly elevated in the cerebrospinal fluid and brain tissue of HIV-positive patients with latent infection compared to healthy controls, accompanied by increased levels of inflammatory factors such as IL-6 and TNF-α. Immunofluorescence staining of postmortem brain samples from these patients revealed BCL11B expression in neurons, microglia, and astrocytes, suggesting its potential involvement in neuroinflammatory processes.^11^ In recent years, sustained neuroinflammation-driven degeneration of dopaminergic neurons has been recognized as a key mechanism in the pathology of PD.^12^ We therefore hypothesize that BCL11B may play an important role in this process and influence disease progression.

Pro-inflammatory factors such as TNF-α can bind to receptors on the surface of neuronal cells and trigger JAK2 activation.^13^ Activated JAK2 phosphorylates tyrosine residues on the receptors, leading to the recruitment of STAT3. Subsequently, phosphorylated STAT3 forms dimers, translocates into the nucleus, binds to gene promoter regions, and regulates the expression of inflammation-related genes. This cascade exacerbates neuroinflammation and promotes neuronal death.^14^ Aberrant activation of the JAK2/STAT3 pathway has been demonstrated to contribute to neurotoxicity in neurodegenerative diseases, including AD^15^ and PD.^16^ Considering the property of BCL11B, we speculated that the JAK2/STAT3 signaling pathway may act as a potential downstream mechanism in PD.

Histone deacetylases (HDACs) represent a pivotal family of epigenetic modifying enzymes that regulate acetylation levels of both histones and non-histone proteins. They are extensively involved in multiple pathological processes, including neuroinflammation and neuronal apoptosis, and exert essential regulatory effects on the pathogenesis and progression of neurodegenerative diseases such as PD.^17^

Nevertheless, the exact biological function and underlying regulatory mechanism of BCL11B in PD remain poorly clarified. Here, we aimed to determine whether BCL11B contributes to dopaminergic neuronal loss and neuroinflammation during PD pathogenesis. To this end, we combined MPTP-induced mouse PD models and MPP^+^-treated neuronal cell lines to detect the expression pattern of BCL11B, determine its biological function via loss-of-function experiments, and further validate the regulatory relationship between BCL11B and the JAK2/STAT3 signaling pathway. This work aims to provide novel experimental evidence to understand the molecular mechanisms of neuroinflammation in PD and identify a promising inflammatory therapeutic target.

## Results

### RNA-Seq implicates BCL11B in PD pathological progression

To investigate the molecular mechanisms underlying PD, we established an acute PD model by administering intraperitoneal injections of MPTP (20 mg/kg, 4 doses at 2-h intervals) to 8-week-old male C57BL/6J mice, with saline-injected mice serving as controls. Seven days post-treatment, bilateral substantia nigra tissues were collected for transcriptome sequencing (Figure 1A). Comparative analysis identified 225 upregulated and 148 downregulated genes in MPTP-treated mice compared to controls (Figure 1B). Gene Ontology (GO) analysis indicated significant differences in terms related to “dopaminergic binding” and “dopaminergic synaptic transmission” (Figure 1C). KEGG pathway analysis revealed enrichment in “tyrosine metabolism” and “neuroactive ligand-receptor interaction” pathways (Figure 1D), consistent with the characteristic loss of dopaminergic neurons in PD and confirming successful model establishment.

**Figure 1:**
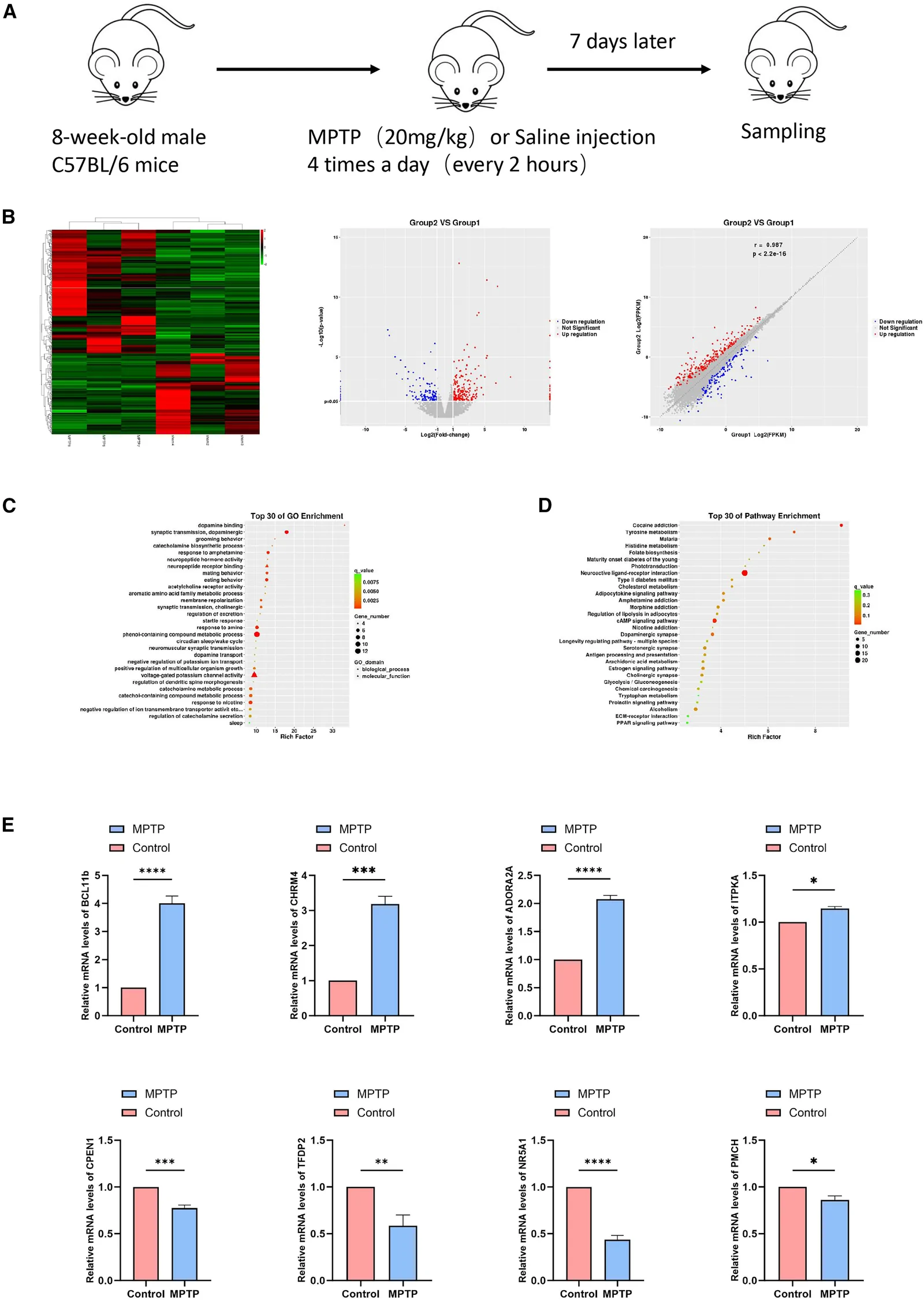
RNA sequencing identifies BCL11B involvement in PD pathogenesis (A) Schematic of model establishment. (B) Heatmap, volcano plot, and scatterplot of differentially expressed genes. (C) GO analysis of differentially expressed genes. (D) KEGG analysis of differentially expressed genes. (E) qRT-PCR analysis of selected differentially expressed genes in the midbrain of PD mice. (Data are represented as mean ± SEM. Statistical significance was determined by one-way ANOVA followed by Sidak’s post hoc test. ∗*p* < 0.05, ∗∗*p* < 0.01, ∗∗∗*p* < 0.001, and ∗∗∗∗*p* < 0.0001.).

Among the differentially expressed genes, we focused on BCL11B, a transcription factor implicated in neuronal development and immune regulation. qRT-PCR validation confirmed a significant upregulation of BCL11B at the mRNA level (Figure 1E). These results suggest that BCL11B may play a role in the pathogenesis of PD.

### Expression dynamics and acetylation modulation of BCL11B in *in vivo* and *in vitro* PD models

To investigate the role of BCL11B in PD pathogenesis, we utilized the MN9D dopaminergic cell line to establish an *in vitro* PD model. Double immunofluorescence staining confirmed the expression and co-localization of BCL11B with tyrosine hydroxylase (TH) in MN9D cells (Figure 2A), accompanied by consistent changes in fluorescence intensity (Figure 2B). A conventional MPP^+^-induced PD model was established, with 100 μM MPP^+^ inducing approximately 50% cell death after 24 h (Figure 2C). Western blot analysis revealed a time-dependent upregulation of BCL11B protein in MPP^+^-treated MN9D cells (Figures 2D and 2E). Similarly, in MPTP-induced acute PD mouse models, BCL11B expression progressively increased over 3, 7, and 14 days (Figures 2F and 2G). Immunofluorescence co-staining further demonstrated BCL11B expression in neurons, dopaminergic neurons, microglia, and astrocytes (Figure 2H).

**Figure 2:**
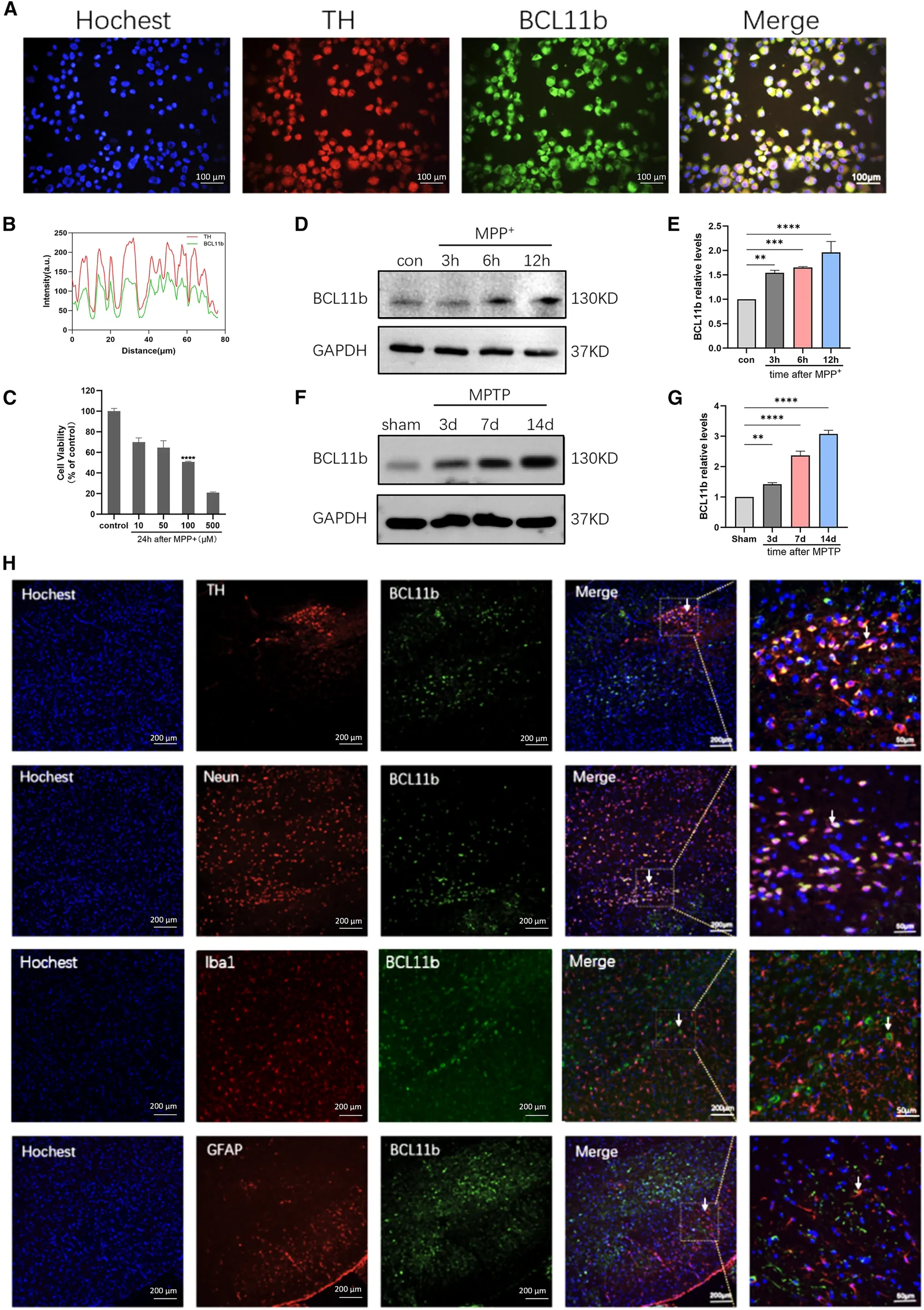
Expression patterns of BCL11B in cellular and animal PD models (A) Co-localization of TH and BCL11B in MN9D cells. Scale bars, 100 μm. (B) Quantification of fluorescence intensity. (C) CCK-8 assay. (D and E) BCL11B protein levels in the MPP^+^ model and quantification. Representative images are shown from 3 number of independent repeats. (F and G) BCL11B protein levels in the MPTP model and quantification. Representative images are shown from 3 number of independent repeats. (H) Double immunofluorescence staining of BCL11B together with NeuN, TH, IBA1, or GFAP in brain sections from MPTP-treated mice. Scale bars, 200 μm (low-magnification images); 50 μm (high-magnification insets). (Data are represented as mean ± SEM. Statistical significance was determined by one-way ANOVA followed by Sidak’s post hoc test. ∗*p* < 0.05, ∗∗*p* < 0.01, ∗∗∗*p* < 0.001, and ∗∗∗∗*p* < 0.0001.).

Previous work by our group has shown that HDAC3 interacts with multiple transcription factors and contributes to neurotoxicity, prompting further investigation into HDAC family involvement. Given that HDAC members influence neuronal survival via histone deacetylation, we assessed global acetylation levels in both MPP^+^-treated MN9D cells and MPTP-induced mouse models. We observed a gradual increase in total protein acetylation during disease progression (Figures 3A–3D). Using IPA software, we predicted potential interactions between BCL11B and HDAC family members, identifying HDAC1, HDAC2, HDAC3, and HDAC6 as likely interactors (Figure 3E). Co-immunoprecipitation assays further confirmed that BCL11B undergoes acetylation modification in the MPP^+^-induced cellular model (Figure 3F). Moreover, BCL11B was found to interact with HDAC1 and HDAC2, suggesting that its acetylation status may be regulated by these deacetylases.

**Figure 3:**
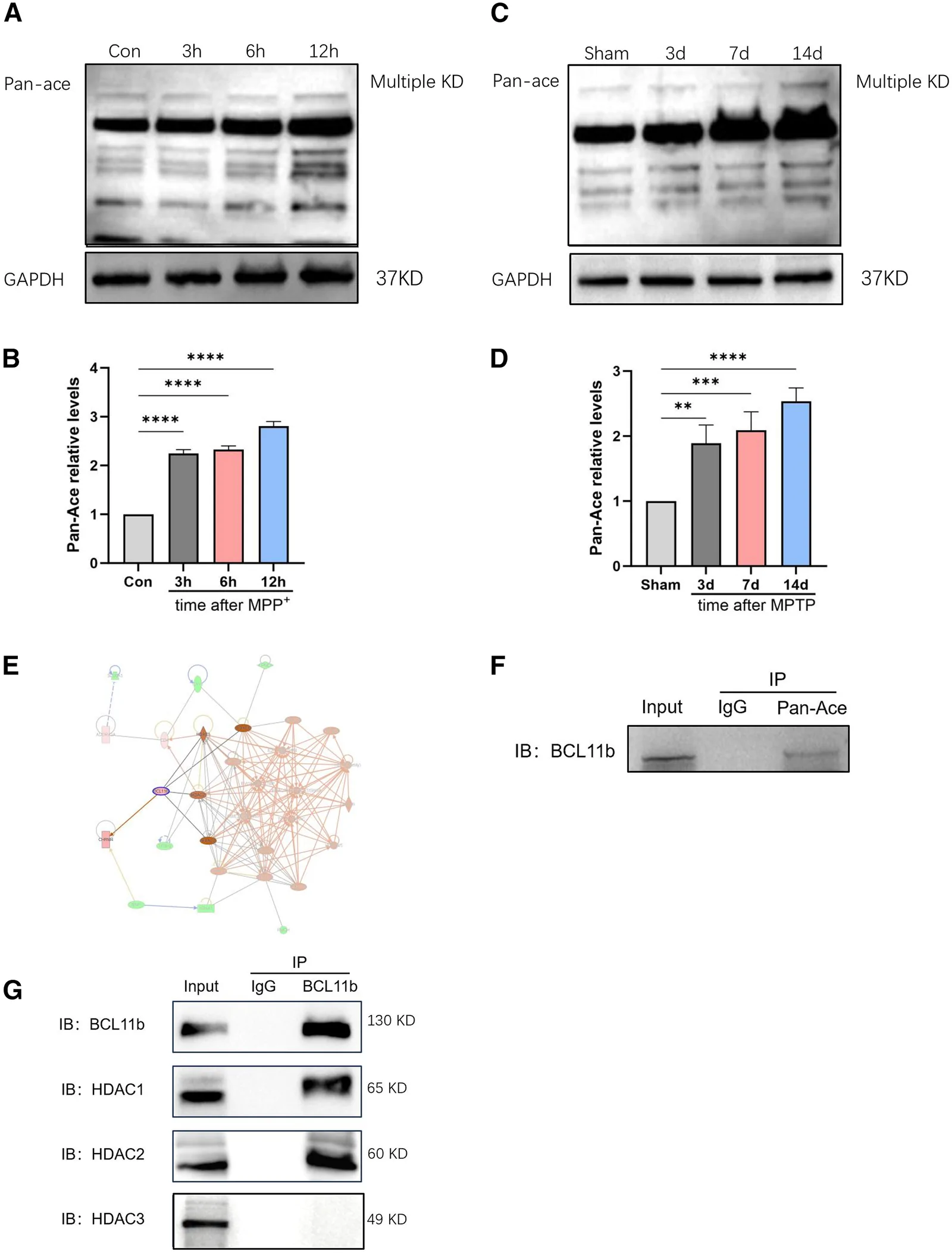
Acetylation dynamics of BCL11B in PD models (A and B) Pan-acetylation expression levels and their statistics in the MPP+-induced MN9D cell model. Representative images are shown from 3 number of independent repeats. (C and D) Pan - acetylation levels and their statistics in the MPTP-induced mouse model. Representative images are shown from 3 number of independent repeats. (E) Prediction by IPA software of the interactions between BCL11b and the HDAC family (based on RNA-seq data, not experimentally validated); (F) Validation by Co/IP of the acetylation expression of BCL11b in MPP+-induced MN9D cells. Representative images are shown from 3 number of independent repeats. (G) Validation by Co/IP of the interactions between BCL11b and HDAC1, HDAC2, and HDAC3. Representative images are shown from 3 number of independent repeats. (Data are represented as mean ± SEM. Statistical significance was determined by one-way ANOVA followed by Sidak’s post hoc test. ∗*p* < 0.05, ∗∗*p* < 0.01, ∗∗∗*p* < 0.001, and ∗∗∗∗*p* < 0.0001).

In summary, BCL11B expression is significantly elevated in both cellular and murine PD models, and its acetylation modification may contribute to PD pathology.

### Expression profile of BCL11B in MN9D cells under inflammatory culture conditions

Neuroinflammation is a critical contributor to PD pathogenesis. Aberrantly aggregated α-synuclein can activate microglia, leading to the release of pro-inflammatory cytokines and subsequent neuroinflammatory damage to neighboring neurons. Reports of elevated BCL11B levels in HIV-positive patients with latent infection—accompanied by increased IL-6 and TNF-α—suggest a potential link between BCL11B and neuroinflammatory processes.

To simulate a neuroinflammatory milieu, we stimulated BV2 microglial cells with 1 μg/mL LPS for 6 h, after which the medium was replaced with fresh culture medium for an additional 24 h (Figure 4A). Meanwhile, the residual BV2 cells were collected to quantify the transcriptional expression of inflammatory mediators iNOS, COX-2, TNF-α, and IL-6 via qRT-PCR. Quantitative real-time PCR analysis revealed that the expression levels of these pro-inflammatory factors were significantly elevated in LPS-exposed BV2 cells relative to untreated control cells (Figure 4B).

**Figure 4:**
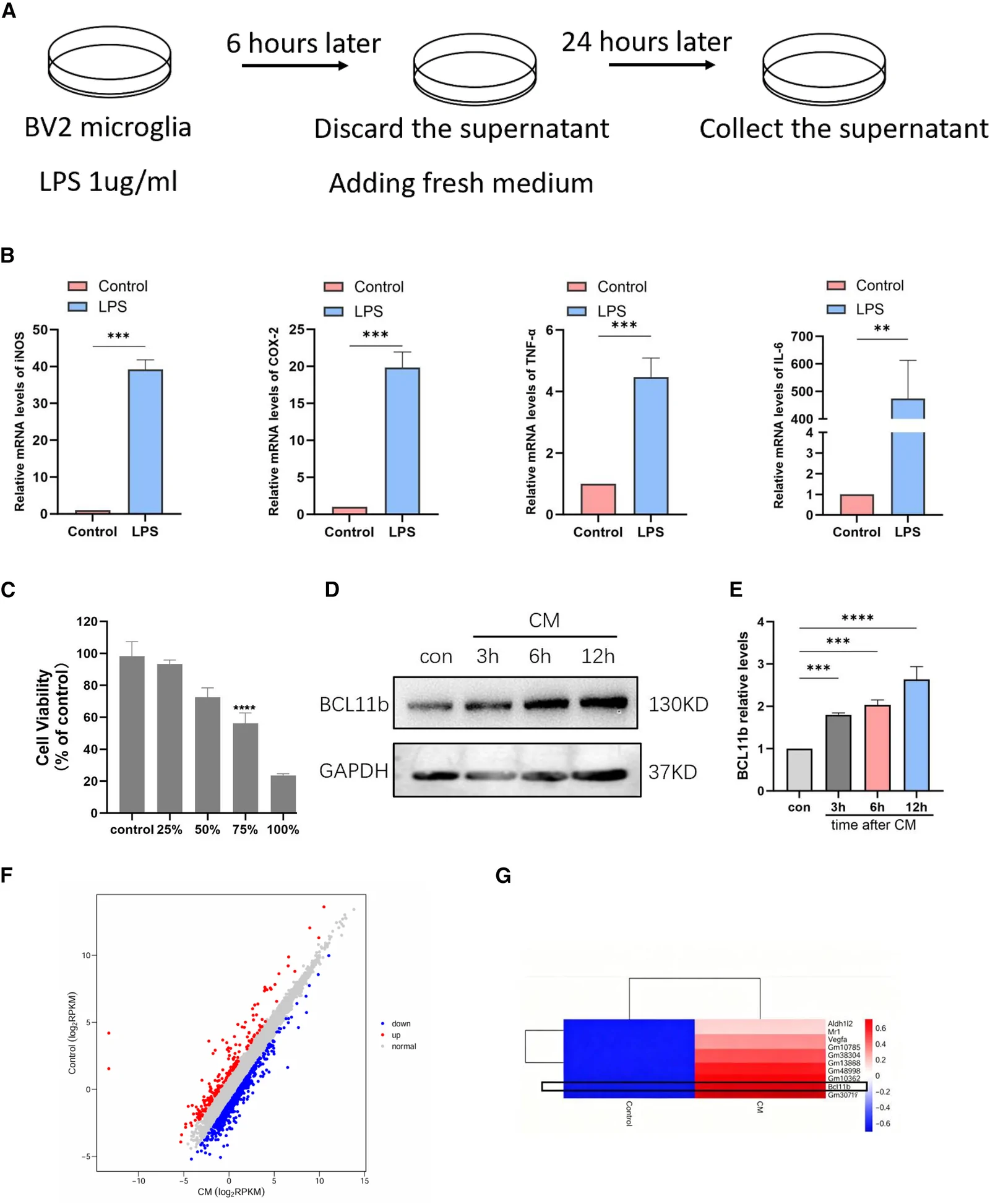
BCL11B expression in MN9D cells under inflammatory stimulation (A) Schematic of inflammatory conditioned-medium preparation. (B) qRT-PCR analysis of iNOS, COX-2, TNF-α, and IL-6 mRNA levels in BV2 cells. Each biological sample was measured in three technical replicates. (C) CCK-8 cell-viability assay. (D and E) BCL11B protein expression in the inflammatory conditioned-medium model and quantification. Representative images are shown from 3 number of independent repeats. (Data are represented as mean ± SEM. Statistical significance was determined by one-way ANOVA followed by Sidak’s post hoc test. ∗*p* < 0.05, ∗∗*p* < 0.01, ∗∗∗*p* < 0.001, and ∗∗∗∗*p* < 0.0001.).

To model inflammation-induced neuronal death, MN9D cells were treated with varying proportions of inflammatory conditioned medium (0–100%) for 24 h. CCK-8 assays indicated that 75% inflammatory medium reduced cell viability to approximately 50%, establishing this as the optimal concentration for subsequent experiments (Figure 4C). Western blot analysis revealed a marked upregulation of BCL11B protein in MN9D cells under these conditions, with peak expression observed at 12 h (Figures 4D and 4E). RNA sequencing of MN9D cells exposed to 75% inflammatory medium for 12 h identified multiple differentially expressed genes (Figure 4F), among which BCL11B was significantly upregulated (Figure 4G), consistent with prior results.

In summary, BCL11B expression is substantially induced in an inflammation-driven cellular model of PD, suggesting its involvement in inflammation-mediated neuronal death. Additional post-translational modifications such as phosphorylation or ubiquitination may also contribute to its regulation, warranting further investigation.

### BCL11B knockdown confers neuroprotection *in vitro* and *in vivo*

To further investigate the mechanism by which BCL11B influences neuroinflammation-induced neuronal death. MN9D cells were pretreated with BCL11B-specific or control siRNA for 60 h, followed by stimulation with 75% inflammatory conditioned medium for 12 h (Figure 5A). Among several siRNA constructs tested, the siRNA targeting site 333 (siRNA No.2) exhibited the highest knockdown efficiency, as confirmed at both the mRNA and protein levels (Figures 5B–5D).

**Figure 5:**
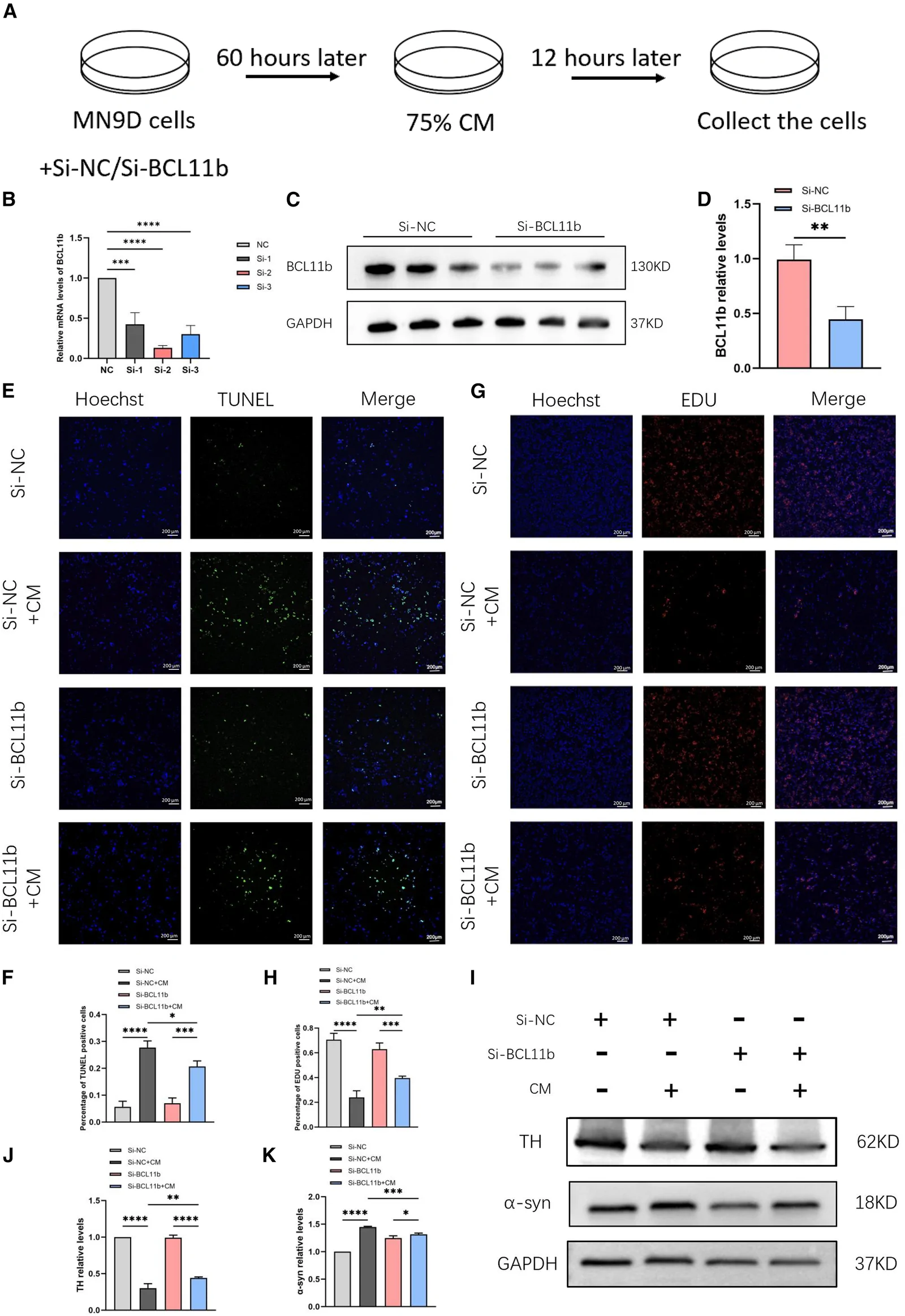
BCL11B inhibition protects MN9D cells from inflammatory injury (A) Schematic of model establishment. (B) qRT-PCR analysis of siRNA knockdown efficiency, with three technical wells per biological sample. (C and D) Western blot analysis of siRNA knockdown efficiency. Representative images are shown from 3 number of independent repeats. (E and F) TUNEL analysis of apoptosis. Scale bars, 200 μm. (G and H) EdU analysis of cell proliferation. Scale bars, 200 μm. (I–K) Western blot analysis of TH and α-synuclein. Representative images are shown from 3 number of independent repeats. (Data are represented as mean ± SEM. Statistical significance was determined by one-way ANOVA followed by Sidak’s post hoc test. ∗*p* < 0.05, ∗∗*p* < 0.01, ∗∗∗*p* < 0.001, and ∗∗∗∗*p* < 0.0001.).

TUNEL staining showed that inflammatory stimulation markedly increased apoptosis, which BCL11B knockdown significantly mitigated (Figures 5E and 5F). Similarly, EdU incorporation assays revealed that inflammatory medium suppressed MN9D cell proliferation, and this effect was partially reversed upon BCL11B inhibition (Figures 5G and 5H). Western blot analysis indicated that inflammatory stimulation downregulated TH and upregulated α-syn expression, whereas BCL11B knockdown restored TH levels and reduced α-syn accumulation (Figures 5I–5K).

To further validate the role of BCL11B in PD, we performed BCL11B knockdown in MPTP-induced C57BL/6J mice. As outlined in Figure 6A, 8-week-old mice received stereotaxic injections of either AAV-control or AAV-BCL11B-shRNA. Two weeks later, a PD model was established via intraperitoneal MPTP administration, followed by behavioral assessments of motor function.

**Figure 6:**
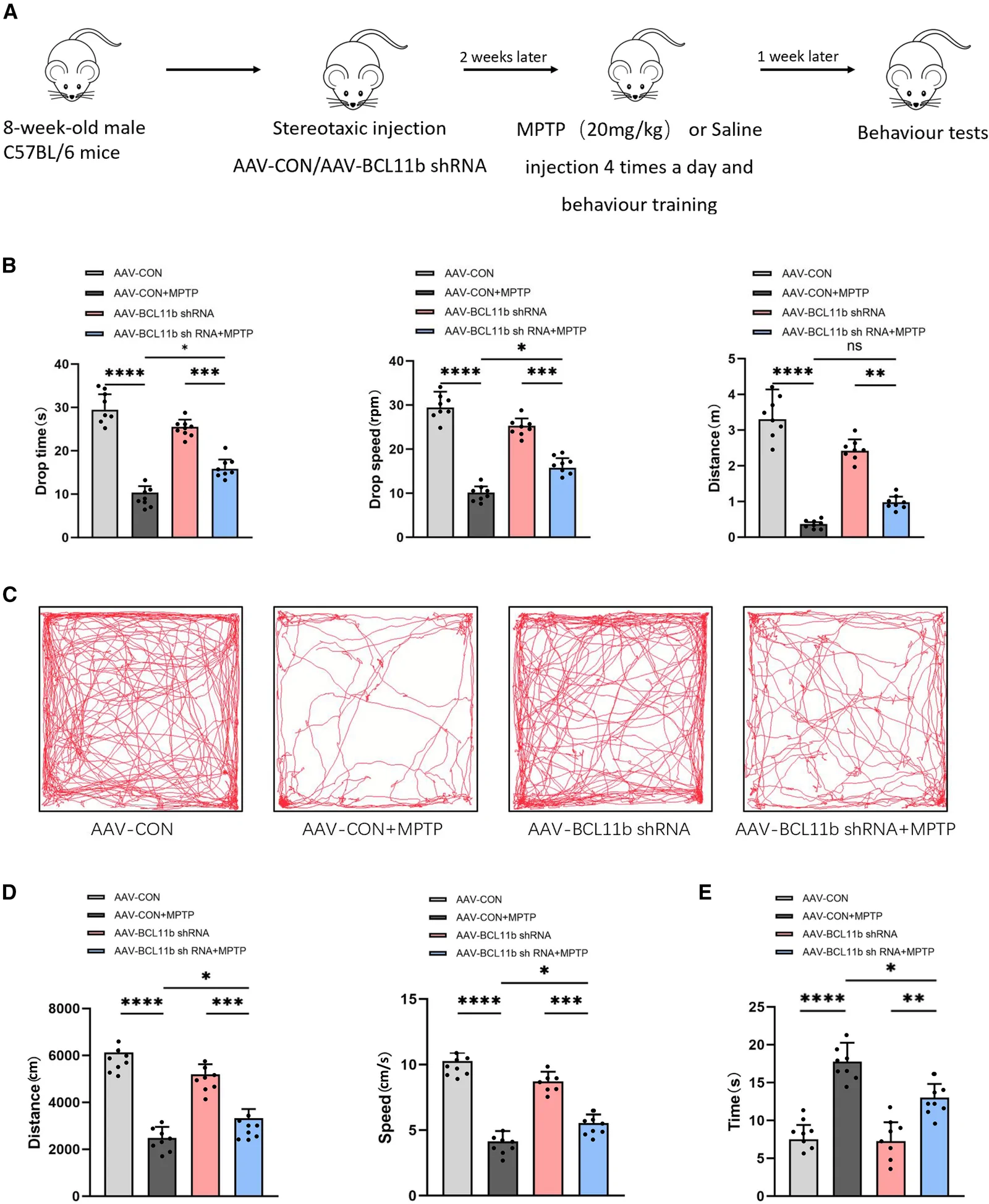
BCL11B knockdown ameliorates motor deficits in MPTP-induced mice (A) Flowchart of model establishment. (B) Rotarod test to detect the motor function of PD mice (number of mice *n* = 8). (C) Representative diagram of the movement trajectories of mice in the open-field test (number of mice *n* = 8). (D) Statistical chart of data analysis in the open-field test. (E) Pole-climbing test to detect the motor coordination ability of mice (number of mice *n* = 8). (Data are represented as mean ± SEM. Statistical significance was determined by one-way ANOVA followed by Sidak’s post hoc test. ∗*p* < 0.05, ∗∗*p* < 0.01, ∗∗∗*p* < 0.001, and ∗∗∗∗*p* < 0.0001).

In the rotarod test, which evaluates motor coordination and balance, MPTP-treated mice exhibited significantly shorter fall latencies and reduced speed compared to controls. BCL11B knockdown improved fall latency and speed, though no significant change was observed in total distance traveled (Figure 6B). The open field test revealed decreased total movement distance and speed in MPTP-injured mice, which were partially restored by BCL11B inhibition (Figure 6C). Further analysis confirmed that BCL11B knockdown significantly ameliorated MPTP-induced motor deficits (Figure 6D). In the pole test, MPTP-administered mice displayed prolonged descent time and impaired motor coordination, both of which were mitigated by BCL11B knockdown.

Upon completion of behavioral tests, midbrain tissues were collected for analysis. Western blot confirmed efficient BCL11B knockdown in the midbrain (Figures 7A and 7B). Immunofluorescence staining of the substantia nigra showed that MPTP reduced dopaminergic neuron signals and increased BCL11B expression, whereas BCL11B knockdown attenuated neuronal loss (Figures 7C–7E). Western blot analysis further demonstrated that MPTP decreased TH expression and increased α-syn levels, effects that were partially reversed by BCL11B knockdown (Figures 7F–7H).

**Figure 7:**
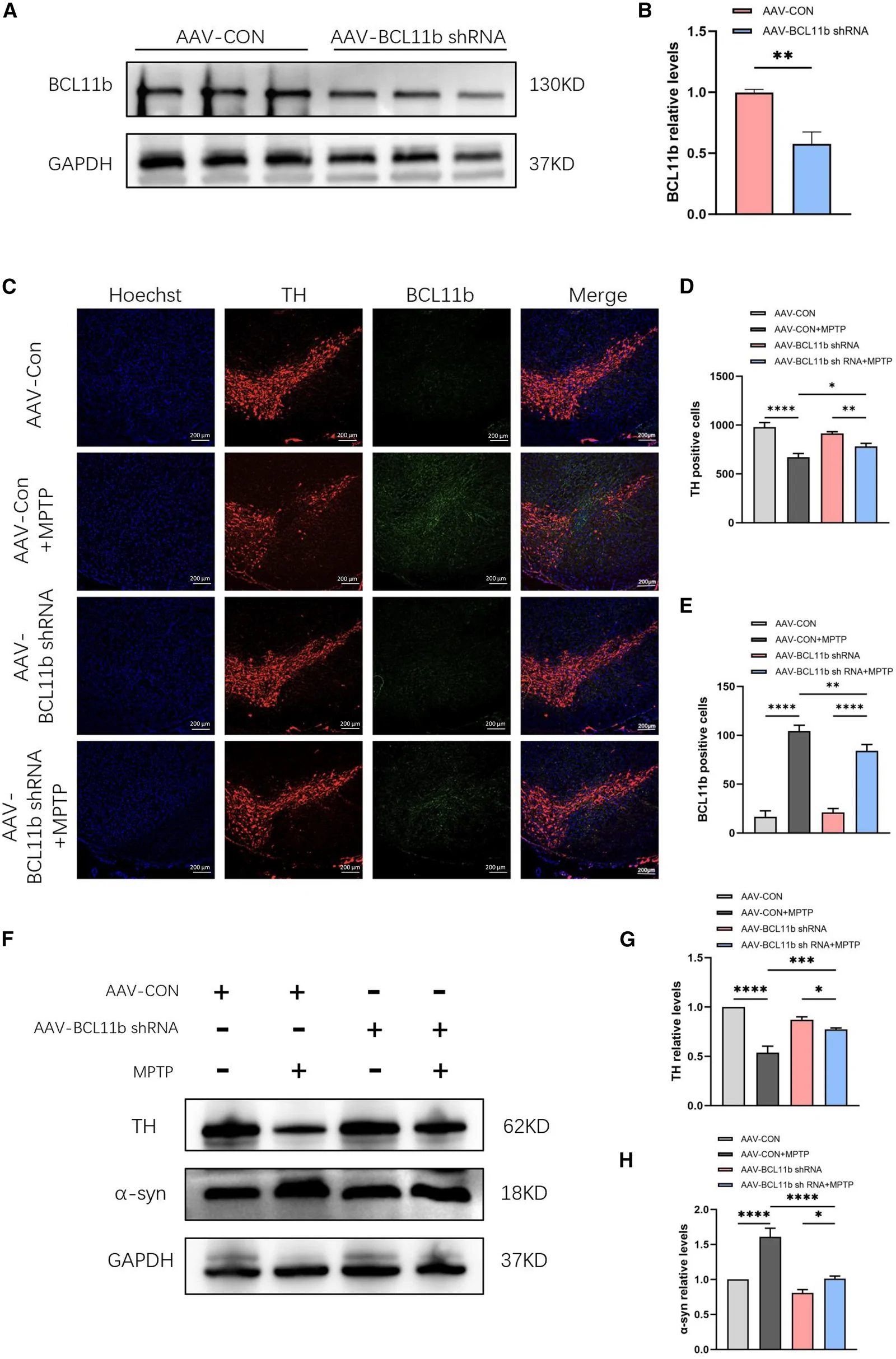
Neuroprotection by BCL11B suppression in MPTP mouse model (A and B) Detection of the expression level of BCL11b by western blot. Representative images are shown from 3 number of independent repeats. (C–E) Double immunofluorescence staining to detect the fluorescence intensities of TH and BCL11b and statistical analysis (number of independent repeats *n* = 3). Scale bars, 200 μm. (F–H) Detection of the expression levels of TH and α-syn in the MPTP-induced mouse model by western blotting. Representative images are shown from 3 number of independent repeats. (Data are represented as mean ± SEM. Statistical significance was determined by one-way ANOVA followed by Sidak’s post hoc test. ∗*p* < 0.05, ∗∗*p* < 0.01, ∗∗∗*p* < 0.001, and ∗∗∗∗*p* < 0.0001).

In summary, BCL11B inhibition exerts prominent neuroprotective effects both *in vitro* and *in vivo*, it ameliorates neuronal apoptosis, promotes cell proliferation and preserves dopaminergic integrity in inflammation-induced PD cell models, and alleviates motor deficits as well as mitigates dopaminergic neuron loss in the MPTP-induced mouse model of PD.

### The JAK2/STAT3 pathway contributes to BCL11B-mediated pathology in PD

To investigate the neurotoxic mechanisms of BCL11B, we utilized the IPA network to predict its downstream effectors. The analysis revealed that BCL11B may activate multiple pro-inflammatory factors, including TNF, IL-2, and IL-17 (Figure 8A). Subsequently, we validated the above prediction in MN9D cells stimulated with inflammatory conditioned medium. The results showed that inflammatory conditioned medium upregulated the level of TNF-α, whereas BCL11B inhibition reversed this elevation. In contrast, IL-2 and IL-17 exhibited no significant alterations under our experimental conditions (Figure 8B). Literature evidence suggests that TNF-α can directly activate JAK2, amplifying inflammation through the JAK2/STAT3 pathway and contributing to dopaminergic neuron degeneration. We therefore assessed whether this pathway is involved in BCL11B-mediated pathology in our experimental models.

**Figure 8:**
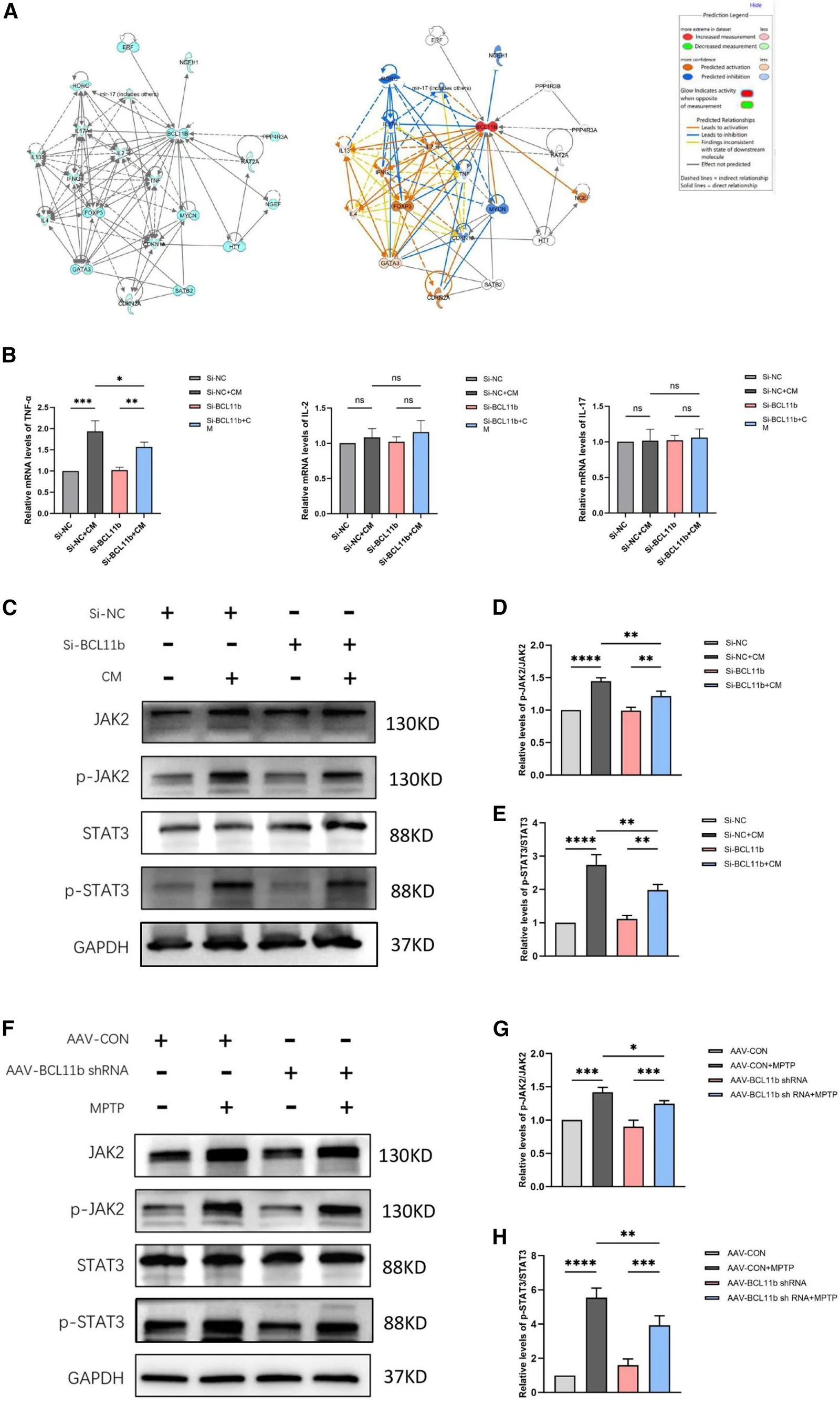
BCL11B activates the JAK2/STAT3 pathway in PD models (A) IPA prediction of factors downstream of BCL11B. (B) qRT-PCR analysis of pro-inflammatory factors in the inflammatory conditioned-medium model, with three technical wells per biological sample. (C–E) JAK2/STAT3 pathway proteins in the inflammatory conditioned-medium model and quantification. Representative images are shown from 3 number of independent repeats. (F–H) JAK2/STAT3 pathway proteins in the MPTP mouse model and quantification. Representative images are shown from 3 number of independent repeats. (Data are represented as mean ± SEM. Statistical significance was determined by one-way ANOVA followed by Sidak’s post hoc test. ∗*p* < 0.05, ∗∗*p* < 0.01, ∗∗∗*p* < 0.001, and ∗∗∗∗*p* < 0.0001.).

In the inflammation-induced cellular model, western blot analysis showed that stimulation with inflammatory conditioned medium significantly elevated levels of phosphorylated JAK2 (*p*-JAK2) and STAT3 (p-STAT3), whereas BCL11B knockdown partially suppressed this activation (Figures 8C–8E). Similarly, in the MPTP-induced mouse model, MPTP treatment enhanced JAK2/STAT3 phosphorylation, and BCL11B inhibition partially reversed this effect (Figures 8F–8H).

These findings indicate that BCL11B acts upstream of the JAK2/STAT3 signaling cascade and participates in neuroinflammation-driven PD pathology by promoting its activation.

## Discussion

With the progression of global aging, PD increasingly impairs quality of life and functional autonomy in affected individuals. The intricate pathogenesis of PD remains a substantial challenge in therapeutic development.^18^ Neuroinflammation has been widely recognized as a pivotal driver in the progression of PD.^19^ In this study, we demonstrated that suppression of BCL11B under inflammatory conditions partially rescues dopaminergic neurons from apoptosis. Importantly, we identified that BCL11B mediates neurotoxicity primarily through activation of the JAK2/STAT3 signaling pathway.

In pursuit of novel therapeutic targets for PD, we conducted transcriptomic sequencing in an acute MPTP-induced mouse model. Among differentially expressed genes, BCL11B—a transcription factor expressed in diverse tissues including the immune and central nervous systems—was selected for further analysis. Previous embryological studies indicated that BCL11B modulates neuronal differentiation and survival through regulation of genes such as DSP, CALB1, HTR2C, and TACR3.^20^ In the present work, immunostaining in MPP^+^-treated MN9D cells revealed co-localization of BCL11B with tyrosine hydroxylase (TH) and its marked upregulation following neurotoxic insult. A consistent expression pattern was observed in MPTP-induced mice, with BCL11B detected in dopaminergic neurons and microglia.

Epigenetic dysregulation,^21^ including alterations in histone acetylation,^22^ has been increasingly implicated in PD pathogenesis. Our group previously reported that HDAC3 modulates transcription factors such as SIRT6 and HIF-1α, contributing to dopaminergic degeneration. Here, we observed a global increase in protein acetylation in both cellular and murine PD models, suggesting widespread epigenetic alterations during disease progression. Through IPA-based prediction and co-immunoprecipitation assays, we further identified an association between BCL11B and histone deacetylases, accompanied by altered acetylation of BCL11B itself. These findings imply that post-translational modification of BCL11B may influence its functional properties and contribute to neurodegeneration in PD.

BCL11B is expressed in multiple brain regions and has been linked to several neurological disorders.^23^ For instance, reduced BCL11B levels are reported in Huntington’s disease, where its overexpression mitigates mutant huntingtin toxicity.^7^^,^^24^ Conversely, in latent HIV infection, elevated BCL11B correlates with neuroinflammation.^25^ Given the central role of neuroinflammation in PD—where sustained microglial activation and cytokine release exacerbate neurodegeneration^26^—we stimulated MN9D cells with conditioned medium from LPS-activated BV2 microglia. This inflammatory challenge significantly upregulated BCL11B expression.

To assess the functional relevance of BCL11B in PD, we performed knockdown experiments *in vitro* and *in vivo*. In MN9D cells, BCL11B silencing reduced apoptosis, enhanced proliferation, and restored TH expression while decreasing α-synuclein accumulation. In MPTP-treated mice, BCL11B knockdown improved motor performance in behavioral assays and attenuated dopaminergic neuron loss, supporting its neuroprotective potential.

To elucidate the underlying mechanism, we employed IPA analysis and identified multiple pro-inflammatory factors downstream of BCL11B, including IFNG, TNF, IL-2, and IL-17, which are known activators of the JAK2/STAT3 pathway. This pathway plays a well-established role in inflammation and neurodegeneration, and its overactivation is implicated in PD.^27^ We found that both inflammatory conditioned medium and MPTP administration enhanced phosphorylation of JAK2 and STAT3, effects that were attenuated by BCL11B inhibition. These results indicate that BCL11B functions upstream of the JAK2/STAT3 cascade to promote neurotoxicity.

In conclusion, our findings establish BCL11B as a novel regulator of neuroinflammation and dopaminergic degeneration in PD, acting through epigenetic mechanisms and JAK2/STAT3 signaling. Targeting BCL11B may offer a promising therapeutic strategy, though further translational studies are required to evaluate its clinical applicability.

### Limitations of the study

Despite the credible findings revealed in the present research, several inherent limitations should be acknowledged. First, all animal experiments in this study were performed using an acute MPTP-induced PD mouse model. We did not employ a chronic MPTP administration regimen or α-synuclein pre-formed fibril (PFF) models; therefore, the regulatory role of BCL11B in long-term chronic neurodegeneration and pathological α-synuclein aggregation remains to be further validated. Second, the expression and function of BCL11B were only verified in cell lines and murine tissues. Human samples, including iPSC-derived neuronal models and postmortem brain tissues from patients with PD, are required to confirm the clinical translational value of BCL11B. Third, the current study only conducted a short-term BCL11B knockdown intervention, and the long-term systemic safety and immune disturbance caused by sustained BCL11B inhibition have not been comprehensively evaluated. Furthermore, although co-immunoprecipitation confirmed the acetylation modification of BCL11B in the MPP^+^-induced cell model, we did not employ a specific acetylated-BCL11B antibody for direct detection. The precise acetylation sites and the functional effects of acetylase inhibitors on BCL11B-mediated neurotoxicity also require further in-depth exploration. Additionally, contrary to the decreased BCL11B expression observed in Huntington’s disease, BCL11B was significantly upregulated in the PD model in our study. Since both PD and HD belong to neurodegenerative disorders, the opposite expression pattern of BCL11B implies its complex and disease-specific regulatory roles in neurodegeneration. Further systematic investigations are necessary to clarify the divergent functions of BCL11B across different degenerative diseases. Collectively, these unresolved limitations should be addressed in our follow-up work to solidify the reliability and translational potential of BCL11B-targeted therapy for PD.

## Resource availability

### Lead contact

Further information and requests for resources should be directed to and will be fulfilled by the lead contact, Jiabing Shen (shenjb0818@ntu.edu.cn).

### Materials availability

AAV vectors, shRNAs, and siRNAs generated or customized for this study are available from the lead contact upon reasonable request.

### Data and code availability

• The RNA-seq datasets are publicly available in the NCBI Sequence Read Archive under BioProject accession：PRJNA1401811. The accession is also listed in the key resources table.
• This paper does not report original code.
• Any additional information required to reanalyze the data reported in this paper is available from the lead contact upon reasonable request.

## Acknowledgments

This work was supported by the following funding sources: the National Natural Science Foundation of China: no. 81901195, the 2024 Natural Science Foundation project of Nantong (JC2024088), the Jiangsu Provincial Research Hospital (YJXYY202204), and the Nantong Municipal Natural Science Foundation Youth Fund Project (JC2023052). We extend our sincere gratitude to Dr. Liu Mei and Dr. Xue Chengbin for assistance with the experiments. We thank the support of the National Natural Science Foundation of China.

## Author contributions

X.G. and J.S. conceived and designed the study; W.C., Y.Z., and X.Z. performed the experiments; J.Y. and Y.S. conducted data analysis; J.S. provided essential reagents, materials, and analytical tools; W.C. and X.G. wrote the manuscript. All authors reviewed and approved the final version.

## Declaration of interests

The authors declare no competing interests.

## STAR★Methods

### Key resources table

**Table:** 

REAGENT or RESOURCE	SOURCE	IDENTIFIER
**Antibodies**		

Rabbit anti-BCL11B	Proteintech	Cat# 55414-1-AP; RRID: AB_11182609
Mouse anti-TH for immunofluorescence	Santa Cruz Biotechnology	Cat# sc-25269; RRID: AB_2877266
Anti-HDAC1	ABclonal	Cat# A26492; RRID:AB_3755950
Anti-HDAC2	ABclonal	Cat# A1163; RRID:AB_2758649
HDAC3 Rabbit mAb	ABclonal	Cat# A19537; RRID: AB_2862654
Tyrosine Hydroxylase Antibody for western blot	Cell Signaling Technology	Cat# 2792; RRID: AB_2115556
Anti-α-synuclein	Cell Signaling Technology	Cat# 4179; RRID:AB_219928
Anti-JAK2	Cell Signaling Technology	Cat# 3230; RRID:AB_2128522
Anti-phospho-JAK2	Cell Signaling Technology	Cat# 3771; RRID:AB_330403
Anti-STAT3	Cell Signaling Technology	Cat# 12640; RRID:AB_2629499
Anti-phospho-STAT3	Cell Signaling Technology	Cat# 9145; RRID:AB_2491009
HRP-conjugated goat anti-rabbit IgG	Proteintech	Cat# SA00001-2; RRID: AB_2491009
HRP-conjugated goat anti-mouse IgG	Proteintech	Cat# SA00001-1; RRID: AB_2722565
Pan Acetylation Monoclonal Antibody	Proteintech	Cat# 66289-1-Ig; RRID: AB_2881672
Normal mouse IgG control	Proteintech	Cat# B900620; RRID:AB_2883054
NeuN Recombinant Mouse Monoclonal Antibody	HUABIO	Cat# HA601111; RRID: AB_3071833
IBA1 Polyclonal Antibody	Proteintech	Cat# 10904-1-AP; RRID: AB_2224377
GFAP Monoclonal Antibody	Proteintech	Cat# 60190-1-Ig; RRID: AB_10838694
Fluorescent secondary antibody for rabbit primary antibodies	Thermo Fisher Scientific	Cat# A-11008; RRID: AB_143165
Fluorescent secondary antibody for mouse primary antibodies	Thermo Fisher Scientific	Cat# A-11005; RRID: AB_2534073
GAPDH Monoclonal Antibody	Proteintech	Cat# 60004-1-Ig; RRID: AB_2107436

**Bacterial and virus strains**		

AAV2/9-hSyn-BCL11B shRNA viral vector	OBiO Technology (Shanghai) Corp., Ltd.	Project/order ID: AOV062; target sequence: 5′-GCTGACCACAGAGTTCAATTT-3′; titer: 1.0 × 10^12^ vg/mL
AAV2/9-hSyn-control shRNA viral vector	OBiO Technology (Shanghai) Corp., Ltd.	Control sequence: 5′-GCTTACGATAGTCGATATT-3′; titer: 1.0 × 10^12^ vg/mL

**Chemicals, peptides, and recombinant proteins**		

MPTP hydrochloride	MedChemExpress	Cat# HY-15608
MPP^+^	MedChemExpress	Cat# HY-W008719
Lipopolysaccharide from Escherichia coli O55:B5	MedChemExpress	Cat# HY-D1056
Poly-D-lysine	Gibco	Cat# A3890401
TRIzol reagent	Thermo Fisher Scientific	Cat# 15596026

**Critical commercial assays**		

TUNEL assay kit	Beyotime	Cat# C1086
EdU assay kit	Beyotime	Cat# C1089
CCK-8 assay kit	NCM Biotech	Cat# C6005
KC Digital mRNA Library Prep Kit	Seqhealth Technology	Cat# KC-DSM
HiScript III RT SuperMix for qPCR	Vazyme	Cat# R323
SYBR Green qPCR kit	Vazyme	Cat# Q711
siRNA/miRNA Transfection Reagent	MedChemExpress	Cat# HY-K2017

**Deposited data**		

Mouse substantia nigra RNA-seq data, MPTP versus sham	This paper; NCBI Sequence Read Archive	BioProject: PRJNA1401811; Study: SRP661562; Runs: SRR36790816–SRR36790821
MN9D RNA-seq data, Si-NC + CM versus Si-NC	This paper; NCBI Sequence Read Archive	BioProject: PRJNA1401811; Study: SRP661562; Runs: SRR36790814–SRR36790815

**Experimental models: Cell lines**		

Mouse: MN9D dopaminergic cell line	Cell Bank of Chinese Academy of Sciences	Cat# C0169; RRID: CVCL_M067
Mouse: BV2 microglial cell line	Cell Resource Center, Shanghai Institute of Cell Biology, Chinese Academy of Sciences	Cat# 0356; RRID: CVCL_0182

**Experimental models: Organisms/strains**		

Mouse: C57BL/6J, wild-type, male, 8 weeks old	Animal Facility of Nantong University	N/A

**Oligonucleotides**		

qPCR primers and BCL11B siRNAs used in this study	This paper; Nanjing Juxionghua Biotechnology Co., Ltd.	See Table S1

**Software and algorithms**		

ImageJ v1.6.0	National Institutes of Health	https://imagej.net/ij/; RRID: SCR_003070
GraphPad Prism v8.5	GraphPad Software	https://www.graphpad.com/scientific-software/prism/; RRID: SCR_002798
fastp v0.23.4	OpenGene	https://github.com/OpenGene/fastp
STAR v2.7.10a	STAR project	https://github.com/alexdobin/STAR
Subread/featureCounts v2.0.3	Subread project	https://subread.sourceforge.net/
DESeq2 v1.38.3	Bioconductor	https://bioconductor.org/packages/DESeq2
clusterProfiler v4.6.2	Bioconductor	https://bioconductor.org/packages/clusterProfiler
R v4.3.1	R Foundation for Statistical Computing	https://www.r-project.org/; RRID: SCR_001905
ggplot2 v3.4.4	CRAN	https://cran.r-project.org/package=ggplot2
Ingenuity Pathway Analysis (IPA)	QIAGEN	Release 01–2024; RRID: SCR_008653
EthoVision XT 15	Noldus Information Technology	https://www.noldus.com/ethovision-xt; RRID: SCR_000481
Mus musculus reference genome and annotation, GRCm38, mm10	Genome Reference Consortium; Ensembl	GRCm38, mm10; Ensembl release 109

**Other**		

RNA-sequencing instrument	Illumina	NovaSeq 6000
ABI PRISM 7500 real-time PCR system	Applied Biosystems	Model 7500
Fluorescence microscope	Leica Microsystems	DMI6000 B
Rotarod apparatus	Ugo Basile	N/A

### Experimental model and study participant details

#### MN9D cells

MN9D cells (mouse dopaminergic cell line, female, Cat# C0169, RRID: CVCL_M067, Cell Bank of Chinese Academy of Sciences, Shanghai, China) were cultured in F12 medium supplemented with 10% fetal bovine serum and 25 μg/mL poly-D-lysine at 37°C in a humidified atmosphere with 5% CO2. The cells were not authenticated and were not tested for mycoplasma contamination in this study. Cells at passages 10–15 were used.

#### BV2 cells

BV2 cells (mouse microglial cell line, female, Cat# 0356, RRID: CVCL_0182, Cell Resource Center, Shanghai Institute of Cell Biology, Chinese Academy of Sciences, Shanghai, China) were cultured in DMEM supplemented with 10% fetal bovine serum and 25 μg/mL poly-D-lysine at 37°C in a humidified atmosphere with 5% CO2. The cells were not authenticated and were not tested for mycoplasma contamination in this study. Cells at passages 10–15 were used.

#### C57BL/6J mice

All male C57BL/6J mice were 8 weeks old and weighed 22–26 g. The mice were obtained from the Animal Facility of Nantong University and housed under specific pathogen-free conditions with a 12-h light/dark cycle, at 22 ± 2°C and 55 ± 5% humidity, with *ad libitum* access to food and water. All experimental protocols were approved by the Institutional Animal Care and Use Committee of Nantong University (approval number S20251219-001) and were conducted in accordance with the Guide for the Care and Use of Laboratory Animals, 8th edition. Only male mice were used. The effect of sex on the study outcomes was not evaluated.

### Method details

#### MN9D cell model *in vitro*

(1) Control group: Cells without any additional treatment.
(2) MPP^+^ group: Cells were exposed to 100 μM MPP^+^ for 12 h to induce cellular injury.
(3) Si-NC group: Cells were transfected with control siRNA using siRNA/miRNA Transfection Reagent (MedChemExpress, Cat# HY-K2017) for 60 h and then maintained in complete F12 medium for another 12 h.
(4) Si-BCL11B group: Cells were transfected with BCL11B siRNA using siRNA/miRNA Transfection Reagent (MedChemExpress, Cat# HY-K2017) for 60 h and then maintained in complete F12 medium for another 12 h.
(5) Si-NC + CM group: Cells were pre-transfected with appropriate Si-NC transfection complex for 60 h, and then incubated with 75% CM for 12 h.
(6) Si-BCL11b + CM group: Cells were pre-transfected with appropriate Si-BCL11b transfection complex for 60 h, and then incubated with 75% CM for 12 h.

#### Inflammatory conditioned medium

BV2 microglial cells were cultured in DMEM supplemented with 10% FBS, and when the cell confluence reached approximately 80%, the cells were treated with 1 μg/mL LPS for 6 h to induce inflammatory activation. After removal of the LPS-containing medium, cells were incubated with fresh complete DMEM for another 24 h. The culture supernatant was then collected, centrifuged at 12,000 rpm for 20 min at 4°C to eliminate cell debris, sterile-filtered through a 0.22 μm microporous membrane, aliquoted, and stored at −20°C for subsequent experimental use.

#### CCK-8 cell-viability assay

Cell viability was assessed using a CCK-8 assay kit (NCM Biotech, Cat# C6005) according to the manufacturer’s instructions. For MPP^+^ dose selection, MN9D cells were exposed to 10, 50, 100, or 500 μM MPP^+^ for 24 h, with untreated cells as the control. For inflammatory conditioned-medium selection, MN9D cells were incubated for 24 h with medium containing 25%, 50%, 75%, or 100% conditioned medium, with untreated cells as the control. Cell-viability values were normalized to the corresponding control group. Cell-based experiments were independently repeated three times.

#### MPTP-induced mouse model

MPTP (HY-15608, MCE) powder was precisely weighed and freshly dissolved in sterile normal saline to prepare working solution with thorough vortexing. The entire preparation process was performed on ice under light protection to prevent reagent degradation and inactivation. An acute mouse model of PD was established via intraperitoneal injection. Mice in the model group received intraperitoneal MPTP injection at a single dose of 20 mg/kg body weight once every 2 h for 4 consecutive administrations within a single day, yielding a total dosage of 80 mg/kg. Mice in the blank control group were injected with an equal volume of sterile normal saline following identical injection frequency and operational procedures. All mice were maintained under routine feeding conditions after the final injection, and their general conditions were monitored continuously for 7 days. Behavioral assessments were conducted in accordance with the experimental protocol upon completion of model induction.

For the MPTP-induced PD mouse model, animals were randomly assigned to four experimental groups.

(1) AAV-CON group: 1 μL of AAV-CON was stereotaxically injected into each side of the bilateral substantia nigra.
(2) AAV-CON + MPTP group: 1 μL of AAV-CON was stereotaxically injected into each side of the bilateral substantia nigra. Two weeks later, MPTP was administered intraperitoneally at a dose of 20 mg/kg.
(3) AAV-BCL11B shRNA group: 1 μL of AAV-BCL11B shRNA was stereotaxically injected into each side of the bilateral substantia nigra.
(4) AAV-BCL11B shRNA + MPTP group: 1 μL of AAV-BCL11B shRNA was stereotaxically injected into each side of the bilateral substantia nigra. Two weeks later, MPTP was administered intraperitoneally at a dose of 20 mg/kg. AAV2/9 vectors expressing BCL11B-specific shRNA or control shRNA under the hSyn promoter were generated by OBiO Technology (Shanghai) Corp., Ltd. The BCL11B-targeting sequence was 5′-GCTGACCACAGAGTTCAATTT-3′. Viral particles were prepared at a reported titer of 1.0 × 10^12^ vg/mL. Stereotaxic injections were made at anteroposterior −3.3 mm, mediolateral ±1.2 mm, and dorsoventral −4.6 mm relative to bregma.

#### Behavioral assessment

##### Rotarod test

Motor coordination and balance were assessed after the behavioral-training period shown in Figure 6A. The four experimental groups each included eight mice. Each mouse was placed on the rotating rod, and the time and rotation speed at falling, together with the distance traveled before falling, were recorded for analysis.

##### Open-field test (OFT)

The open field test (OFT) was used to assess anxiety-like behavior and spontaneous locomotor activity in rodents. Before testing, all mice were transferred to the quiet experimental room for a 30-min habituation period to acclimate to the ambient temperature (22 ± 1°C) and eliminate stress interference. The test was conducted under uniform dim lighting (30–50 lux) to avoid excessive light-induced anxiety. Briefly, each mouse was gently placed in a square arena (45 × 45 × 30 cm) and allowed to explore freely for 10 min. The movement trajectories were continuously recorded by an overhead video camera. To reduce subjective bias, the behavioral data analysis was performed by an experienced researcher who was blinded to the experimental grouping. Parameters including total distance traveled, distance moved in the center zone, and time spent in the center area were systematically analyzed using EthoVision XT 15 software.

##### Pole-climbing test

The pole test was employed to evaluate bradykinesia in the PD mouse model. Briefly, a vertical pole (55 cm in height, 1 cm in diameter) was used. Each mouse was placed head-up at the top of the pole, and the time required to reorient completely downward (T-turn) was recorded. Each animal underwent three trials, and the mean value was used for statistical analysis.

#### Immunofluorescence staining

Mice were transcardially perfused with phosphate-buffered saline (PBS) followed by 4% paraformaldehyde (PFA) for tissue fixation. The harvested brains were post-fixed in 4% PFA for 24 h, dehydrated, and cryoprotected in 30% sucrose solution until sedimentation. After embedding in Optimal Cutting Temperature (OCT) compound (Sakura, 4583), the frozen brains were sectioned coronally at 10 μm thickness using a cryostat.

For immunofluorescence staining, brain sections and fixed cell cultures were blocked with 5% bovine serum albumin and 0.3% Triton X-100 for 1 h and then incubated with primary antibodies at 4°C overnight. The samples were washed with PBS and incubated with species-appropriate fluorescent secondary antibodies at 37°C for 2 h in the dark. After washing, the samples were mounted with antifade medium containing Hoechst 33342 and imaged using a Leica DMI6000 B fluorescence microscope. Primary antibodies included rabbit anti-BCL11B (Proteintech, Cat# 55414-1-AP), mouse anti-TH (Santa Cruz Biotechnology, Cat# sc-25269), mouse anti-NeuN (HUABIO, Cat# HA601111), rabbit anti-IBA1 (Proteintech, Cat# 10904-1-AP), and mouse anti-GFAP (Proteintech, Cat# 60190-1-Ig). Fluorescent secondary antibodies were from Thermo Fisher Scientific (Cat# A-11008 and A-11005).

#### TUNEL assay

The TUNEL assay was performed using a commercial kit (Beyotime) according to the manufacturer’s instructions. Cells grown on glass coverslips were fixed with 4% paraformaldehyde for 30 min at room temperature and permeabilized for 5 min. After PBS washes, the TUNEL reaction mixture was applied to the samples and incubated at 37°C for 1 h in the dark. Fluorescent signals were visualized and captured using a fluorescence microscope.

#### EdU assay

Cell proliferation was evaluated using the EdU assay kit (Beyotime) following the manufacturer’s protocol. MN9D cells were plated on sterilized glass coverslips in 24-well plates. When cells reached 60–70% confluence, they were subjected to stimulation and drug treatment. A pre-warmed 2× EdU working solution was added to each well and incubated for 2 h. After removal of the medium, cells were fixed at room temperature. A freshly prepared Click reaction mixture was then applied and incubated for 30 min in the dark. Finally, nuclei were counterstained with Hoechst 33342-containing anti-fade mounting medium, and images were acquired using a Leica DMI6000 B fluorescence microscope.

#### Western blot analysis

Protein extracts from cells and brain tissues were prepared using lysis buffer, and protein concentrations were measured with a BCA assay kit. Total protein, 20–40 μg per lane, was separated by 8–10% SDS-PAGE and transferred to PVDF membranes. After blocking with 5% non-fat milk for 2 h at room temperature, the membranes were incubated with primary antibodies overnight at 4°C. The antibodies included BCL11B (Proteintech, Cat# 55414-1-AP), HDAC1 (ABclonal, Cat# A26492), HDAC2 (ABclonal, Cat# A1163), HDAC3 (ABclonal, Cat# A19537), TH (Cell Signaling Technology, Cat# 2792), α-synuclein (Cell Signaling Technology, Cat# 4179), JAK2 (Cell Signaling Technology, Cat# 3230), phospho-JAK2 (Cell Signaling Technology, Cat# 3771), STAT3 (Cell Signaling Technology, Cat# 12640), phospho-STAT3 (Cell Signaling Technology, Cat# 9145), and GAPDH (Proteintech, Cat# 60004-1-Ig). After washing with TBST, membranes were incubated with HRP-conjugated goat anti-rabbit IgG (Proteintech, Cat# SA00001-2) or goat anti-mouse IgG (Proteintech, Cat# SA00001-1) for 2 h at room temperature. Protein bands were visualized using an enhanced chemiluminescence detection system and quantified using ImageJ v1.6.0.

#### Co-immunoprecipitation (Co-IP)

MN9D cells were washed with PBS and lysed in ice-cold PBST (PBS containing 0.5% Triton X-100) supplemented with protease and phosphatase inhibitors. For each immunoprecipitation, 400 μL of whole cell lysate was incubated with 8 μL of anti-pan-acetylation antibody, 4 μL of anti-BCL11B antibody, or 5 μL of normal mouse IgG for 2 h at room temperature. Meanwhile, 40 μL of protein A/G magnetic beads were washed three times with PBST. The pre-cleared beads were then added to the antibody-lysate mixtures and incubated for 2 h at room temperature with gentle rotation. After three washes with PBST, bound proteins were eluted by boiling in SDS loading buffer and subsequently analyzed by western blotting.

#### RNA extraction and sequencing

Total RNA was extracted using TRIzol Reagent (Thermo Fisher Scientific, Cat# 15596026). Residual genomic DNA was removed with DNase I (NEB, Cat# M0303L). RNA purity was evaluated using a NanoDrop OneC spectrophotometer, RNA integrity was assessed using a LabChip GX Touch system, and RNA concentration was measured with a Qubit 3.0 fluorometer and Qubit RNA Broad Range Assay Kit (Thermo Fisher Scientific, Cat# Q10210). Sequencing libraries were prepared using the KC Digital mRNA Library Prep Kit (Seqhealth Technology, Cat# KC-DSM), size-selected for 200–500 bp fragments, quantified, and sequenced on an Illumina NovaSeq 6000 platform in paired-end 150-bp mode. Clean reads were aligned to the mouse reference genome GRCm38, mm10.

Raw RNA-seq reads were processed with fastp v0.23.4 to remove adapters and low-quality reads. Clean reads were aligned to the mouse GRCm38, mm10, reference genome using STAR v2.7.10a. Gene counts were generated with featureCounts in Subread v2.0.3. Differential expression analysis was performed using DESeq2 v1.38.3 in R v4.3.1. Genes with an adjusted *p* value <0.05 and an absolute log2 fold change ≥1.5 were considered differentially expressed. Gene Ontology and KEGG enrichment analyses were performed using clusterProfiler v4.6.2. Volcano plots and heatmaps were generated using ggplot2 v3.4.4.

#### Ingenuity pathway analysis

Differential-expression results from the RNA-seq analyses were examined using Ingenuity Pathway Analysis (QIAGEN, release 01–2024; RRID: SCR_008653). The analysis was used to identify predicted molecular relationships between BCL11B and HDAC family members and to predict factors downstream of BCL11B. IPA outputs were interpreted as in silico predictions; selected relationships were subsequently examined by co-immunoprecipitation, qRT-PCR, or western blotting in the corresponding experiments described in method details.

#### RNA isolation and quantitative PCR

MN9D cells were plated in 6-well plates and treated with 100 μM MPP^+^ for 12 h. Total RNA was extracted using TRIzol reagent and reverse-transcribed using the HiScript III RT SuperMix for qPCR (Vazyme, Cat# R323). Quantitative PCR was performed using an SYBR Green qPCR kit (Vazyme, Cat# Q711) on an ABI PRISM 7500 real-time PCR system. The cycling program was 95°C for 10 s, followed by 40 cycles of 95°C for 5 s and 60°C for 30 s. Relative expression was calculated using the comparative Ct method. Each biological sample was assayed in three technical wells. Primer and siRNA sequences are provided in Table S1.

### Quantification and statistical analysis

Data are presented as mean ± SEM. Animal experiments used eight mice per group unless otherwise stated. Cell-based experiments were independently repeated three times, and qPCR measurements were performed in three technical wells for each biological sample. Comparisons between two groups were performed using an independent two-sample *t* test. Comparisons among multiple groups were performed using one-way ANOVA followed by Sidak’s post hoc test. Analyses were performed using GraphPad Prism v8.5. *p* values <0.05 were considered statistically significant. Animals were randomly assigned to experimental groups, and open-field data were analyzed by an investigator blinded to group allocation. Where statistical tests are presented as asterisks, ∗ indicates *p* < 0.05, ∗∗ indicates *p* < 0.01, ∗∗∗ indicates *p* < 0.001, and ∗∗∗∗ indicates *p* < 0.0001.

## Supplemental information

Data S1. Unedited microscopy images from at least two independent experiments, related to Figures 2, 5, and 7 (included in the Supplemental PDF)

Data S2. Uncropped western blot images from at least two independent experiments, related to Figures 2, 3, 4, 5, 7, and 8 (included in the Supplemental PDF)

Table S1. Primer and siRNA sequences used in this study, related to METHOD DETAILS (separate Excel file)
