# Supplementary material for: Targeting BCL11B-mediated JAK2/STAT3 activation: A novel therapeutic strategy for Parkinson’s disease
Source: iScience. 2026 Oct 8;29(11):117833. doi: 10.1016/j.isci.2026.117833 (PMC13677406; doi:10.1016/j.isci.2026.117833)

iScience, Volume *29*

Supplemental information

Targeting BCL11B-mediated JAK2/STAT3 activation: A novel therapeutic strategy for Parkinson's disease

Wenwen Chen, Yejing Zhang, Xinrui Zhou, Jianan Yan, Yiming Song, Jiabing Shen, and Xiaosu Gu

Figure2 A n = 3 independent replicates

Replicate 1

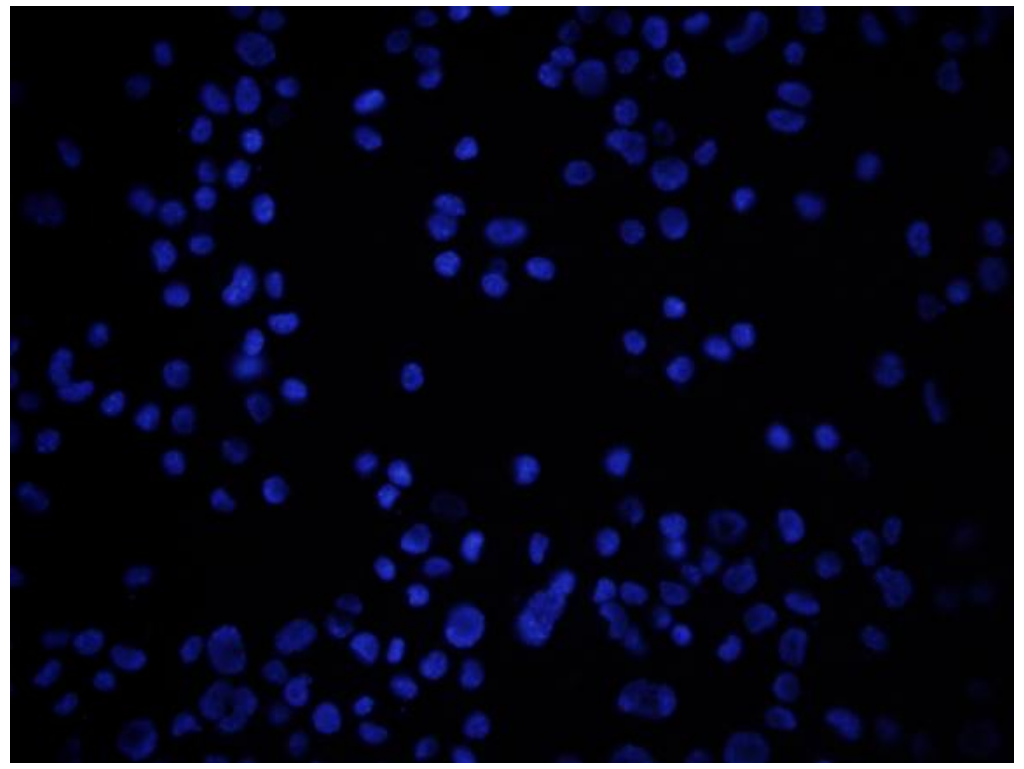

Be showed in the figure

Blue： Hochest

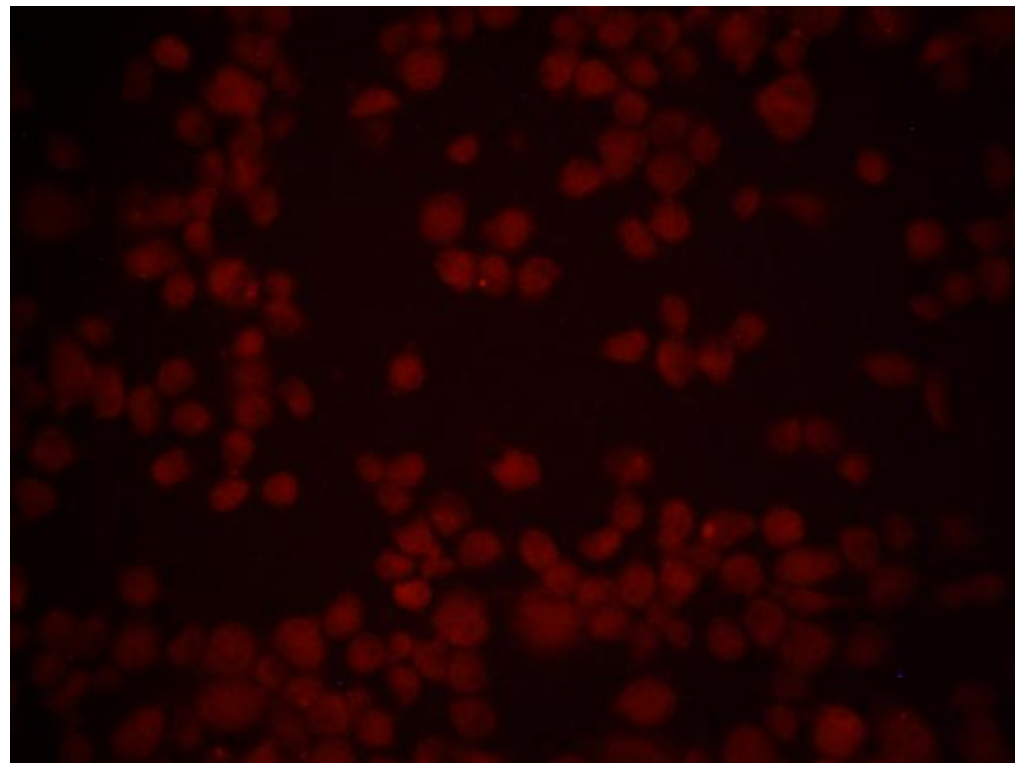

Be showed in the figure

Red： TH

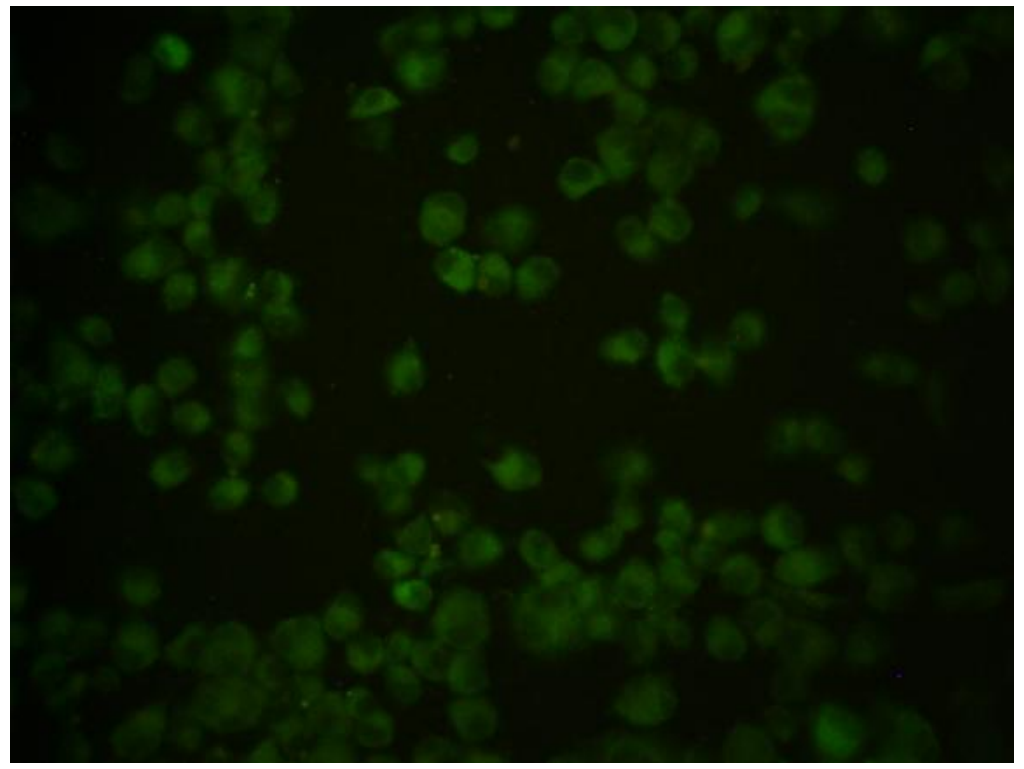

Be showed in the figure

Green： BCL11b

Replicate 2

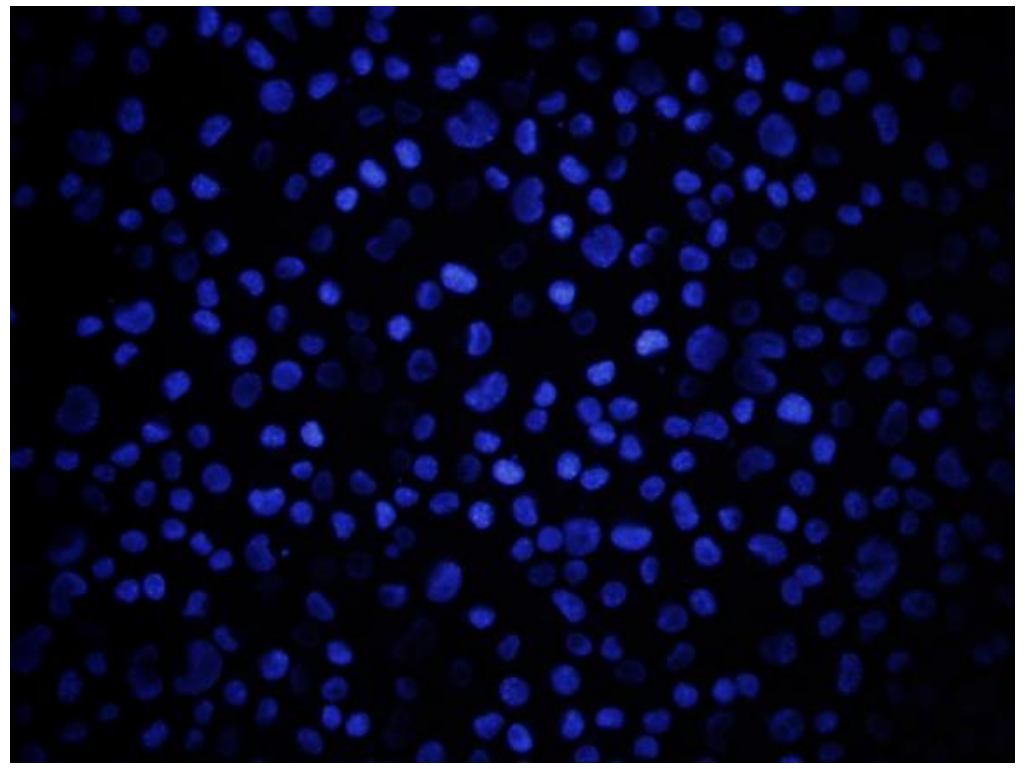

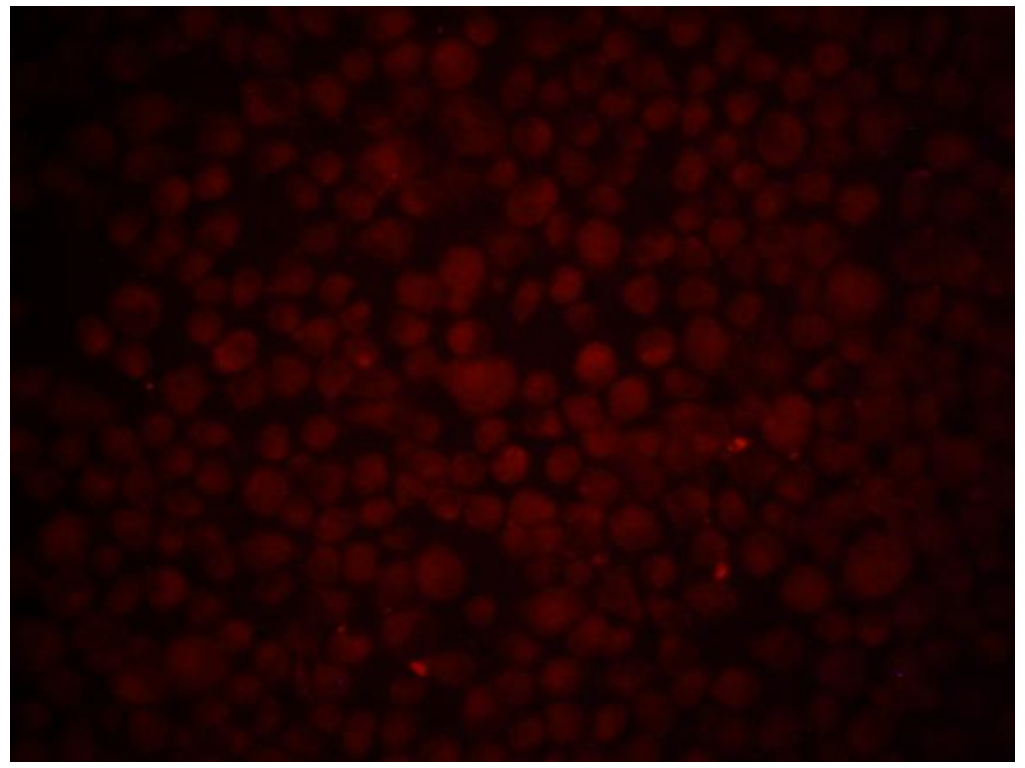

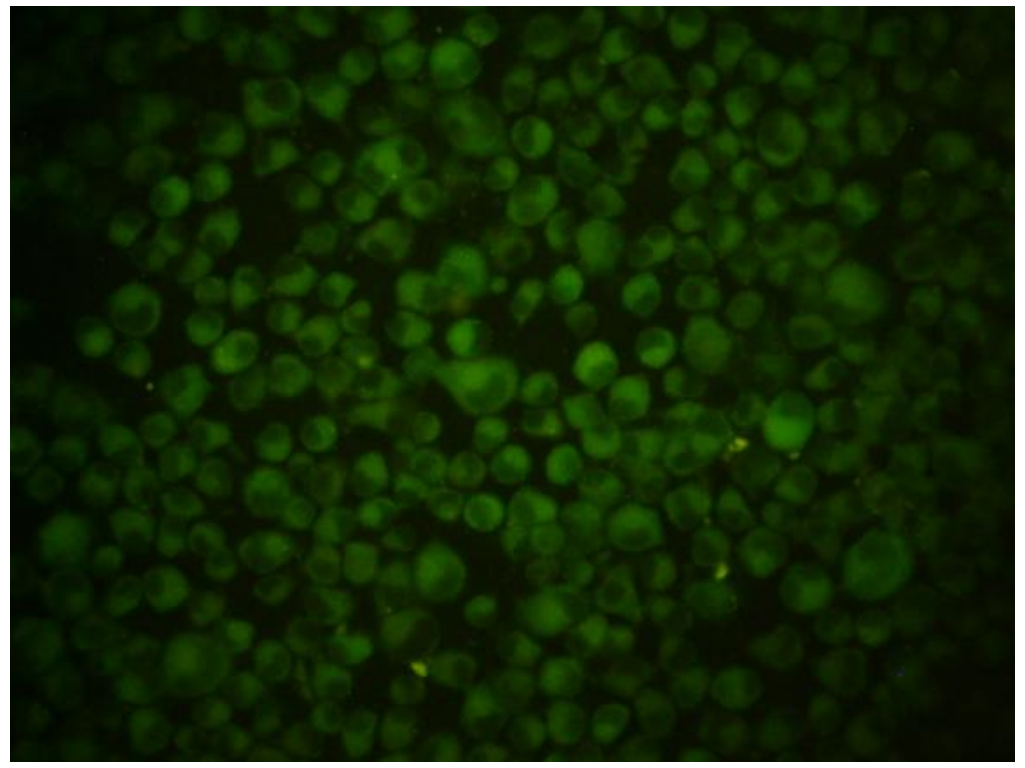

Figure2 H

n = 3 independent replicates

Replicate 1

Replicate 2

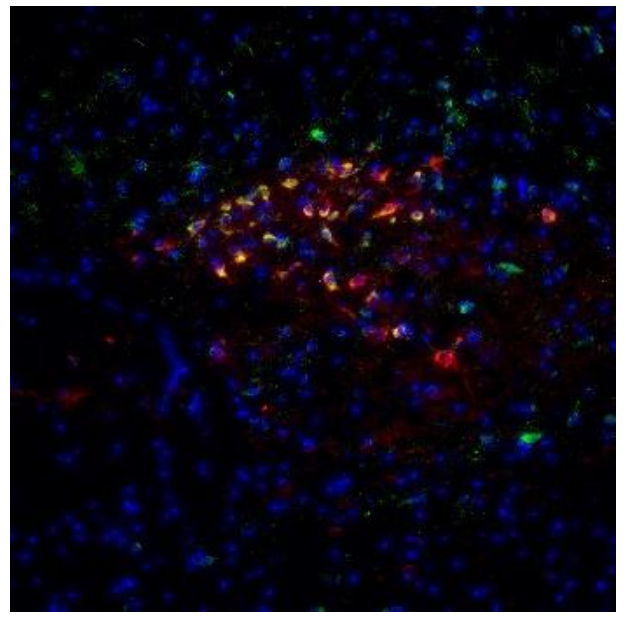

Be showed in the figure

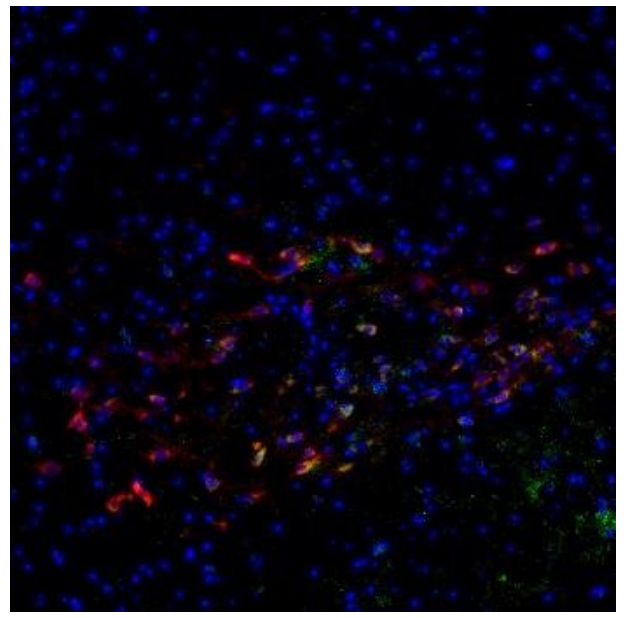

Blue： Hochest

Red： TH

Green： BCL11b

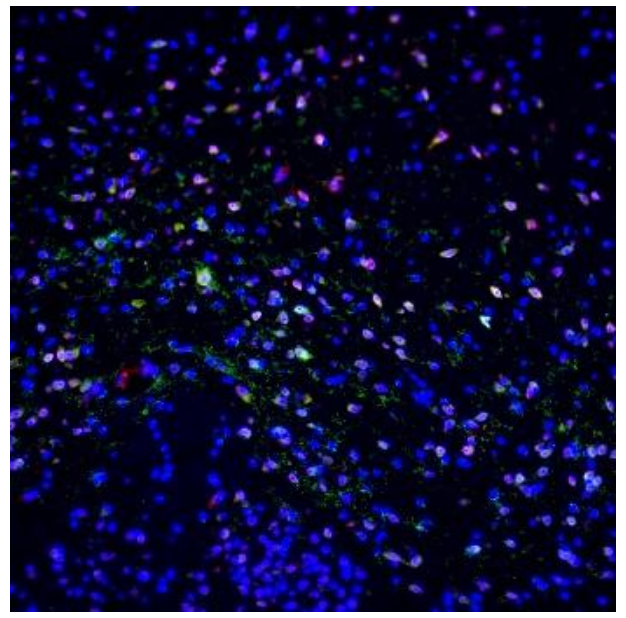

Be showed in the figure

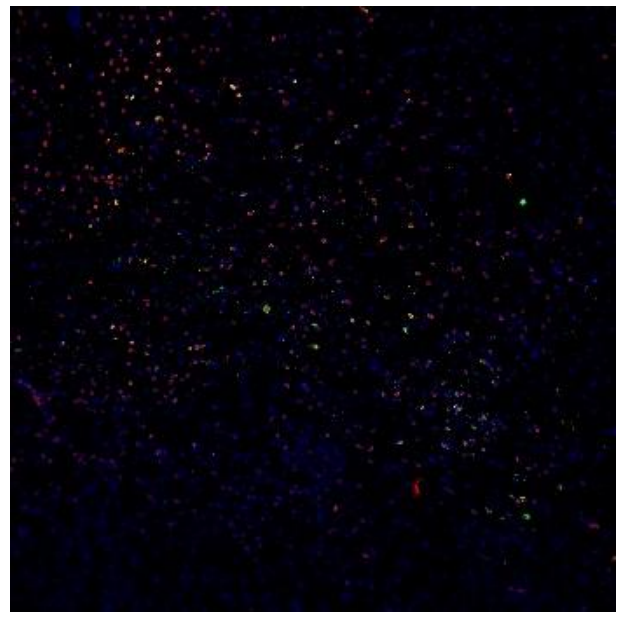

Blue： Hochest

Red： Neun

Green： BCL11b

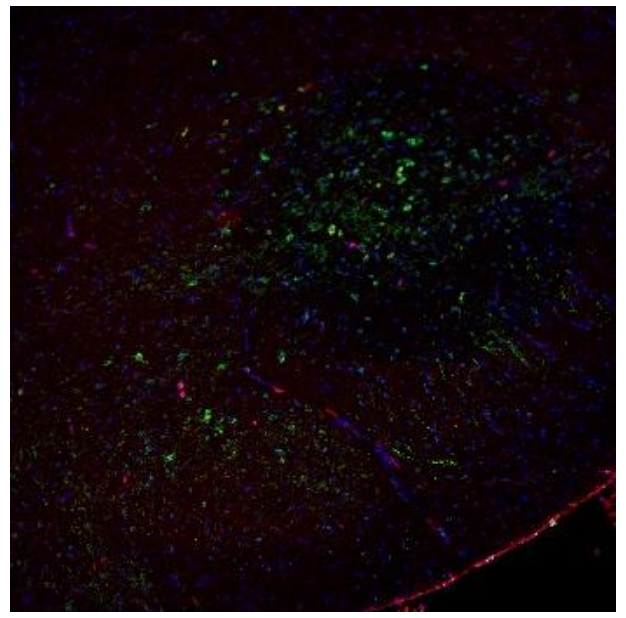

Be showed in the figure

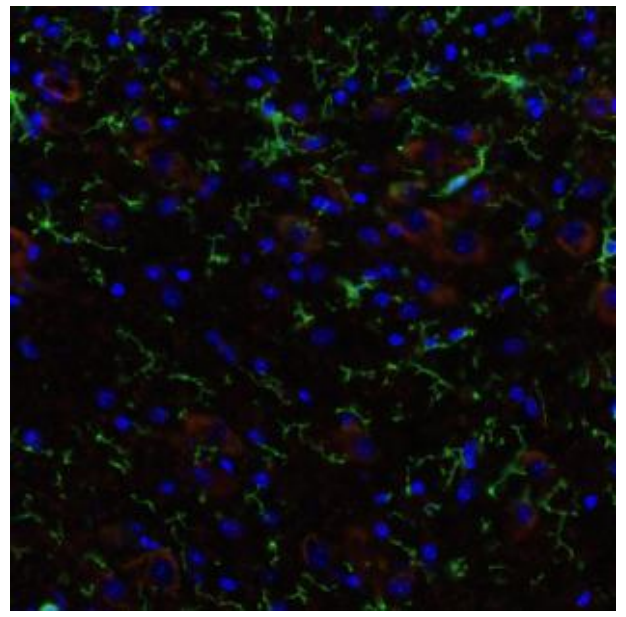

Blue： Hochest

Red： Bcl11b

Green： Iba1

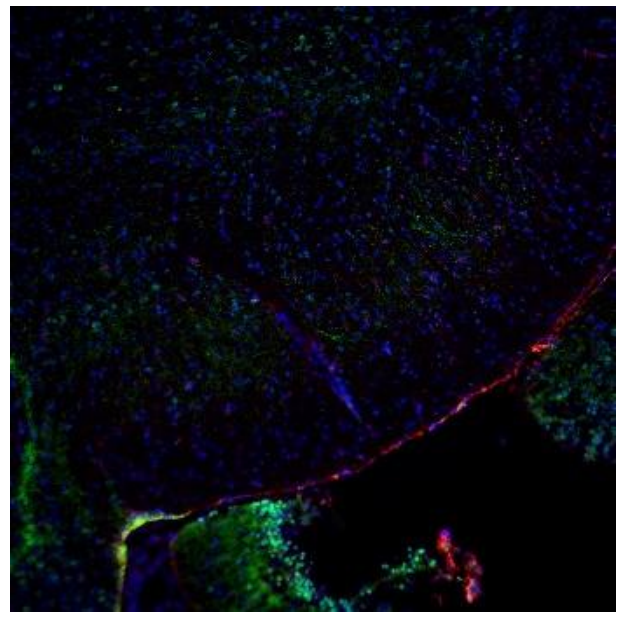

Be showed in the figure

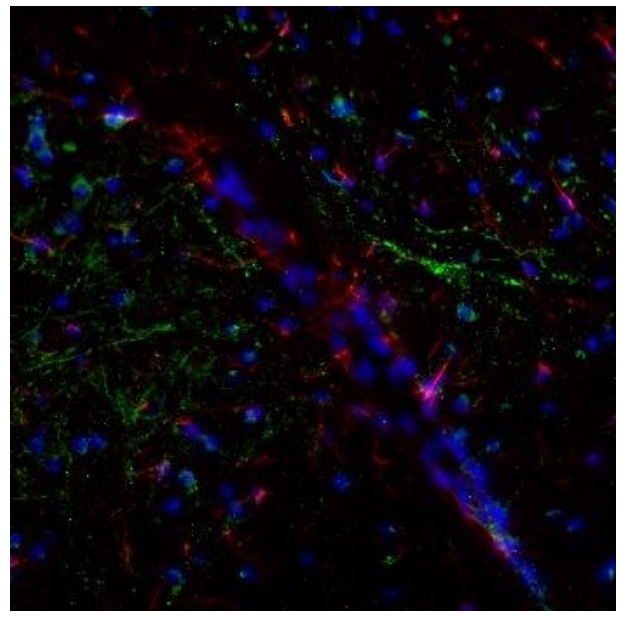

Blue： Hochest

Red： GFAP

Green： BCL11b

Figure5 E

n = 3 independent replicates

Blue： Hochest

Green： TUNEL

Replicate 1

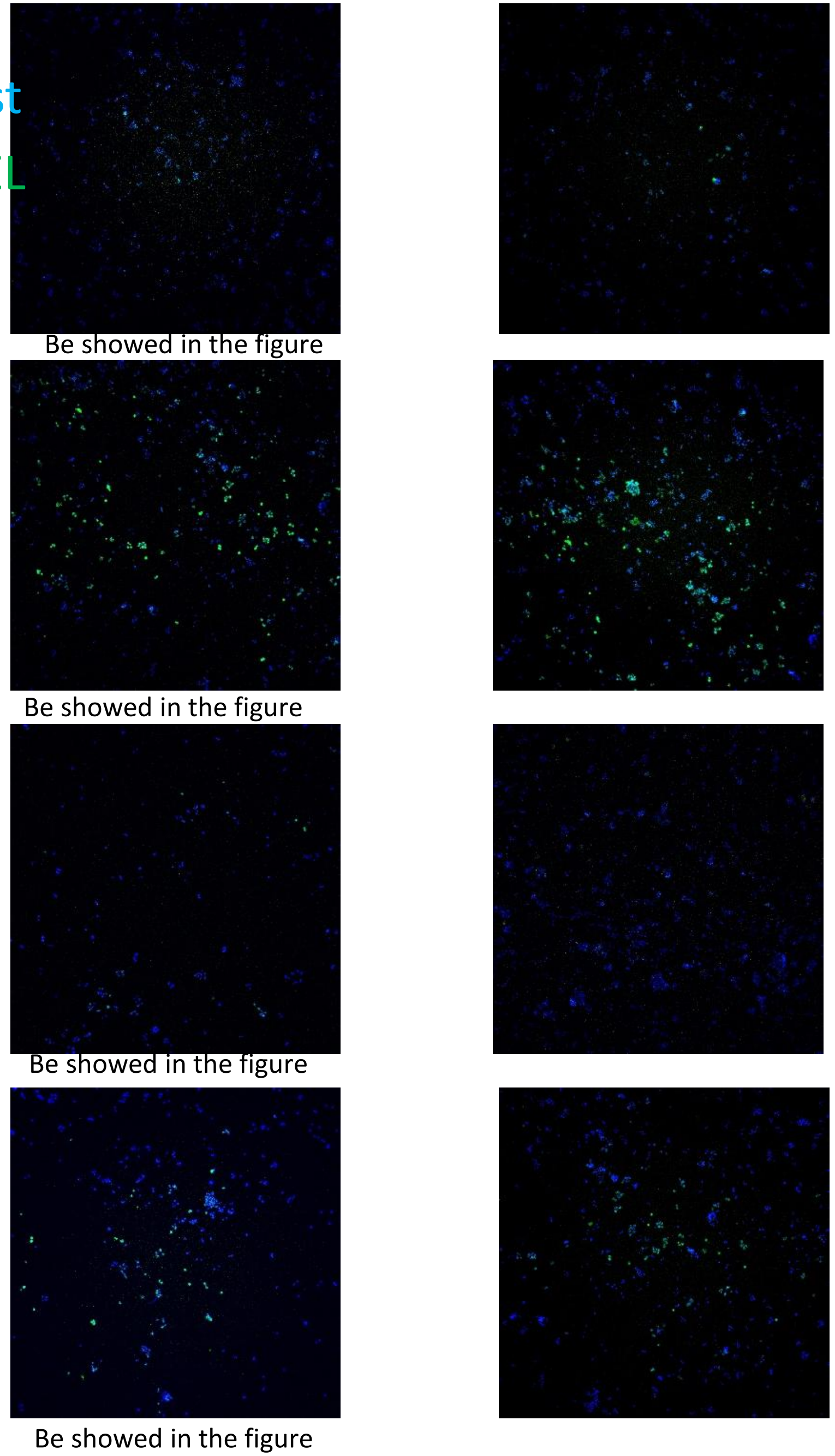

Replicate 2

Replicate 1

Replicate 2

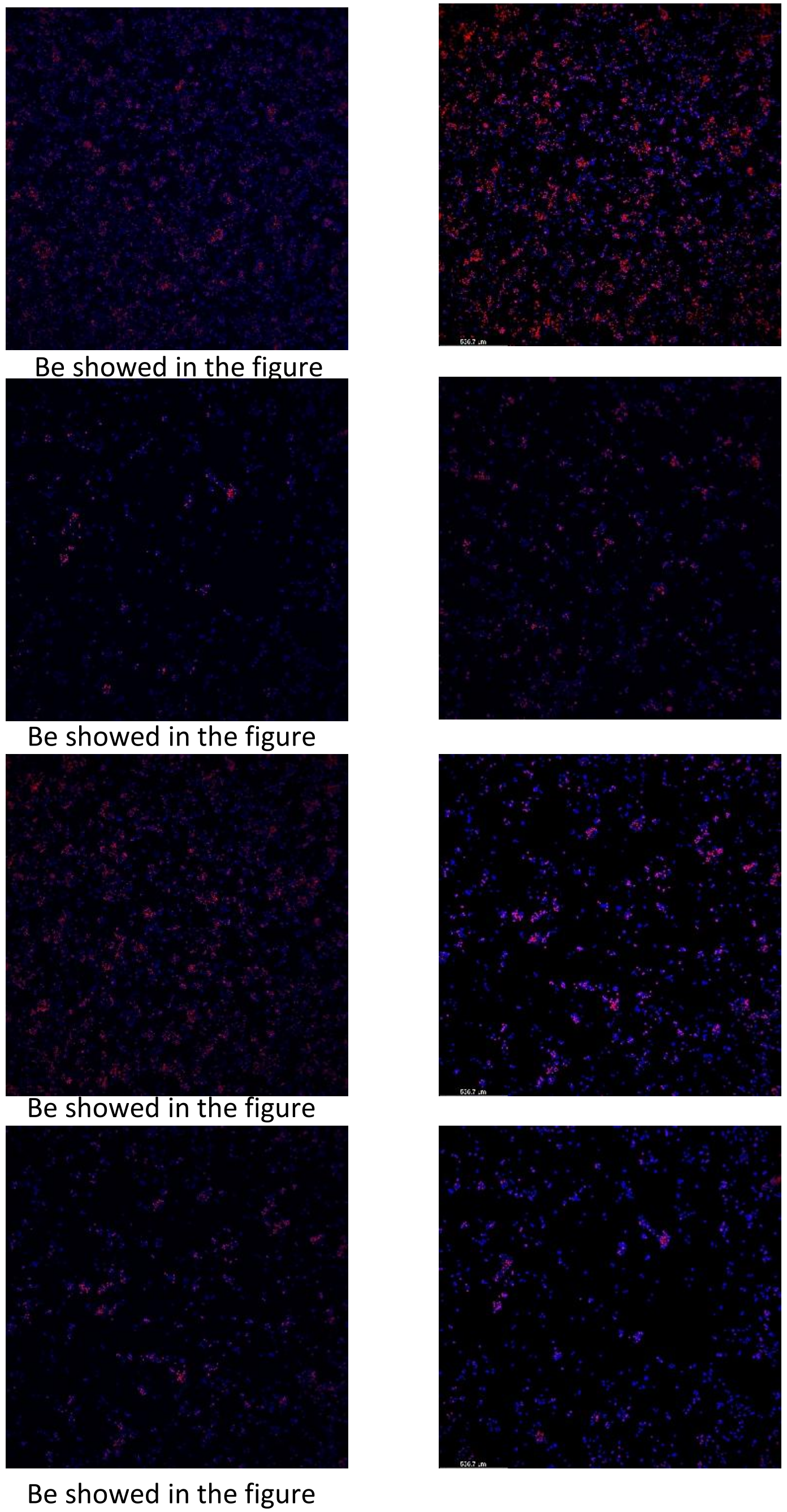

Blue： Hochest

Red： EDU

Figure7 C

n = 3 independent replicates

Blue： Hochest

Red： TH

Green： BCL11b

Replicate 1

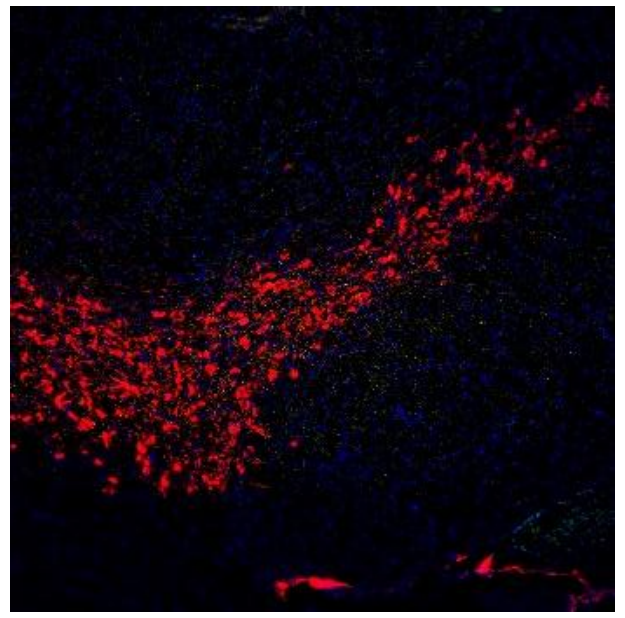

Be showed in the figure

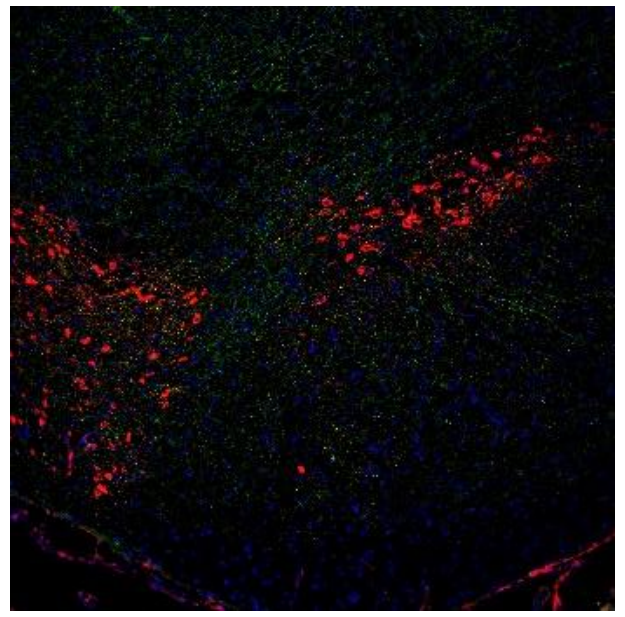

Be showed in the figure

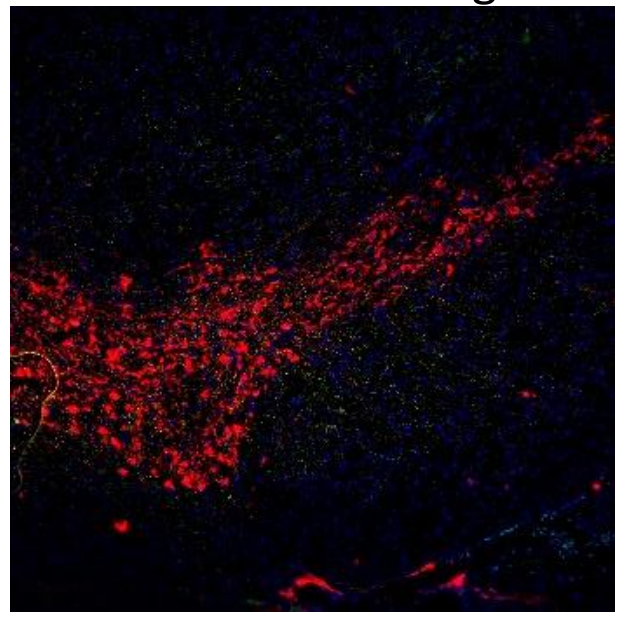

Be showed in the figure

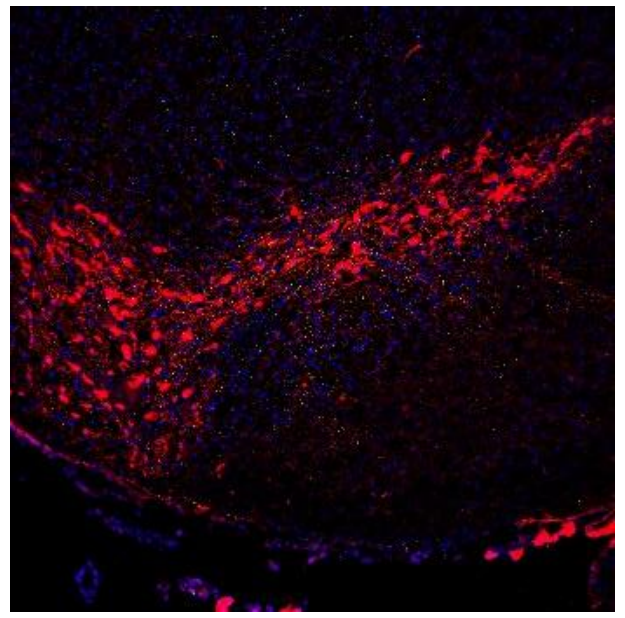

Be showed in the figure

Replicate 2

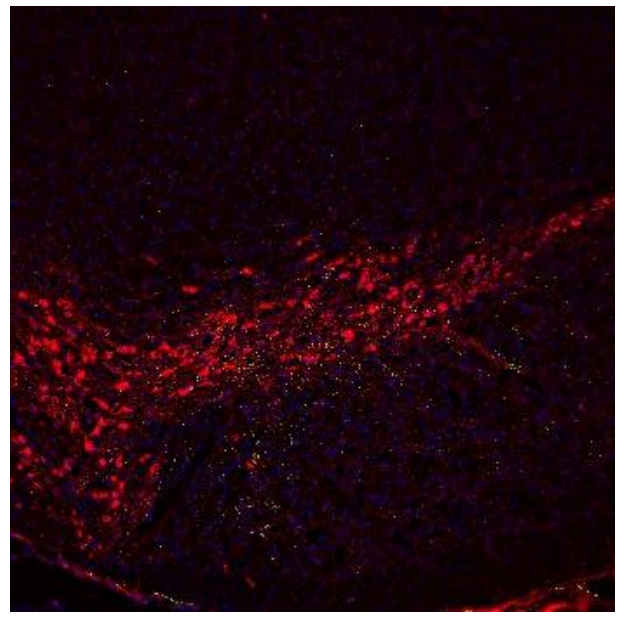

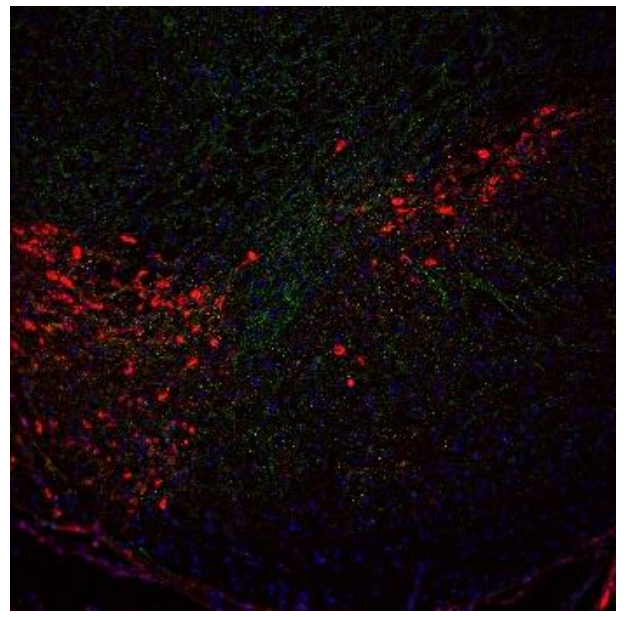

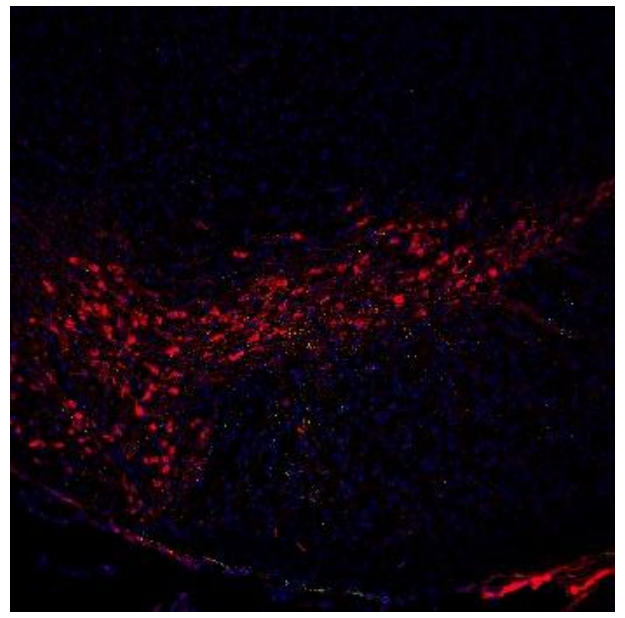

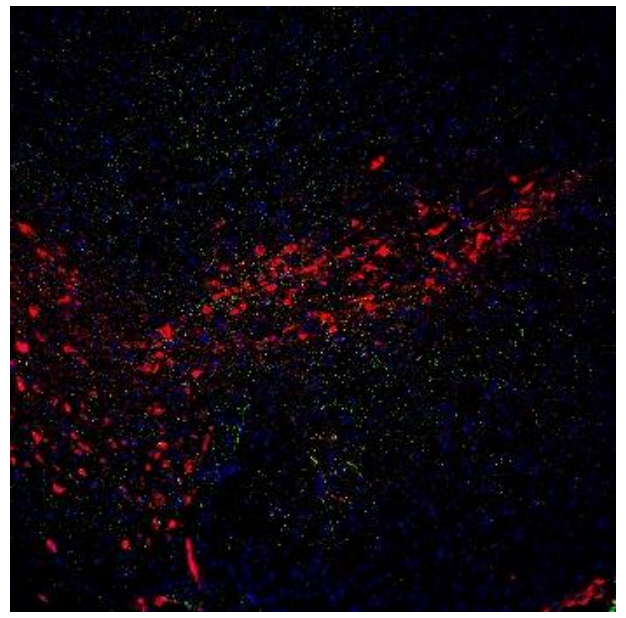

Supplement: Supplementary file 1 [file mmc1.pdf]
