# Supplementary material for: Targeting BCL11B-mediated JAK2/STAT3 activation: A novel therapeutic strategy for Parkinson’s disease
Source: iScience. 2026 Oct 8;29(11):117833. doi: 10.1016/j.isci.2026.117833 (PMC13677406; doi:10.1016/j.isci.2026.117833)

Figure2 D n = 3 independent replicates

replicates 1

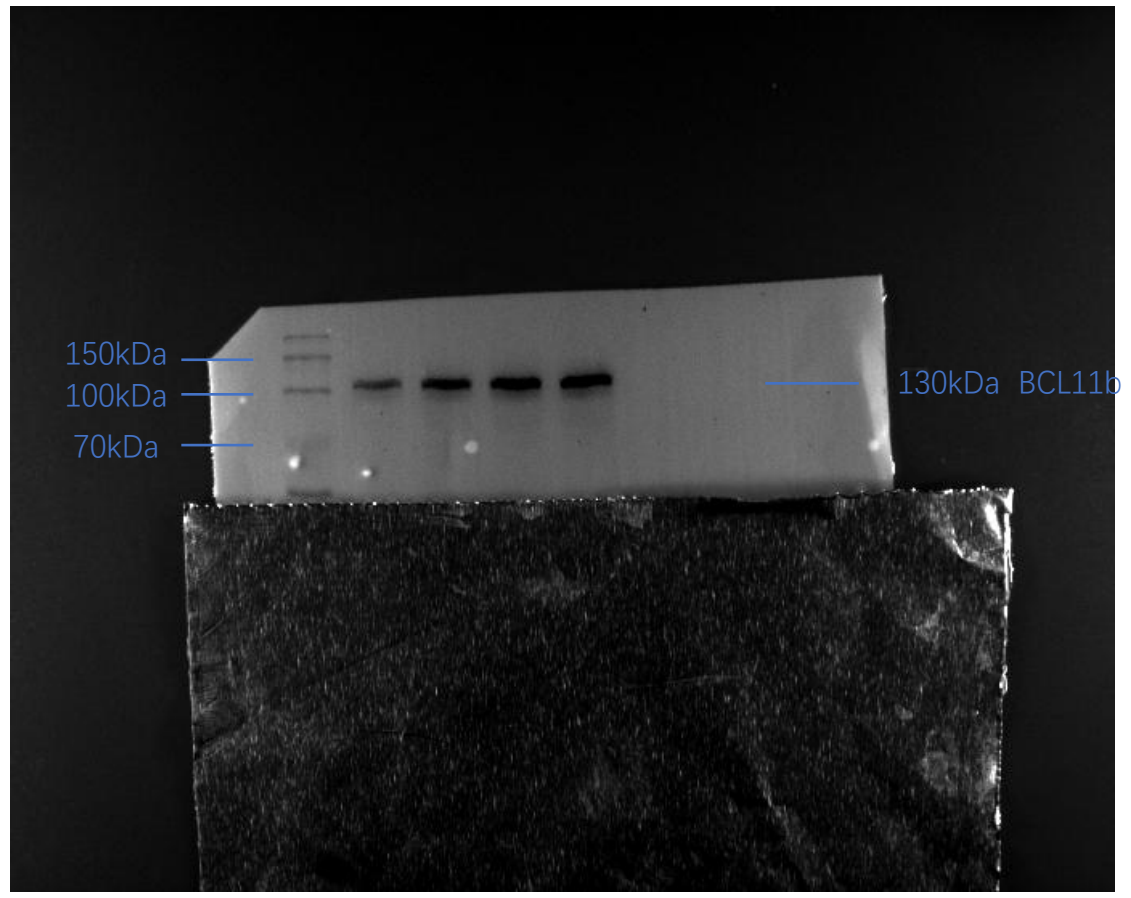

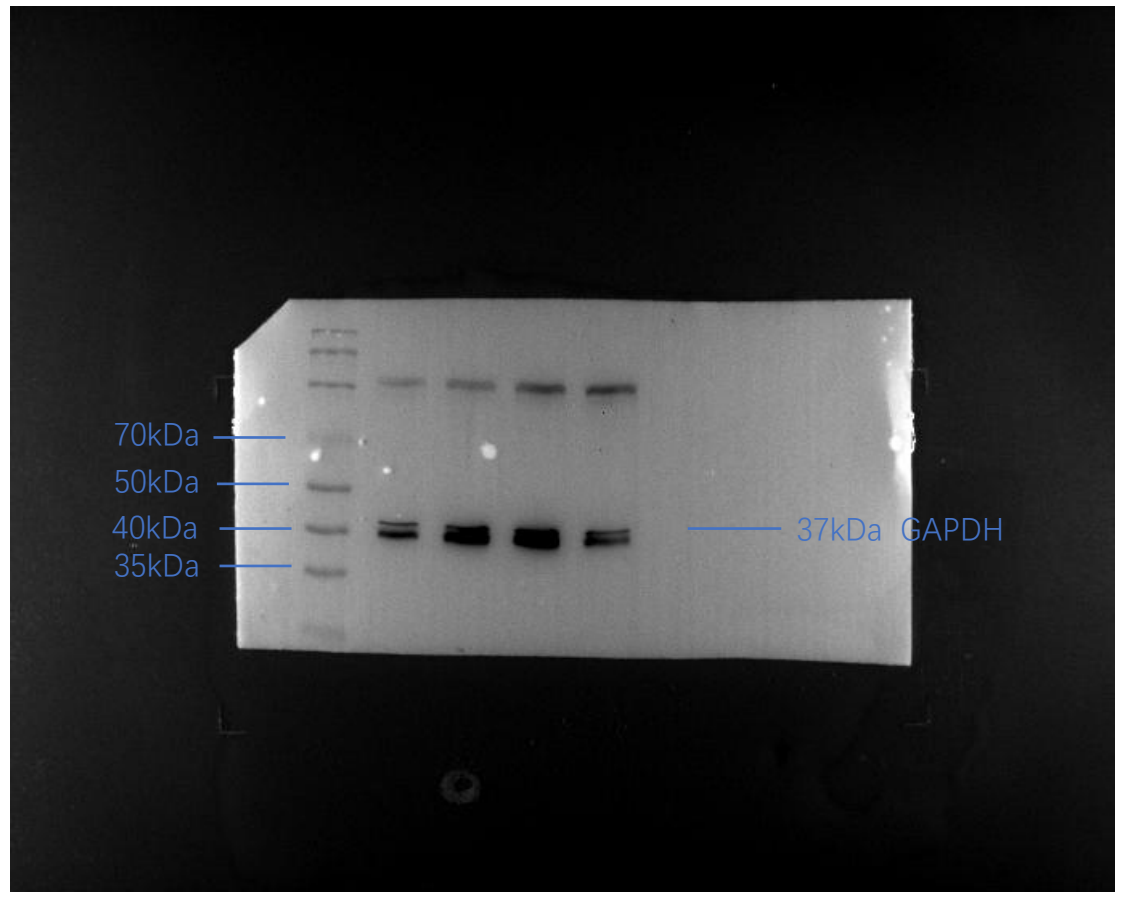

replicates 2

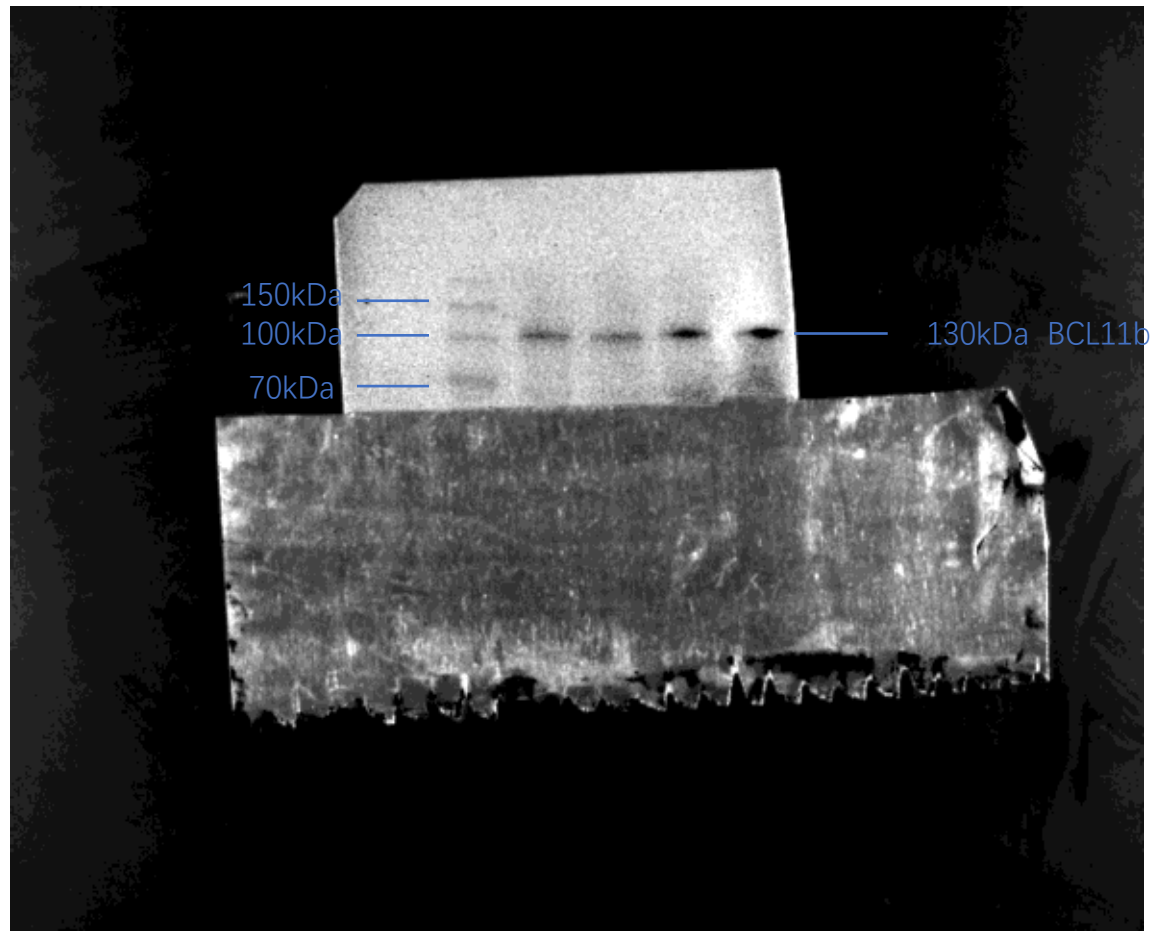

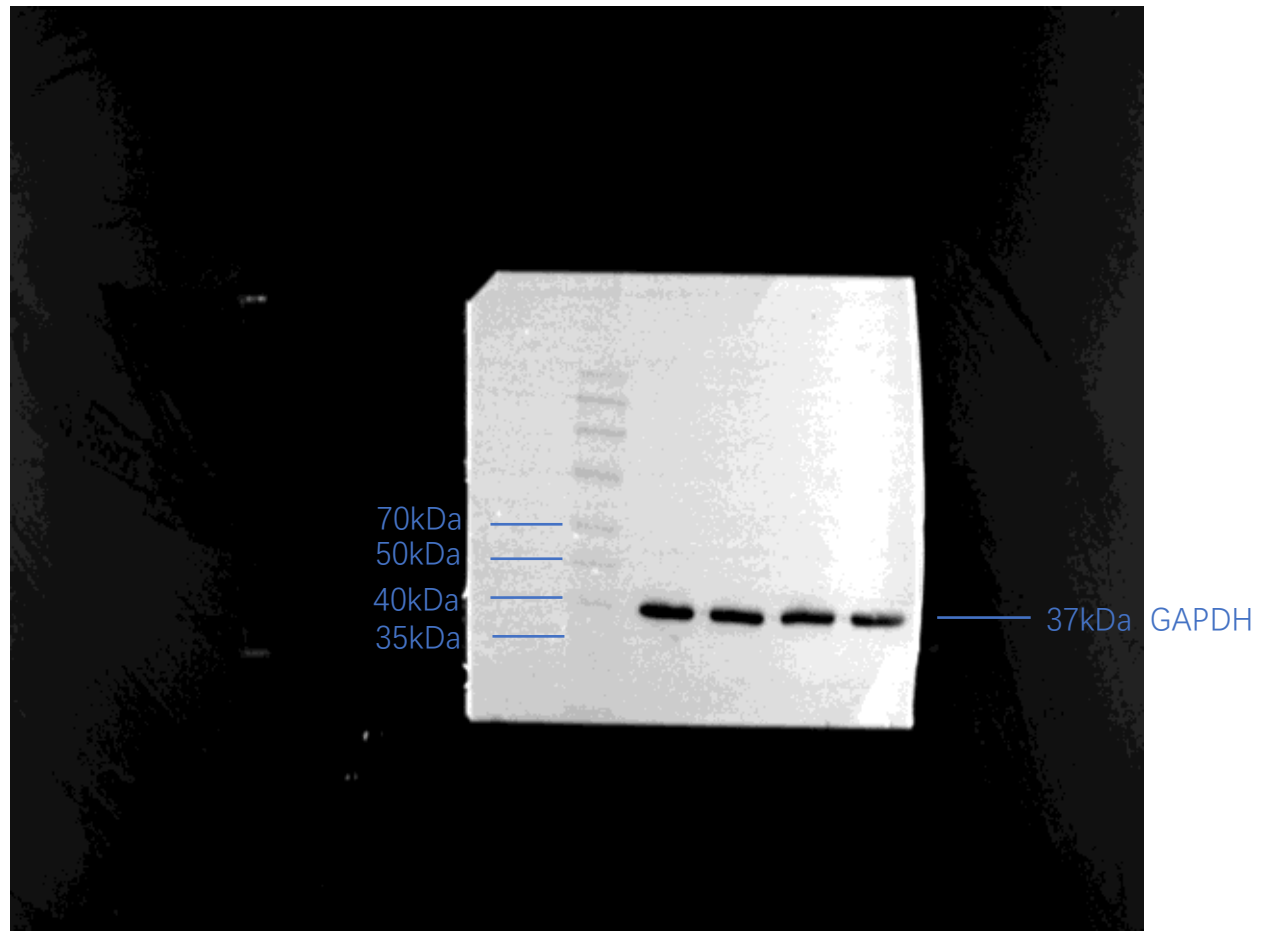

Be showed in the figure

Be showed in the figure

Figure2 F   n = 3 independent replicates

replicates 1   replicates 2   replicates 1   replicates 2   replicates 3

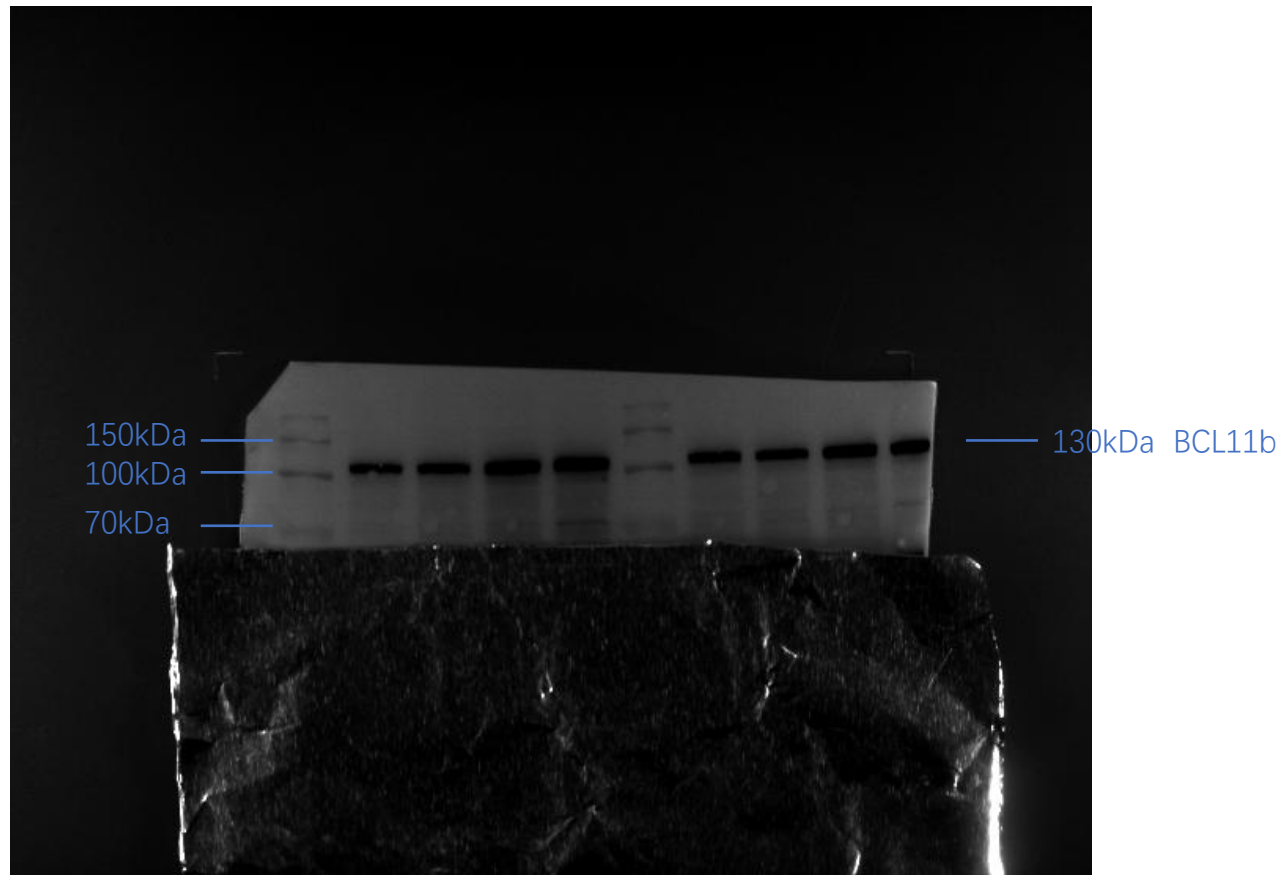

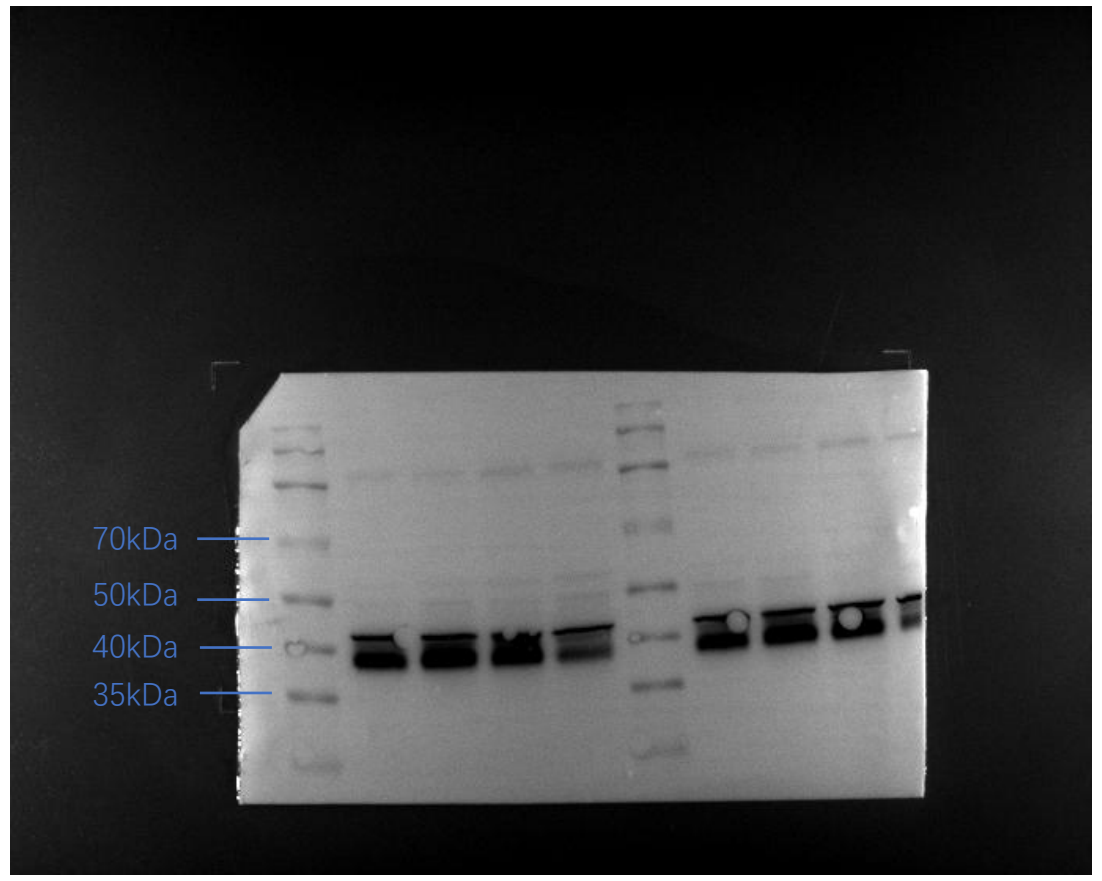

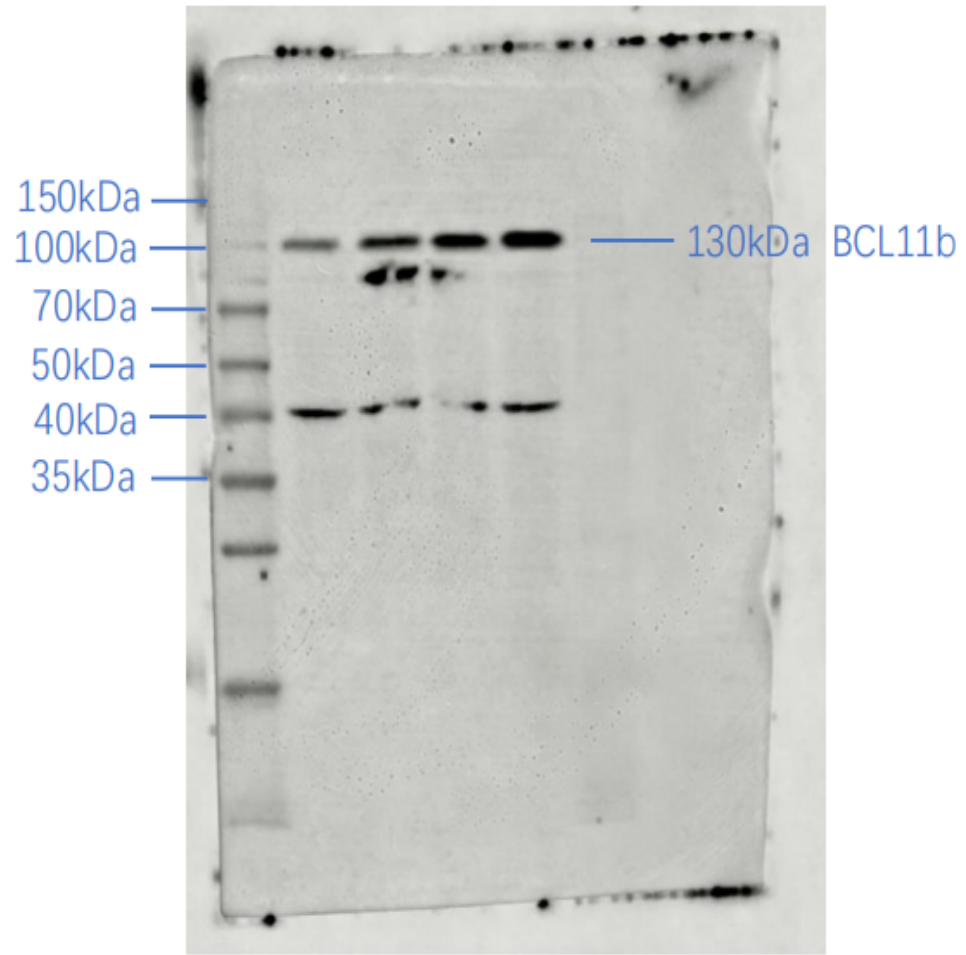

Be showed in the figure

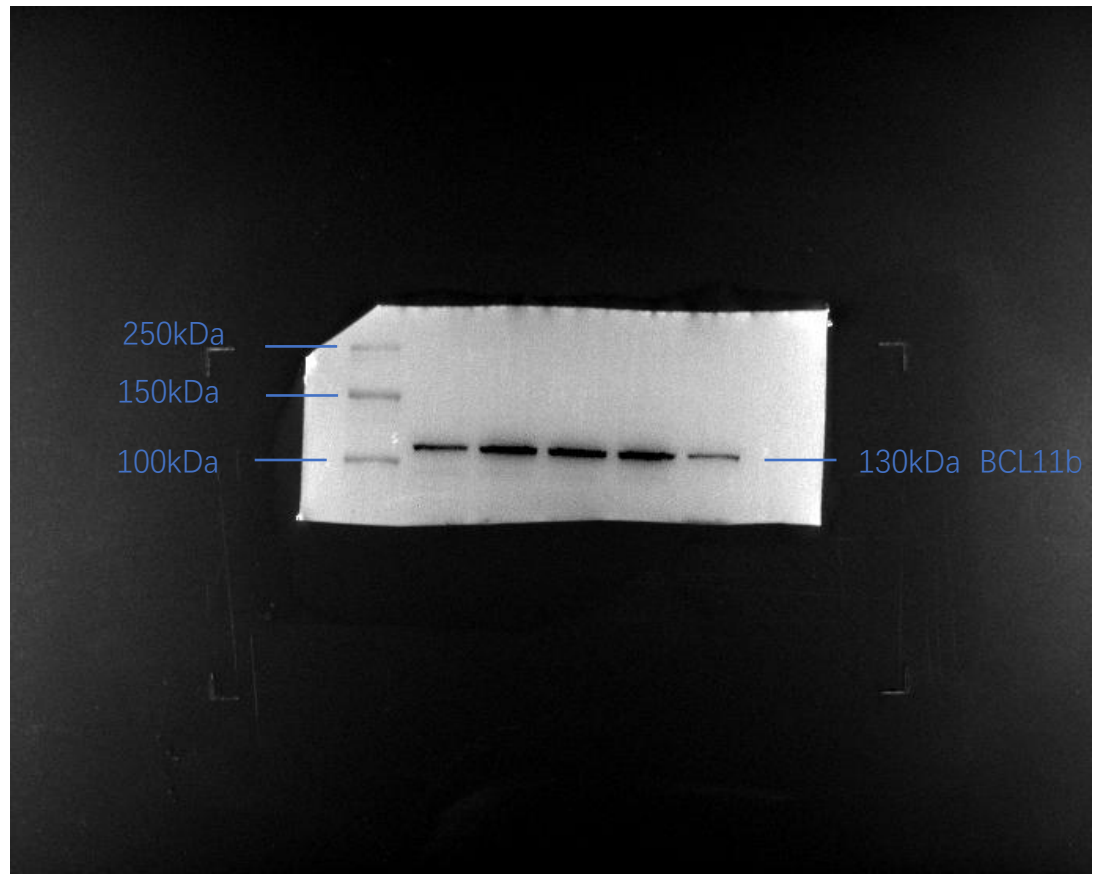

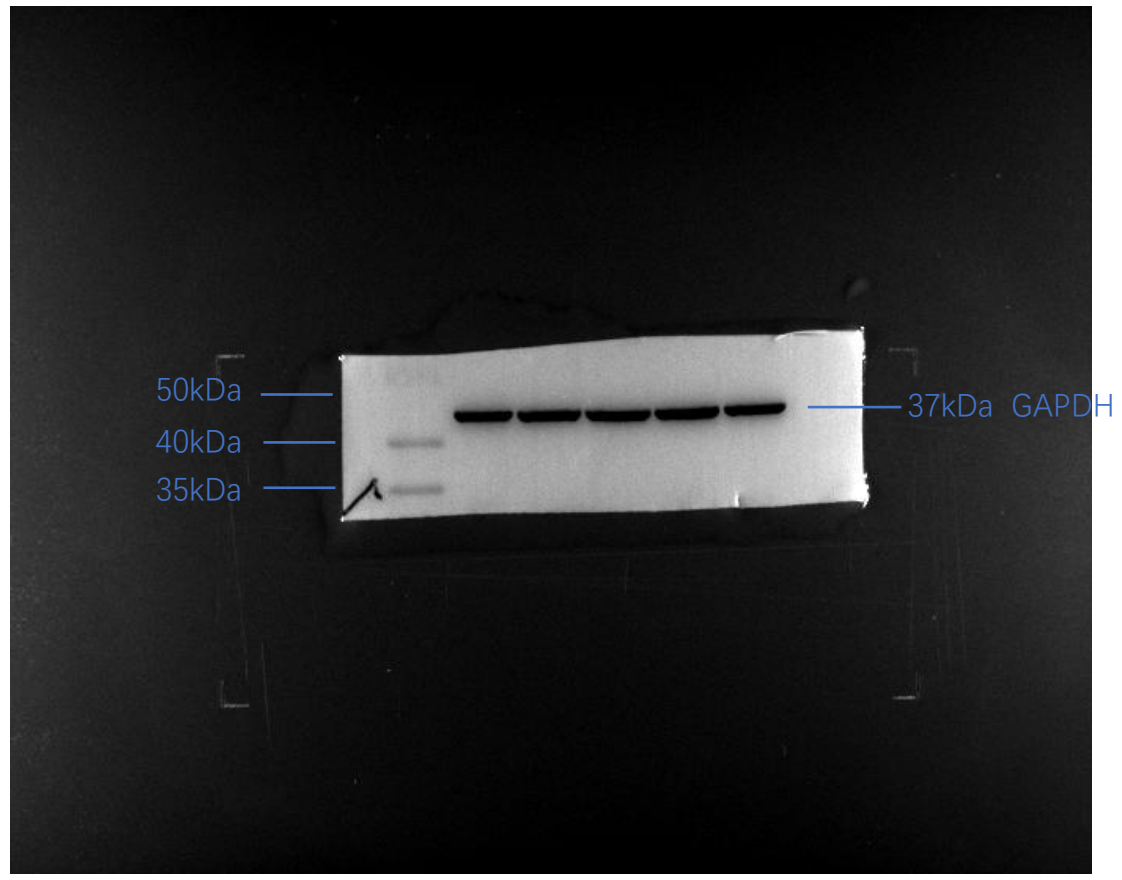

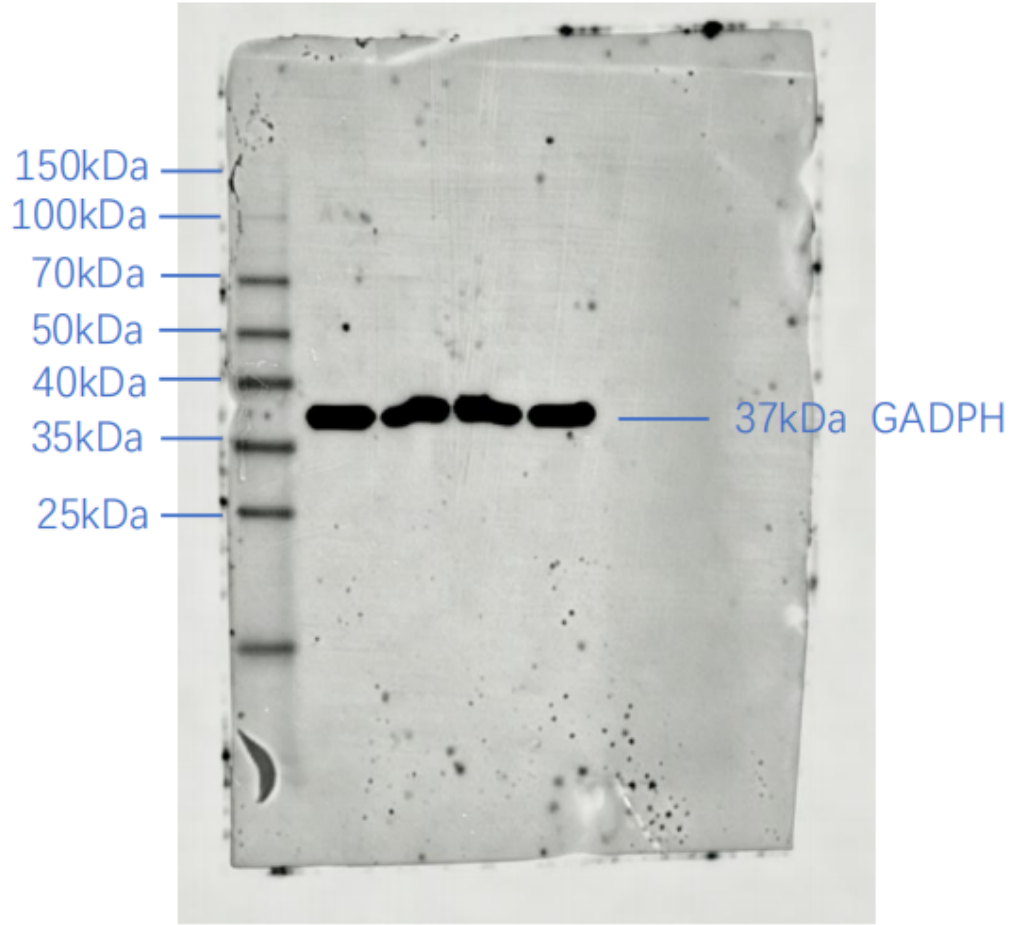

Be showed in the figure

n = 3 independent replicates

Figure3 A

replicates 1

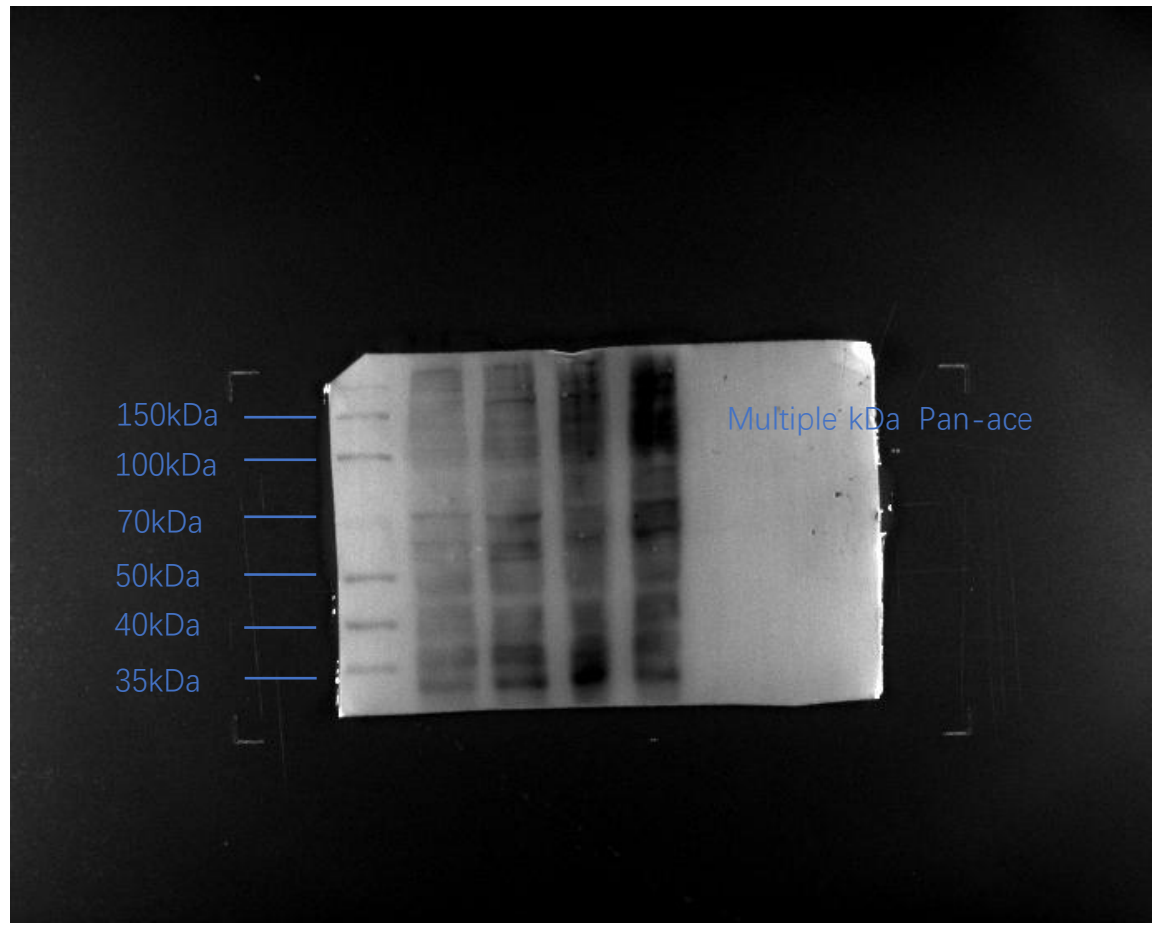

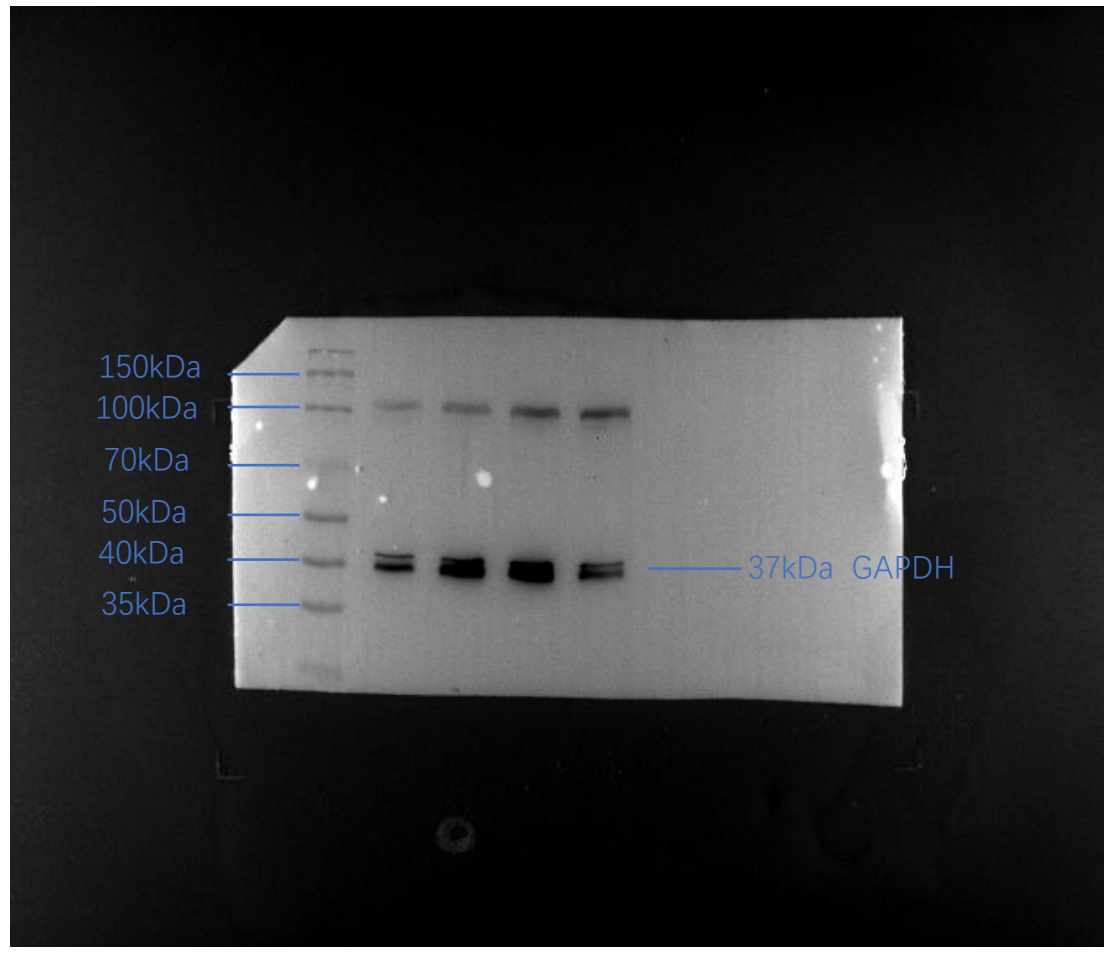

replicates 2

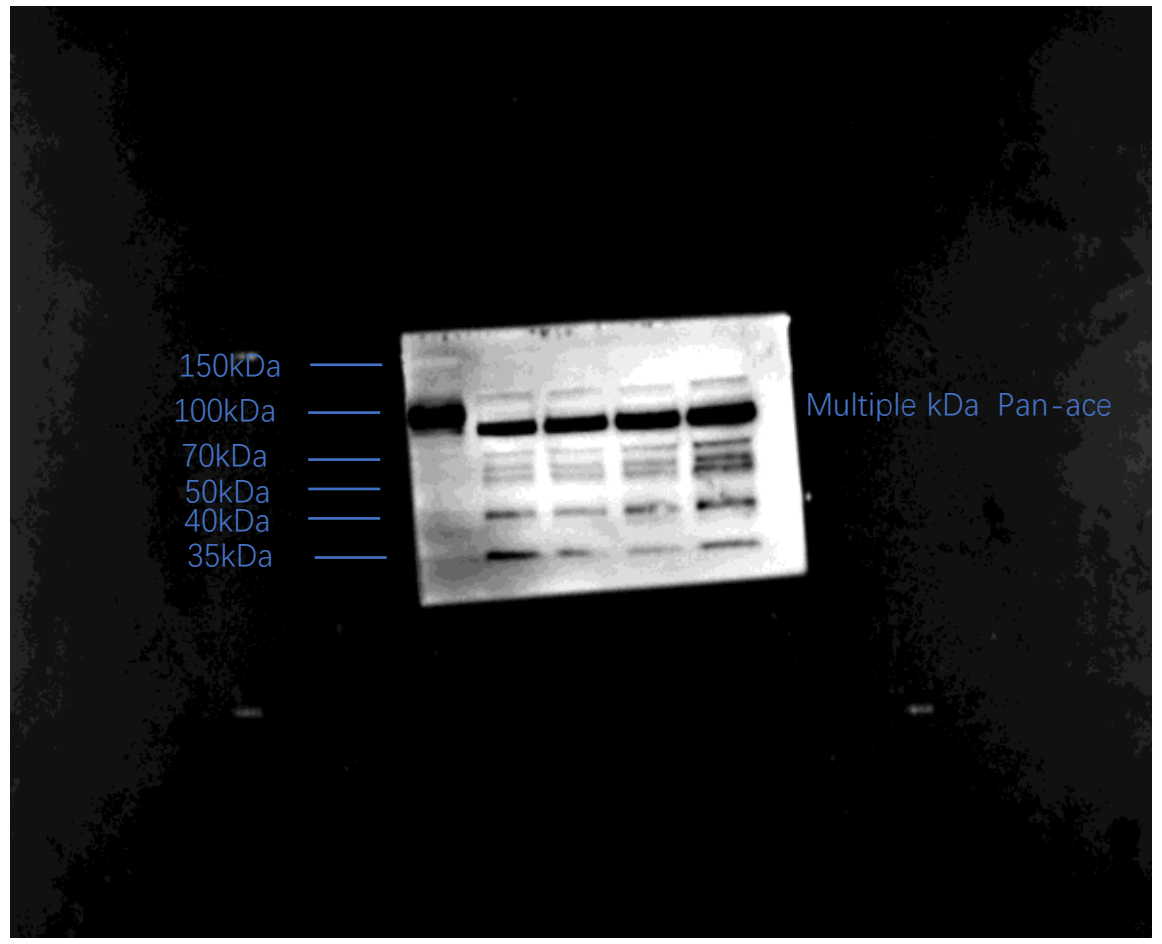

Be showed in the figure

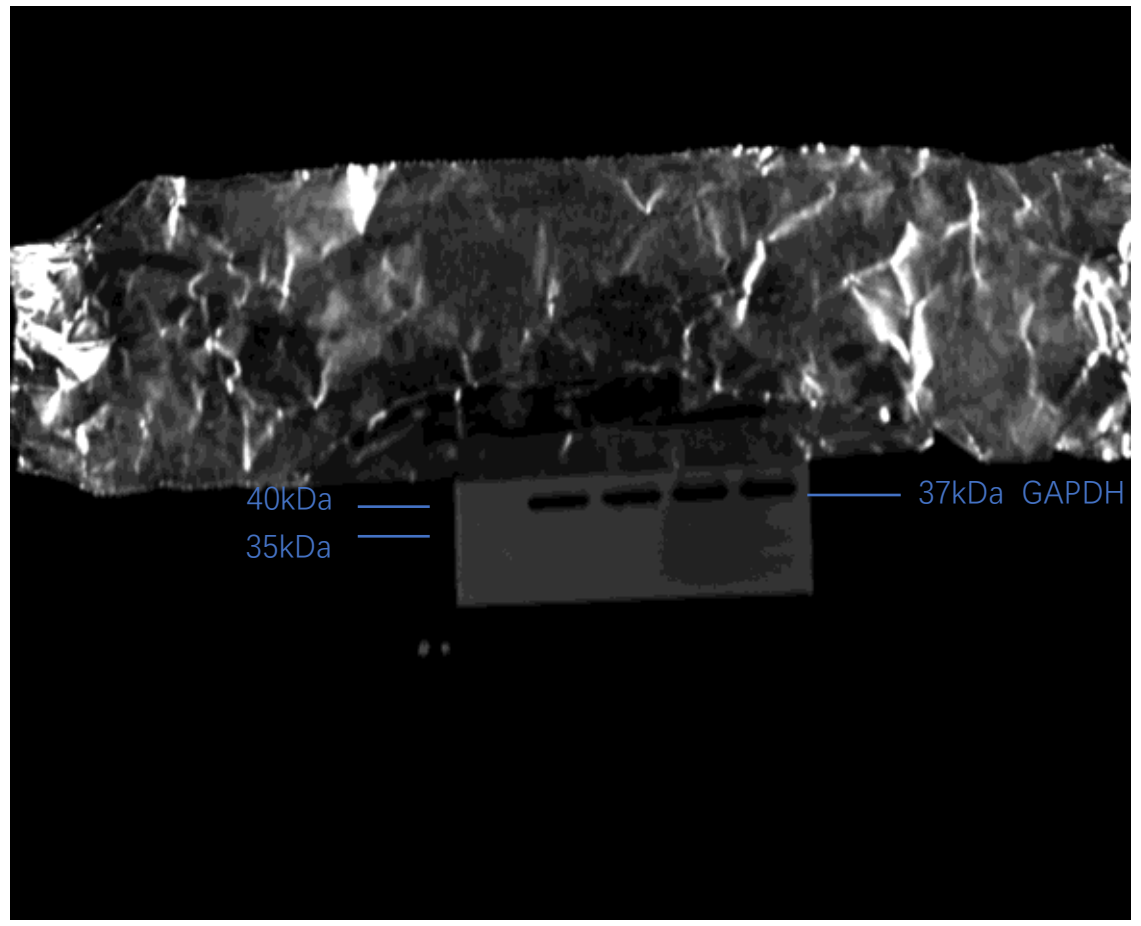

Be showed in the figure

n = 3 independent replicates

Figure3 C

replicates 1

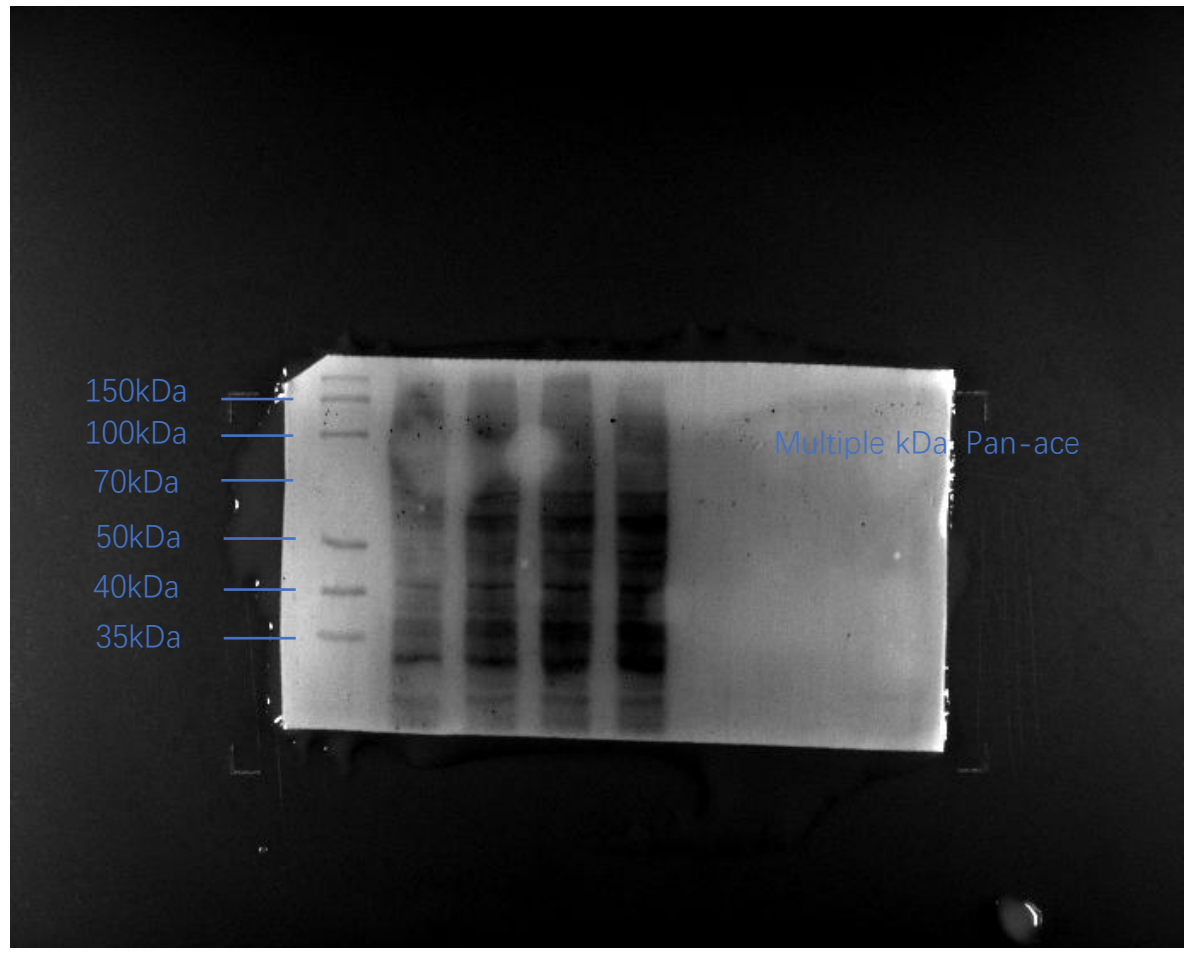

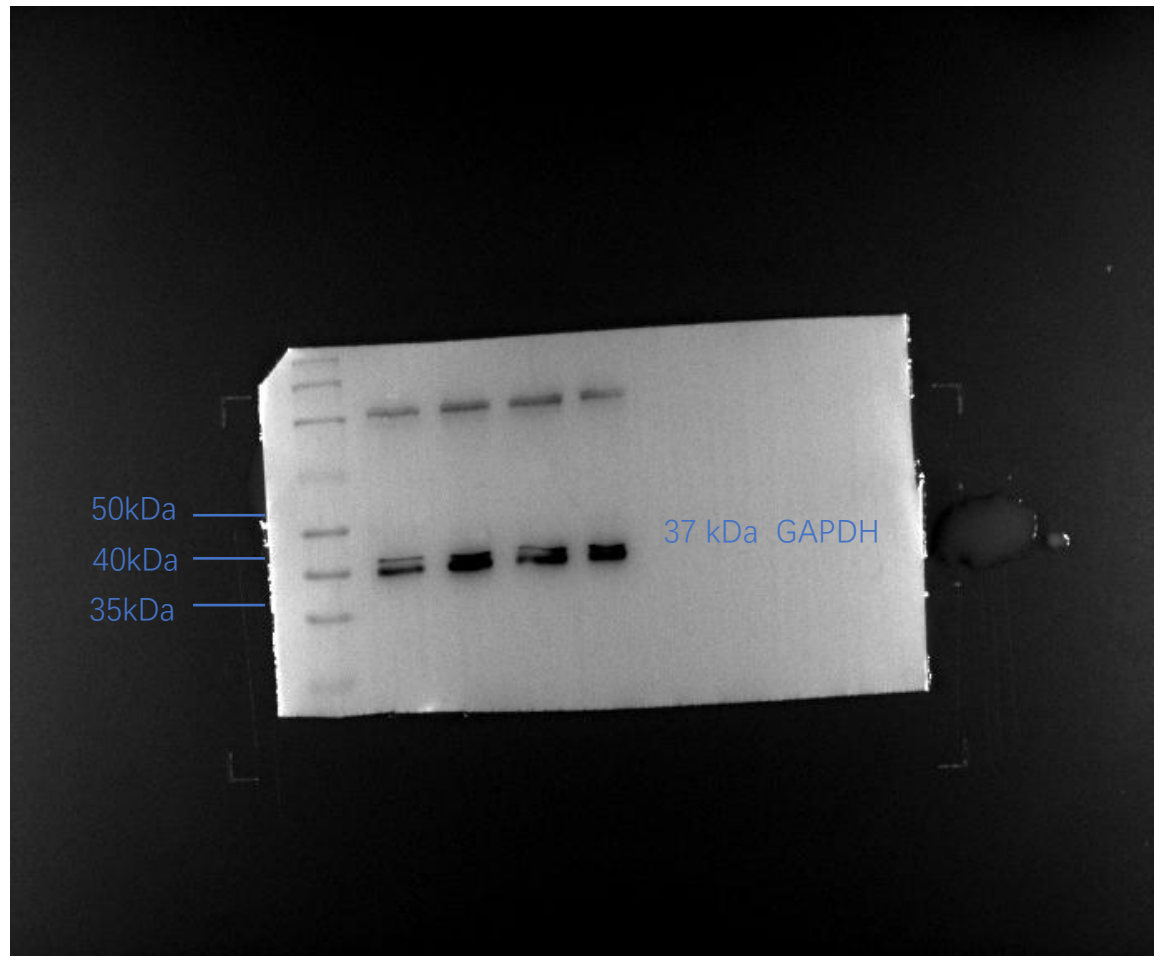

replicates 2

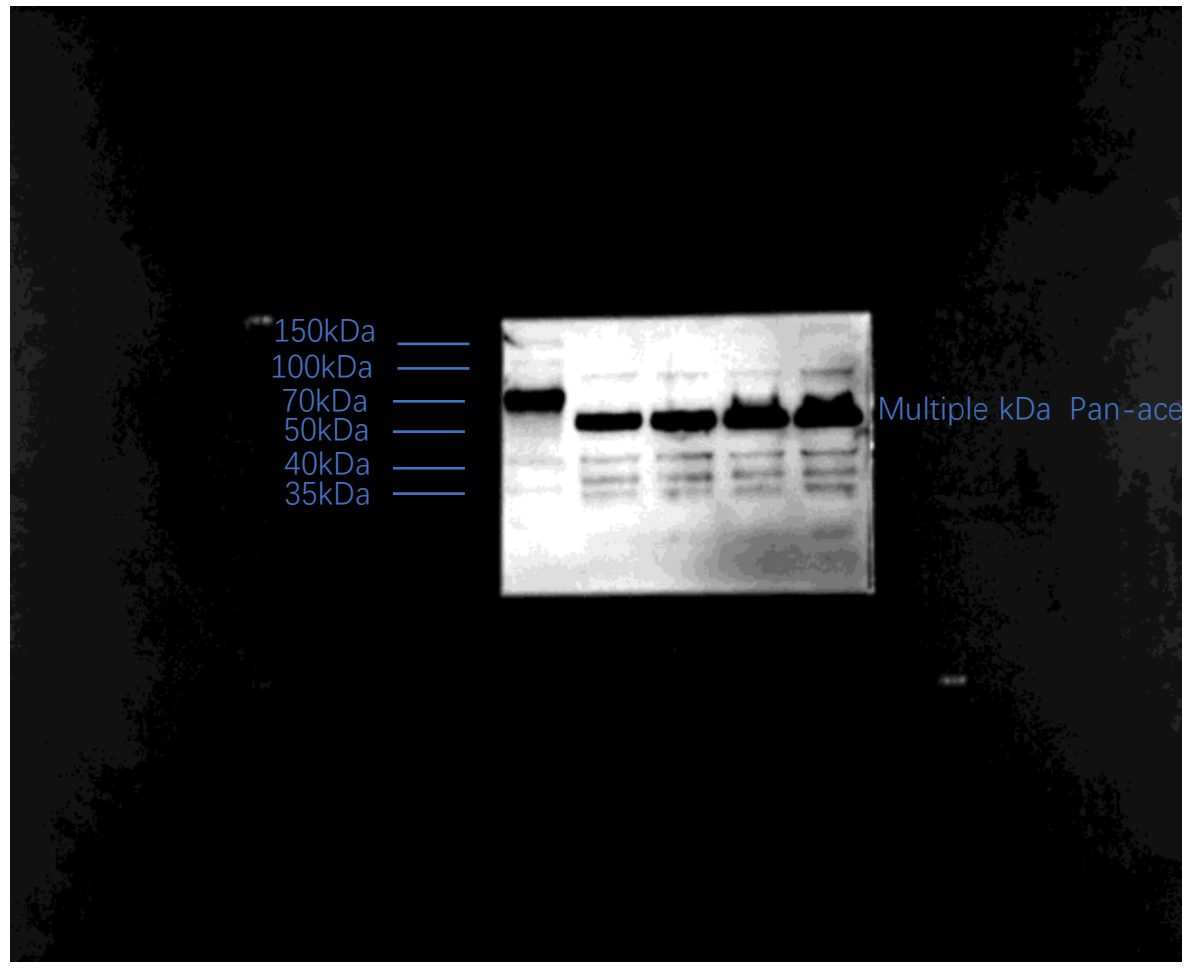

Be showed in the figure

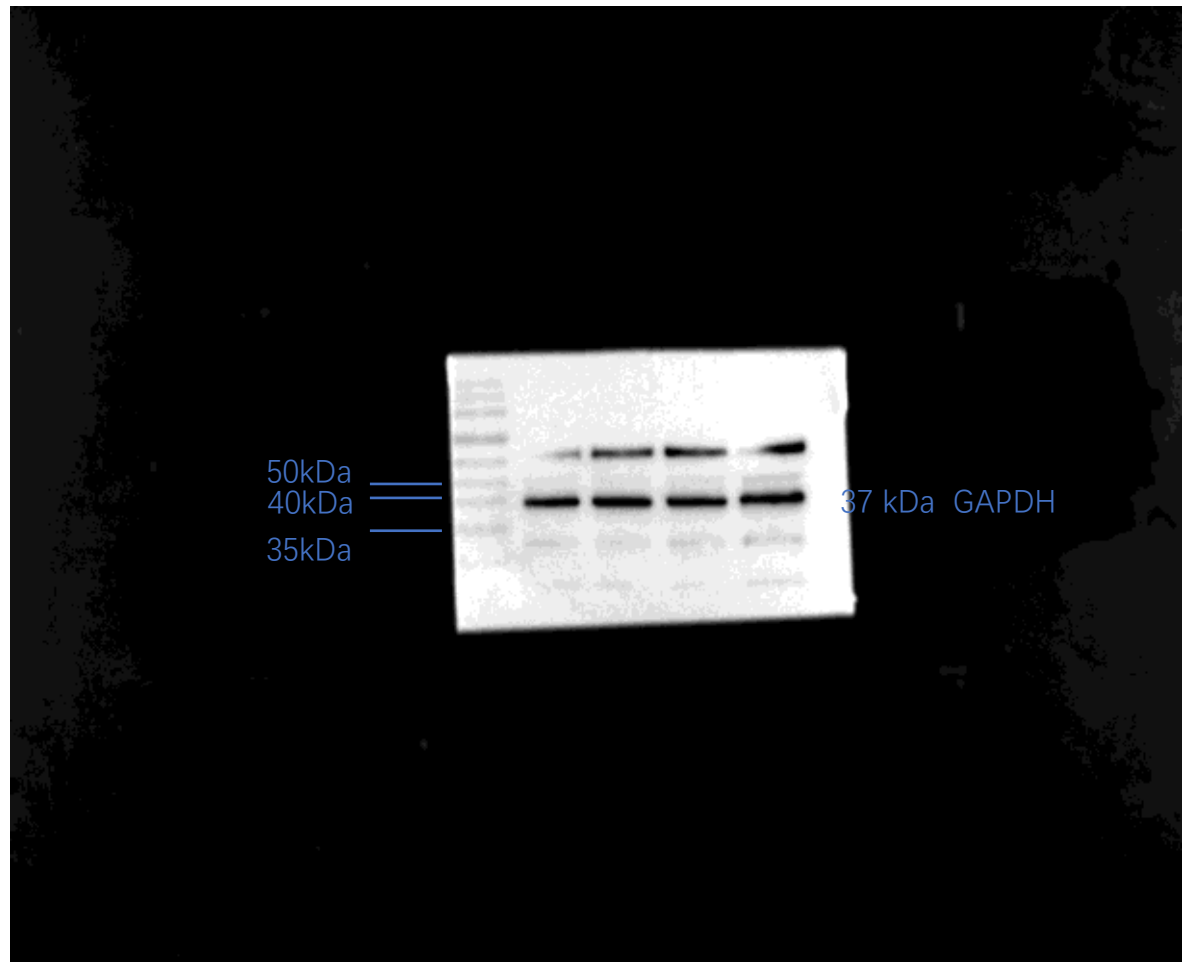

Be showed in the figure

Figure3 F n = 3 independent replicates

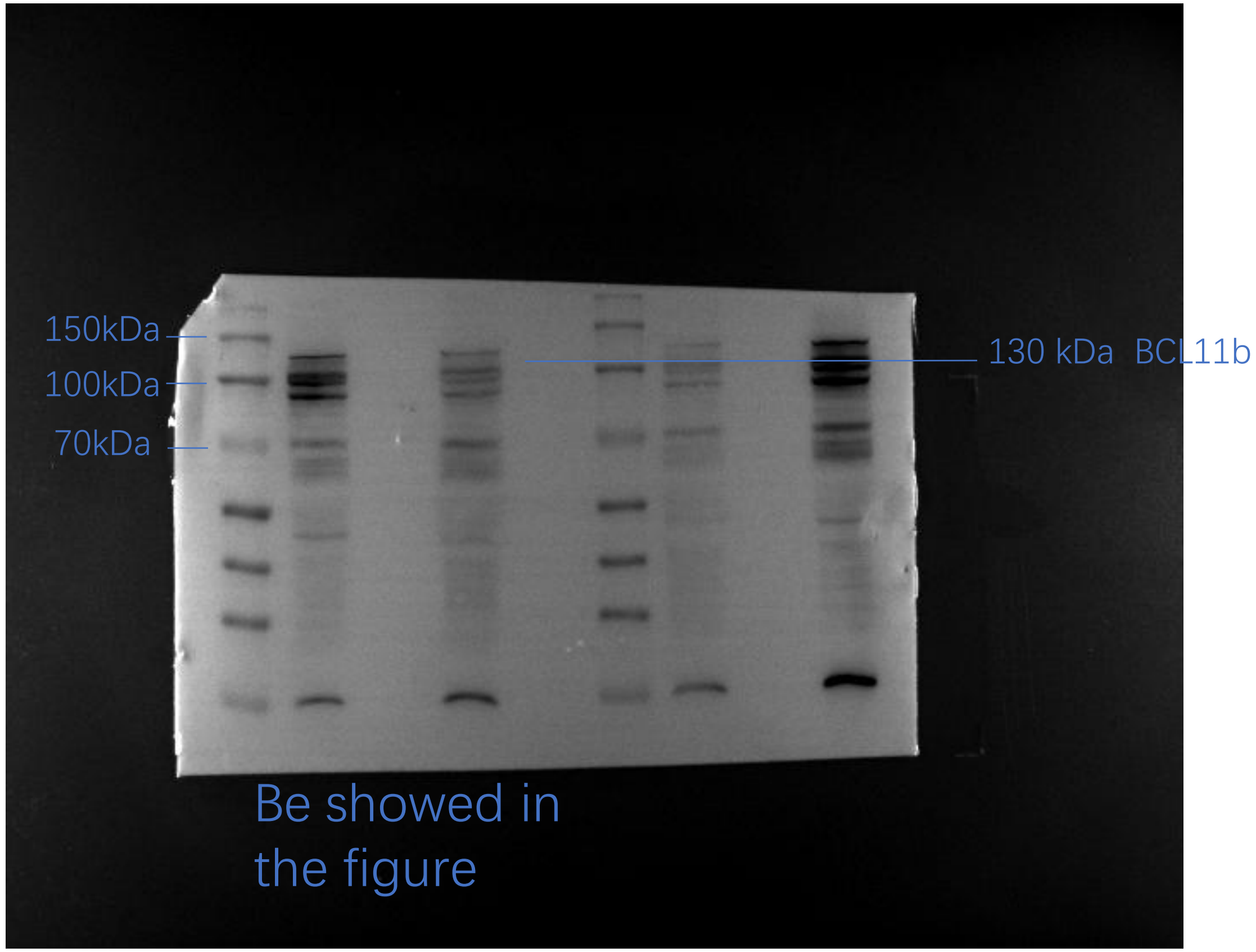

replicates 1 replicates 2

Figure3 G n = 3 independent replicates

replicates 1

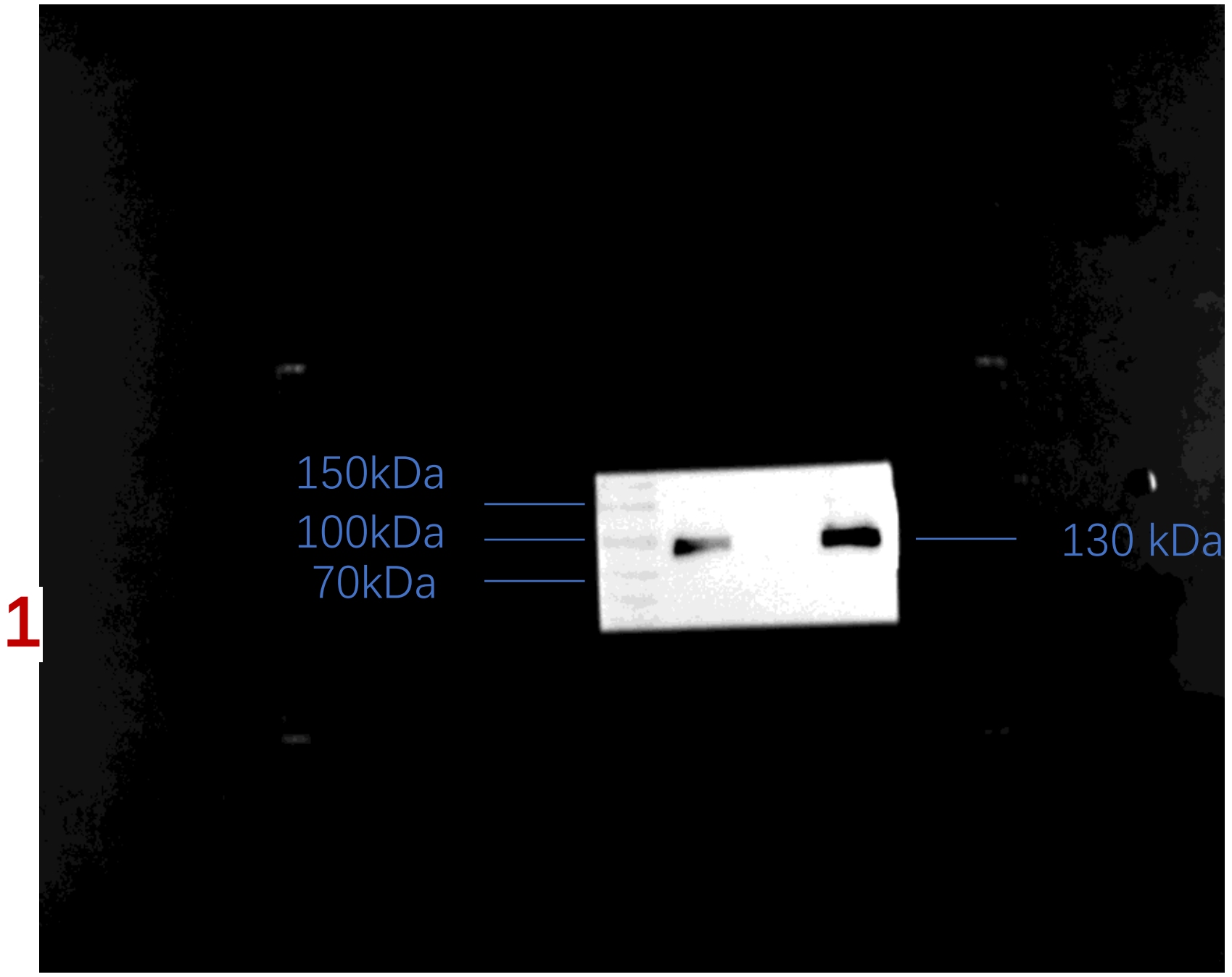

Be showed in the figure

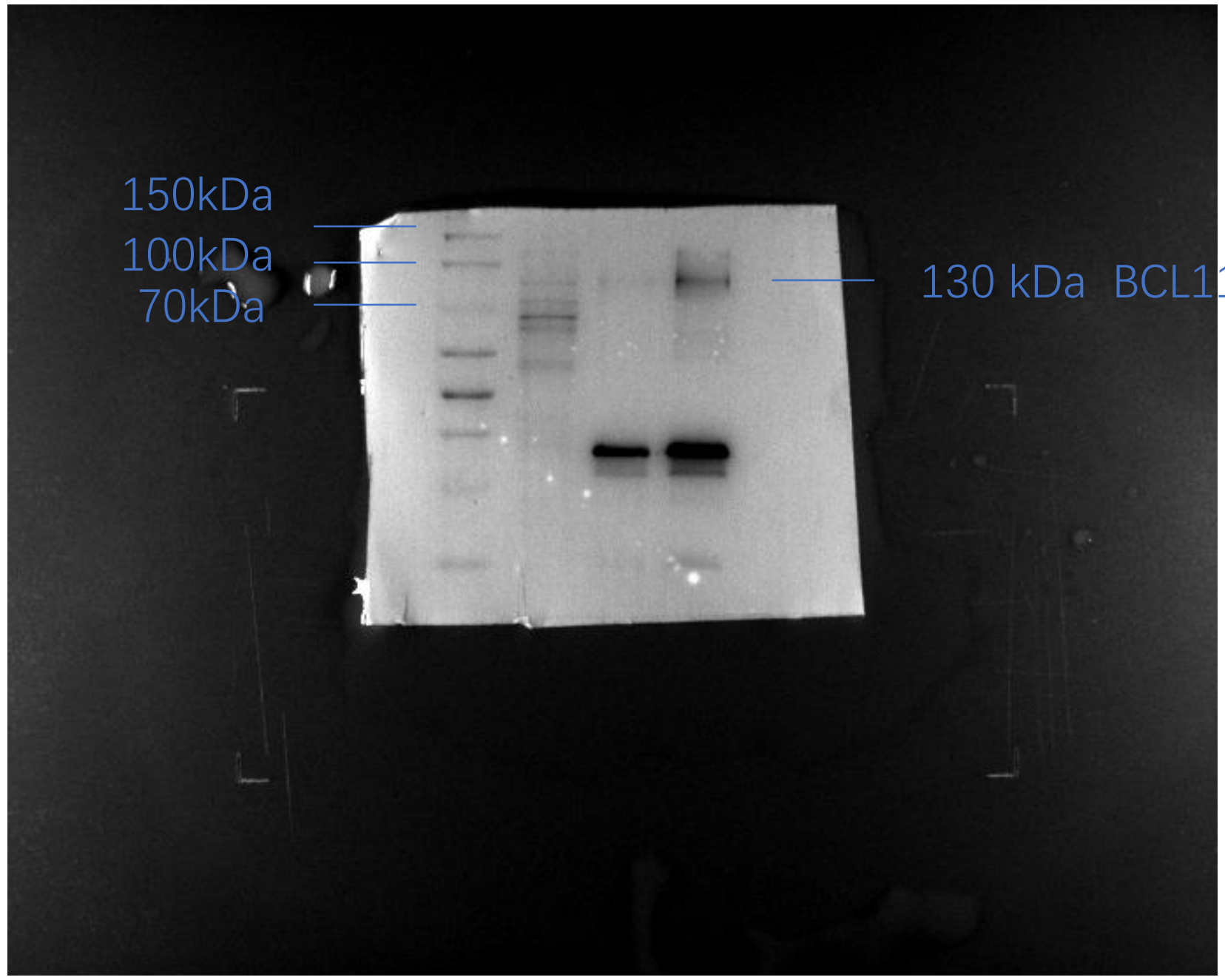

replicates 2

Figure3 G n = 3 independent replicates

replicates 1

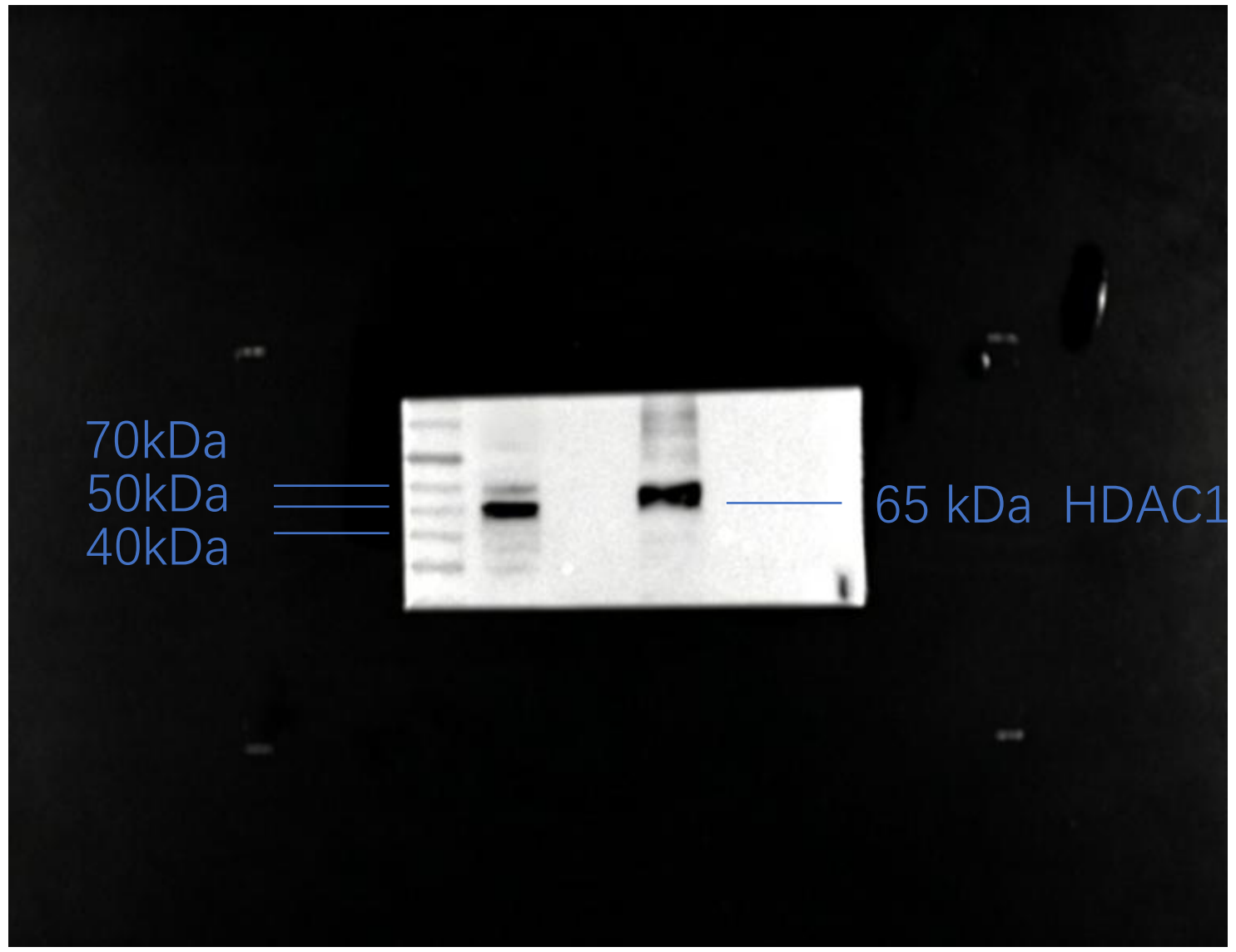

Be showed in the figure

replicates 2

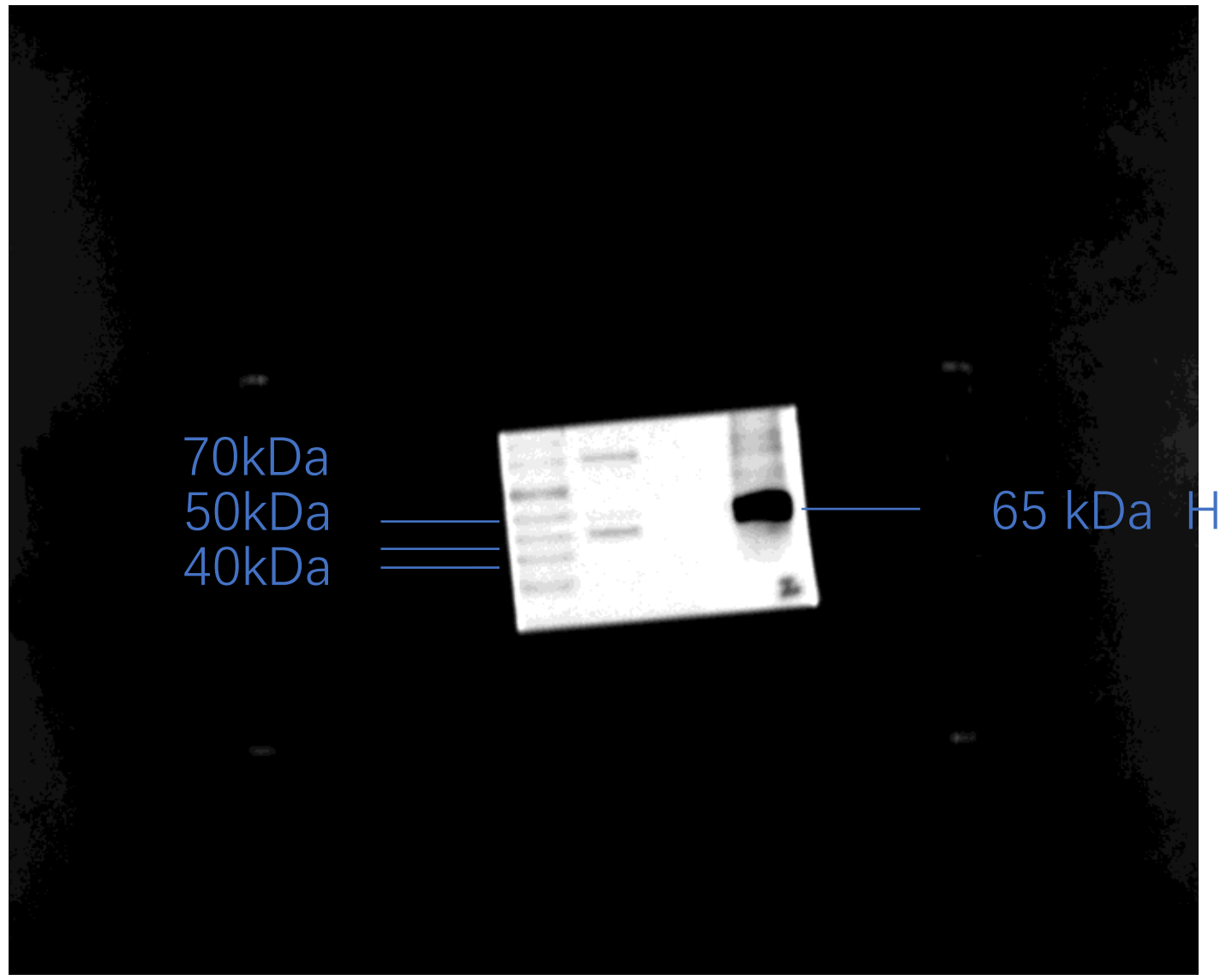

Figure3 G n = 3 independent replicates

replicates 1

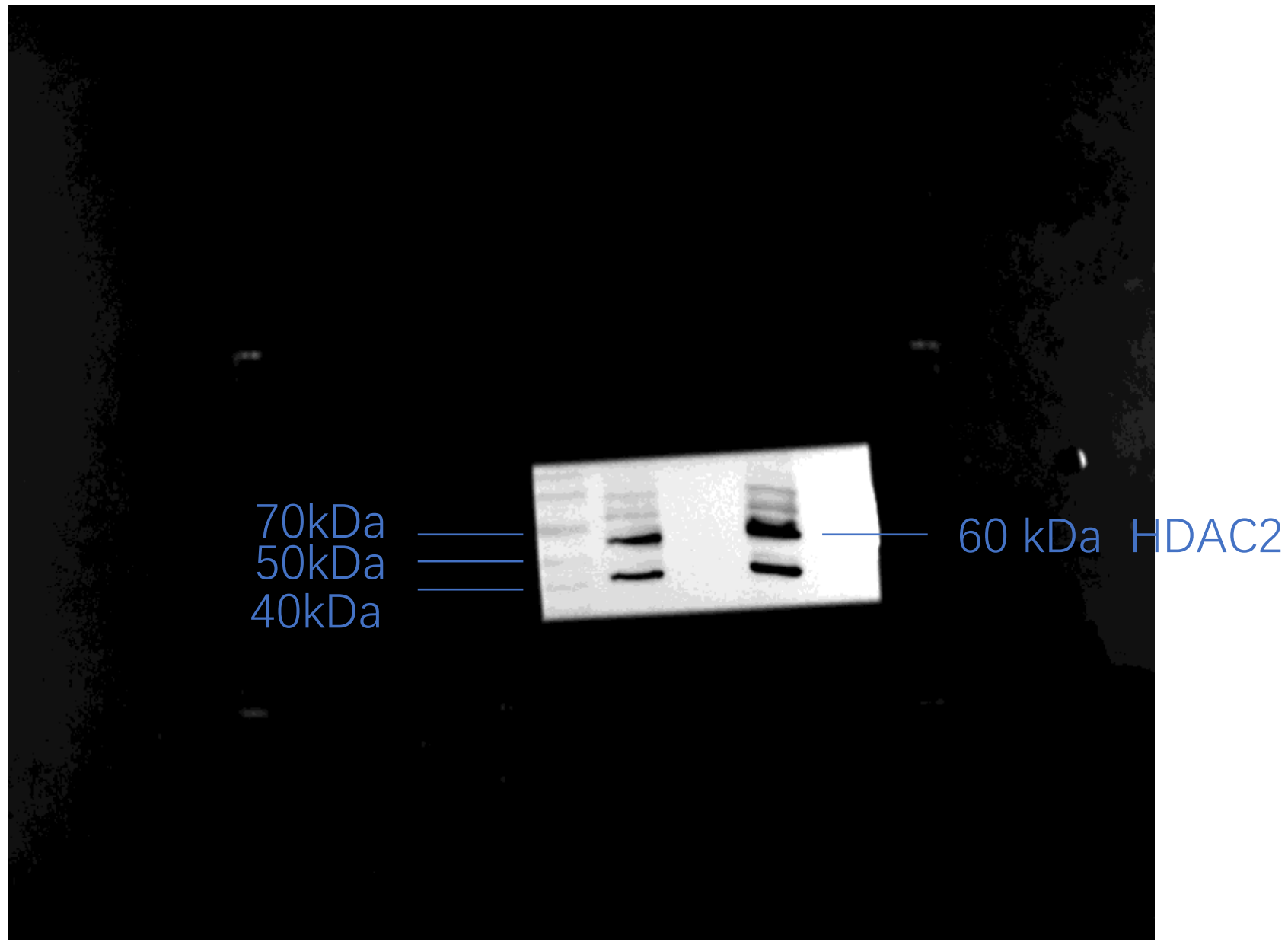

Be showed in the figure

replicates 2

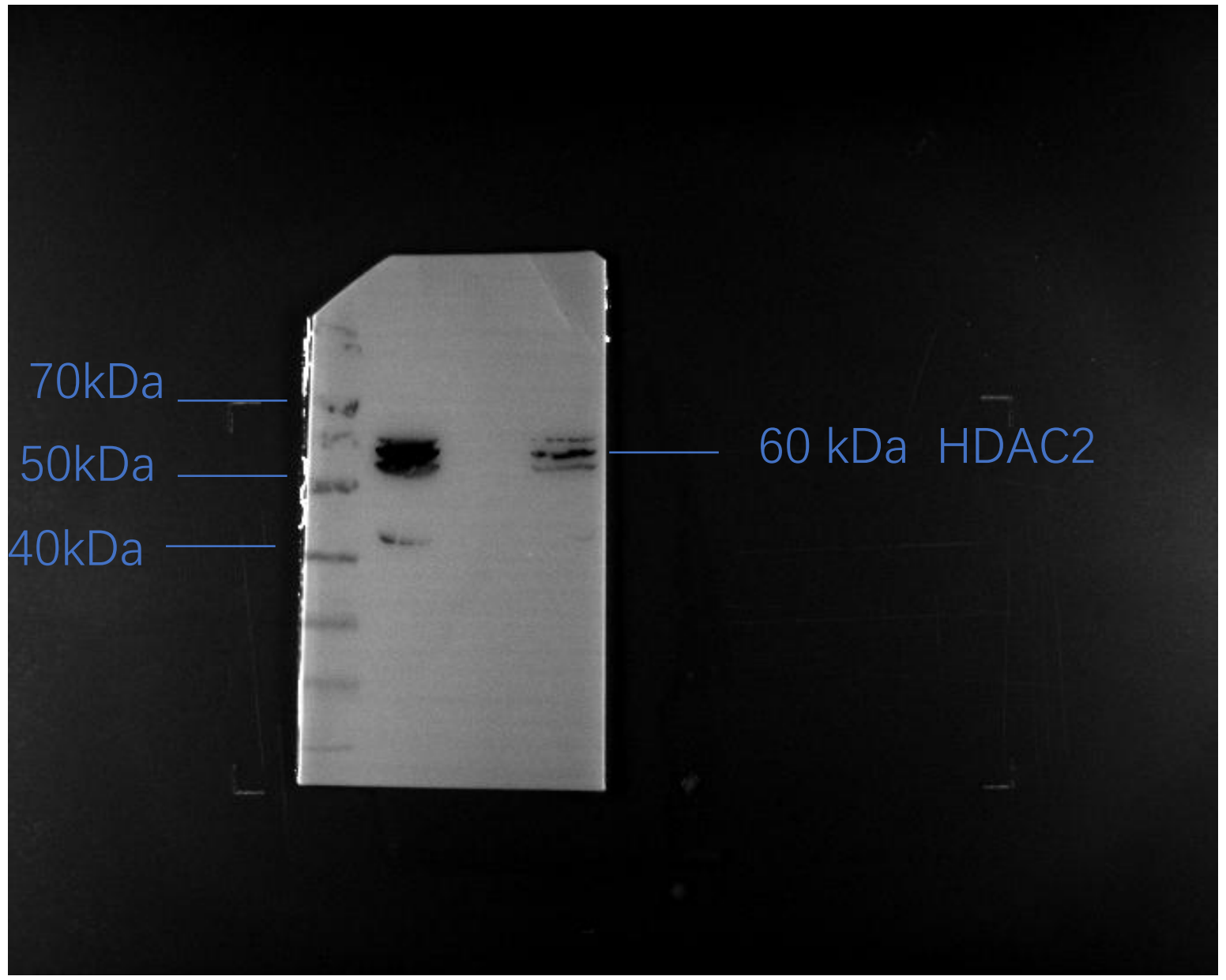

Figure3 G n = 3 independent replicates

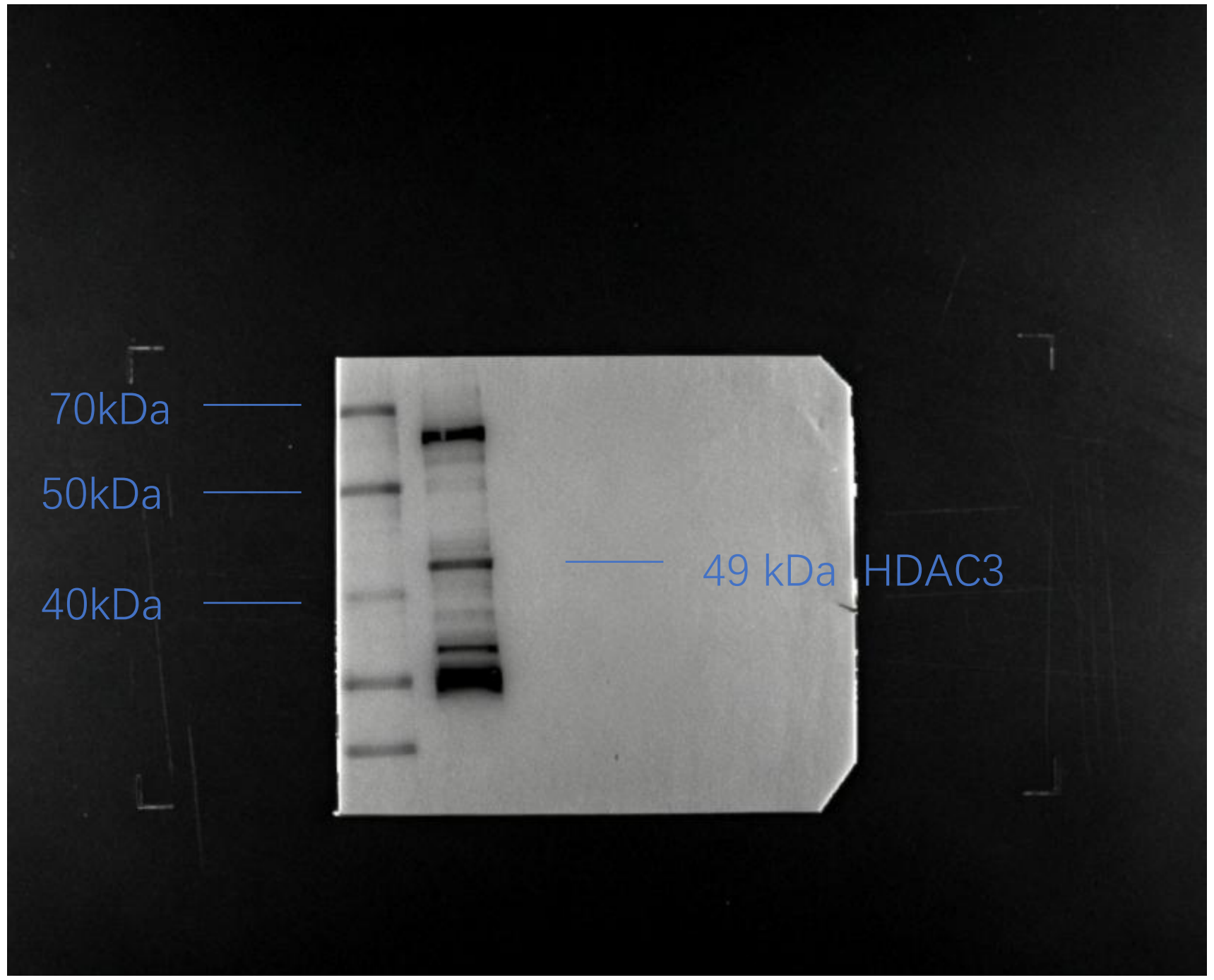

replicates 1

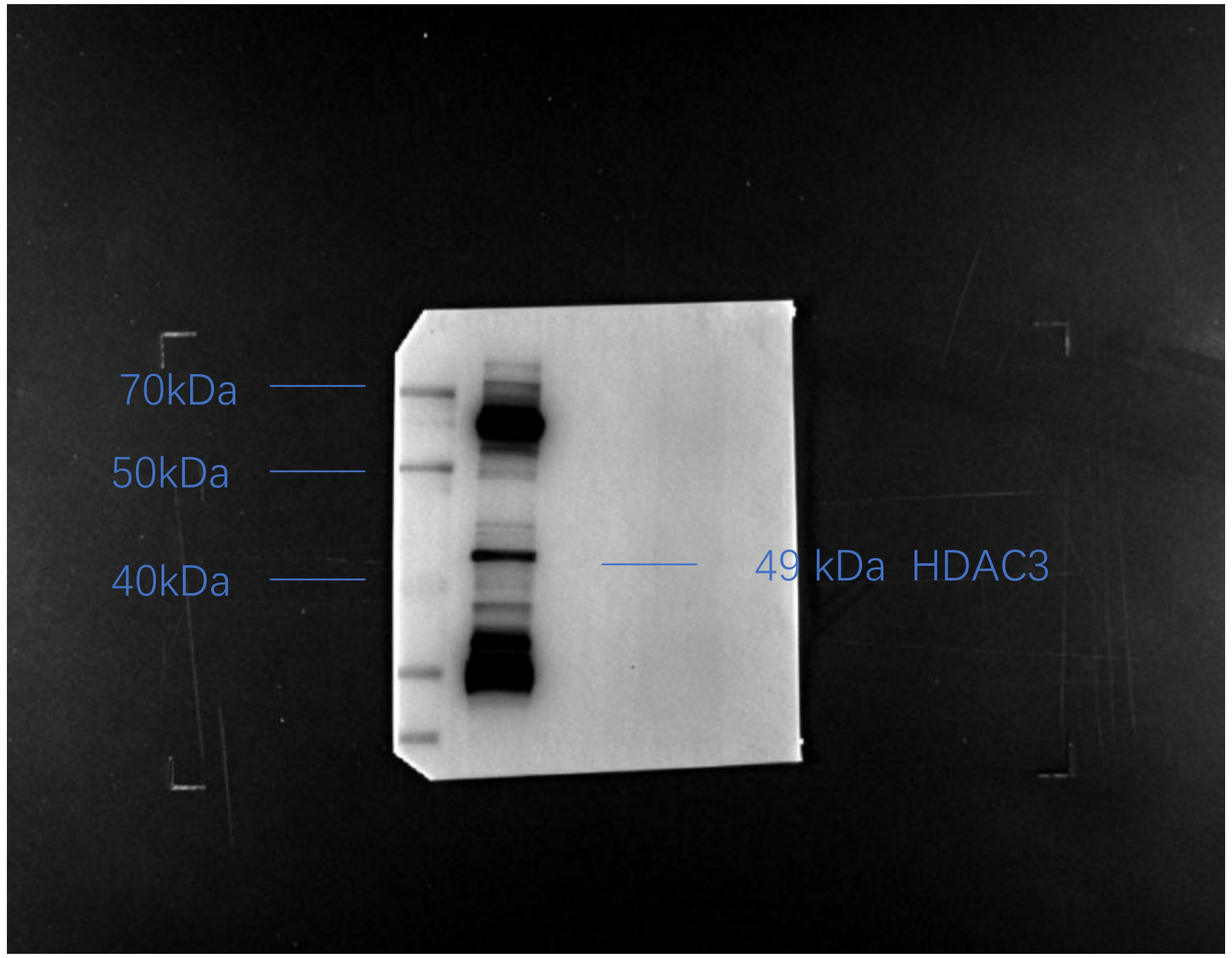

Be showed in the figure

replicates 2

Figure4 D n = 3 independent replicates

replicates 1

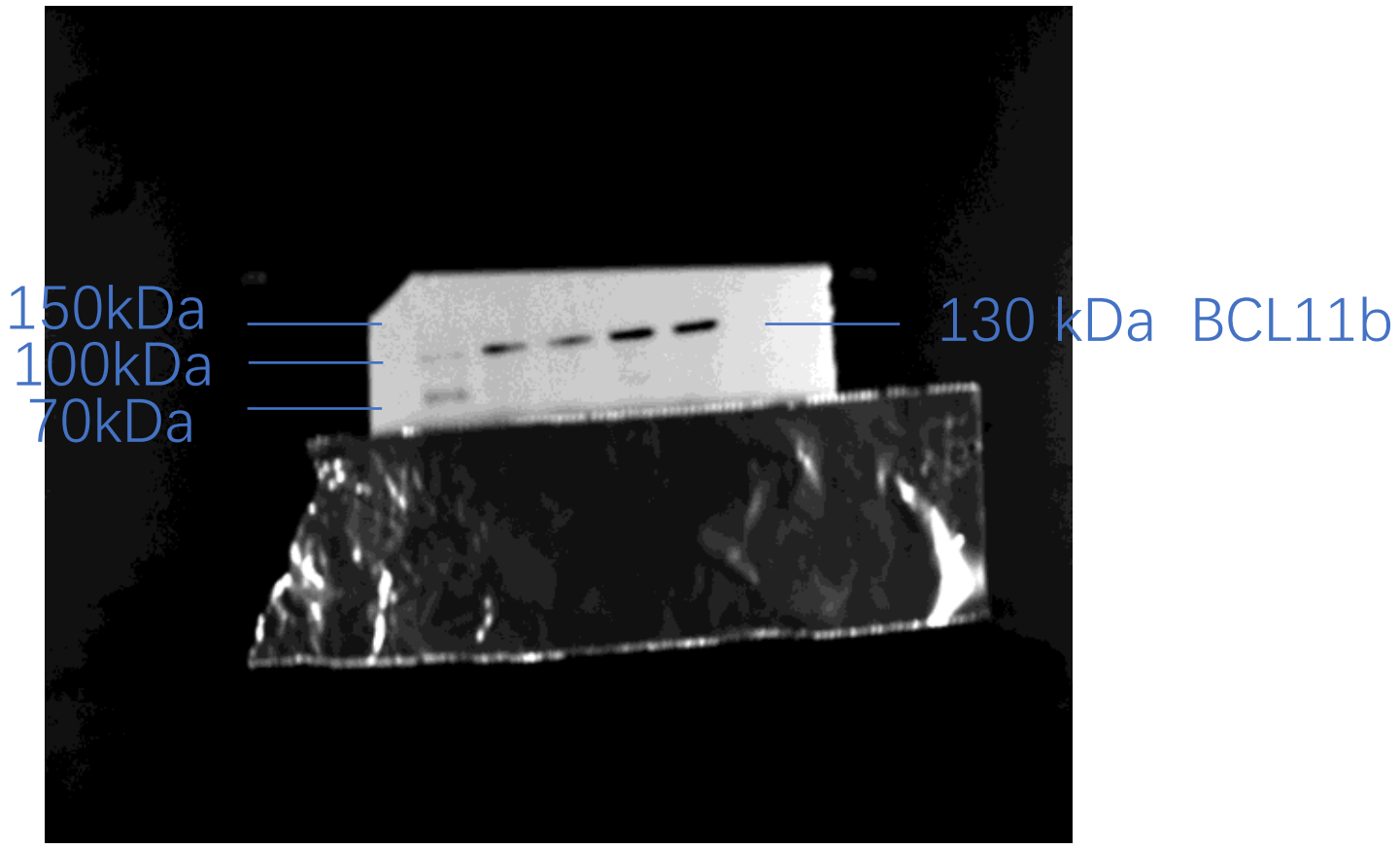

Be showed in the figure

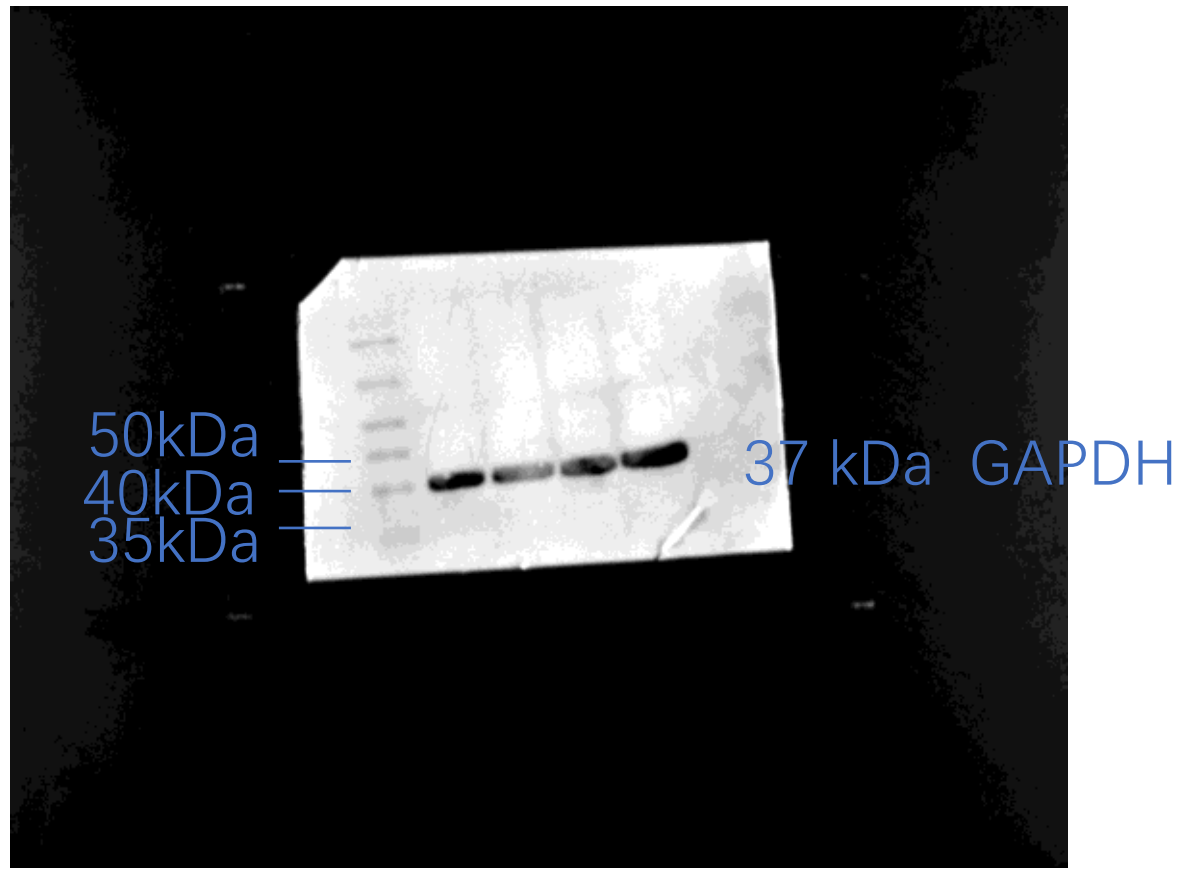

Be showed in the figure

replicates 2

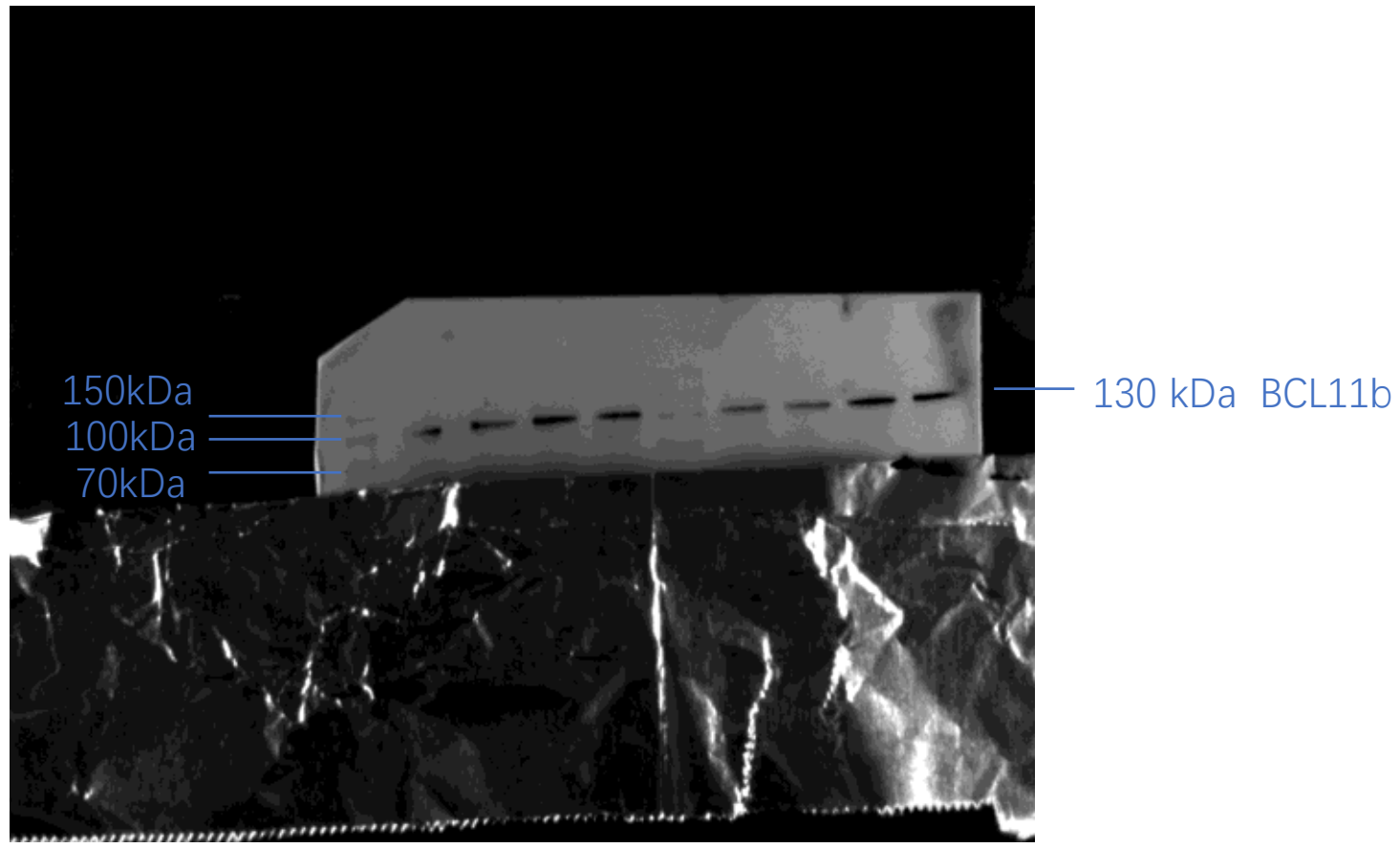

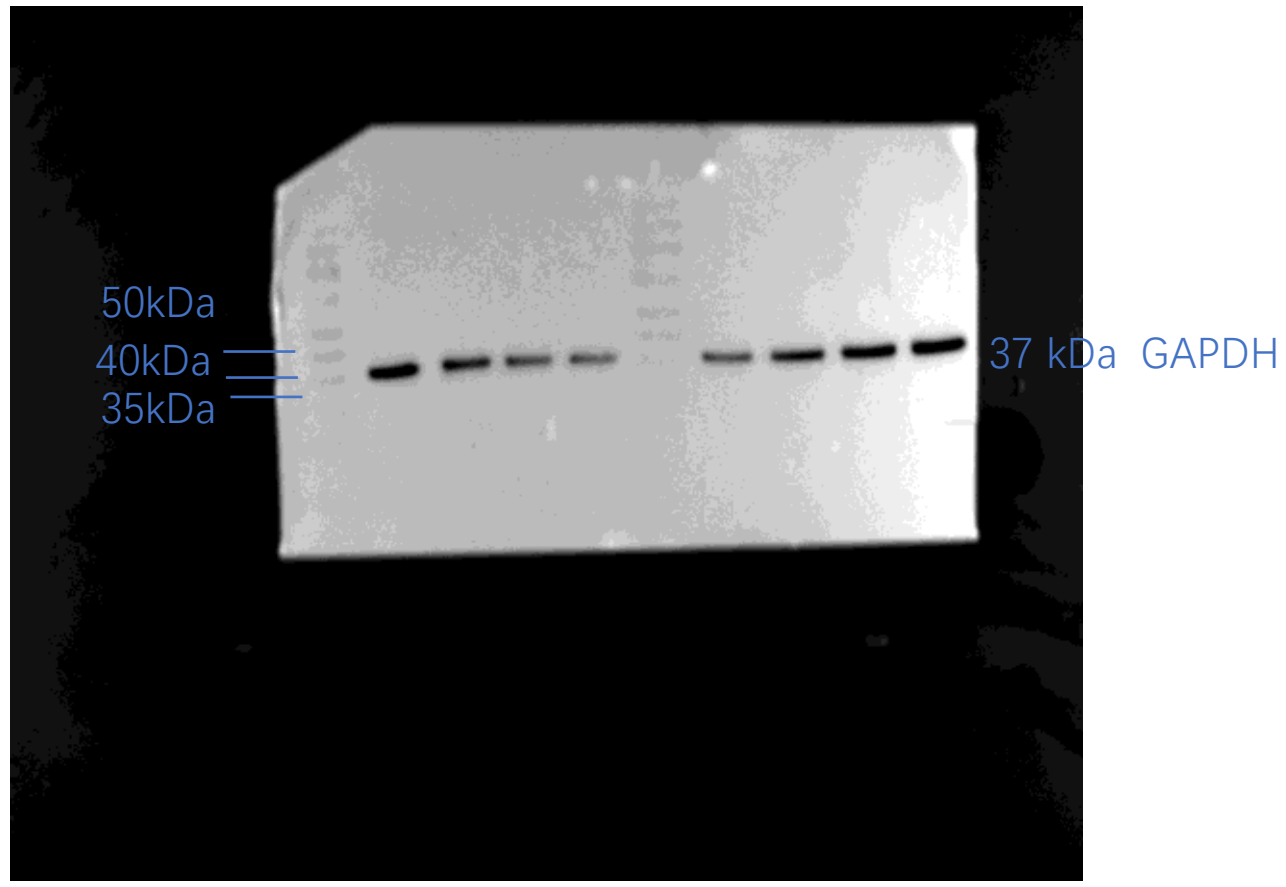

Figure5 C n = 3 independent replicates

replicates 1 replicates 2 replicates 1 replicates 2

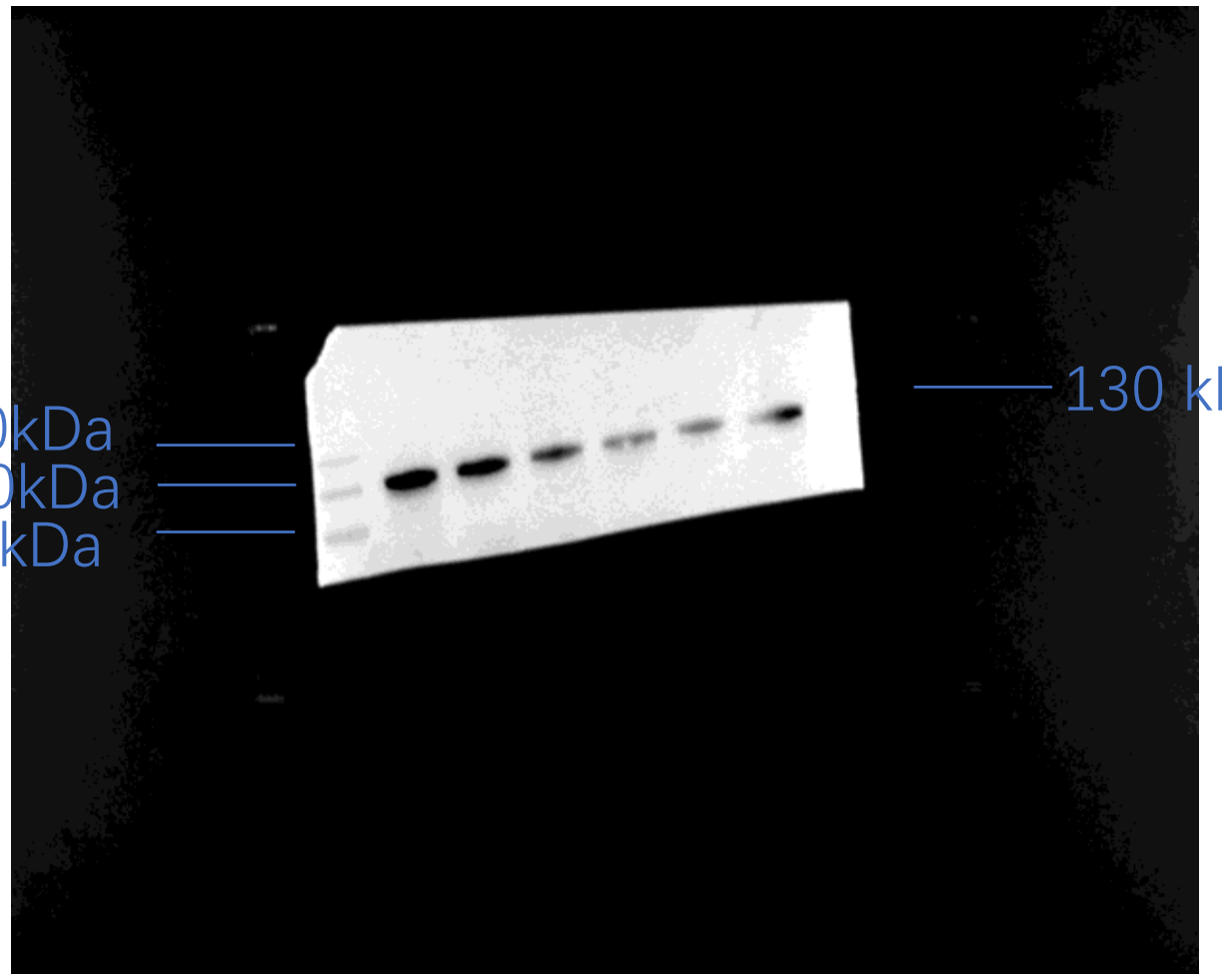

Be showed in the figure

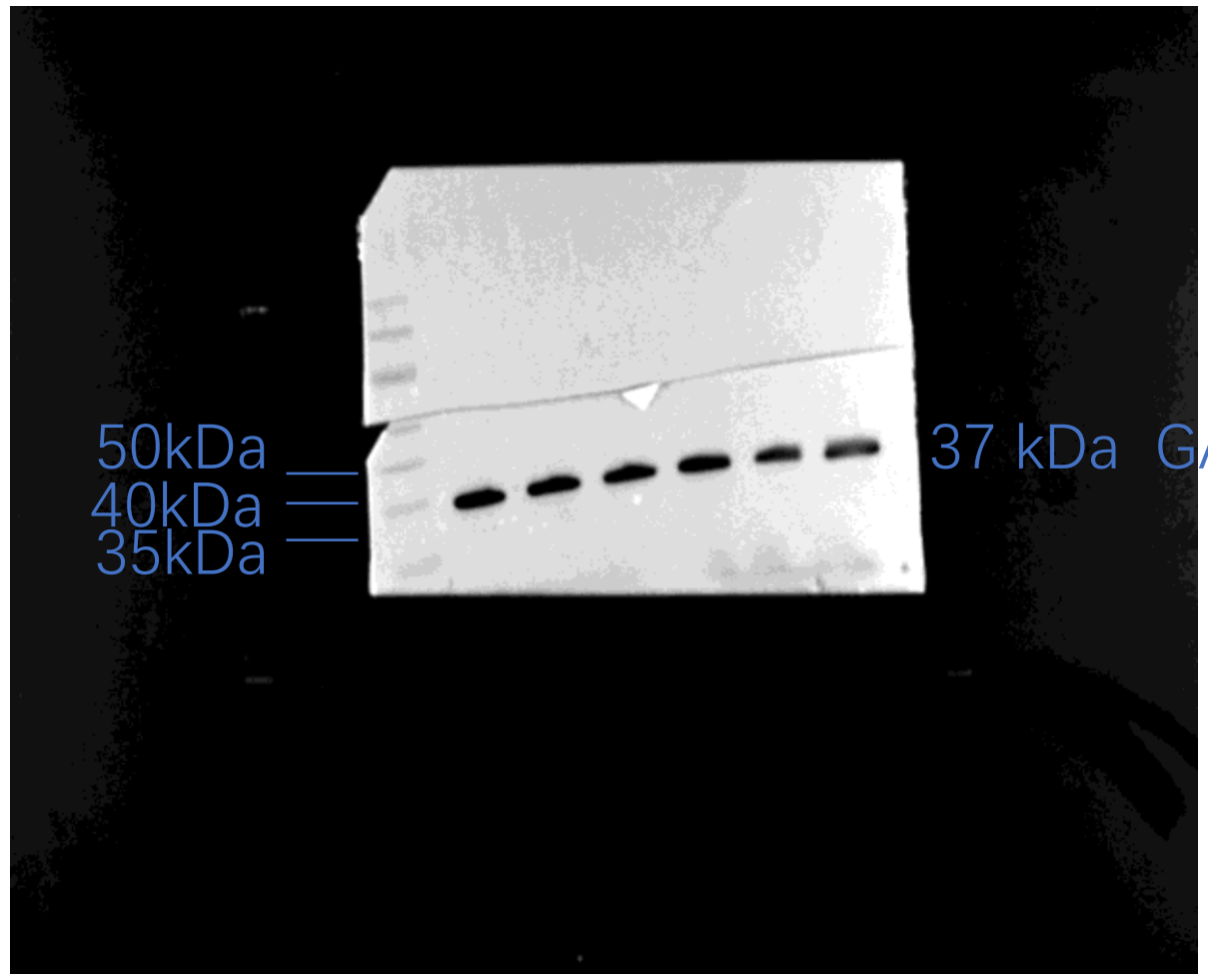

Be showed in the figure

Figure 5 I

n = 3 independent replicates

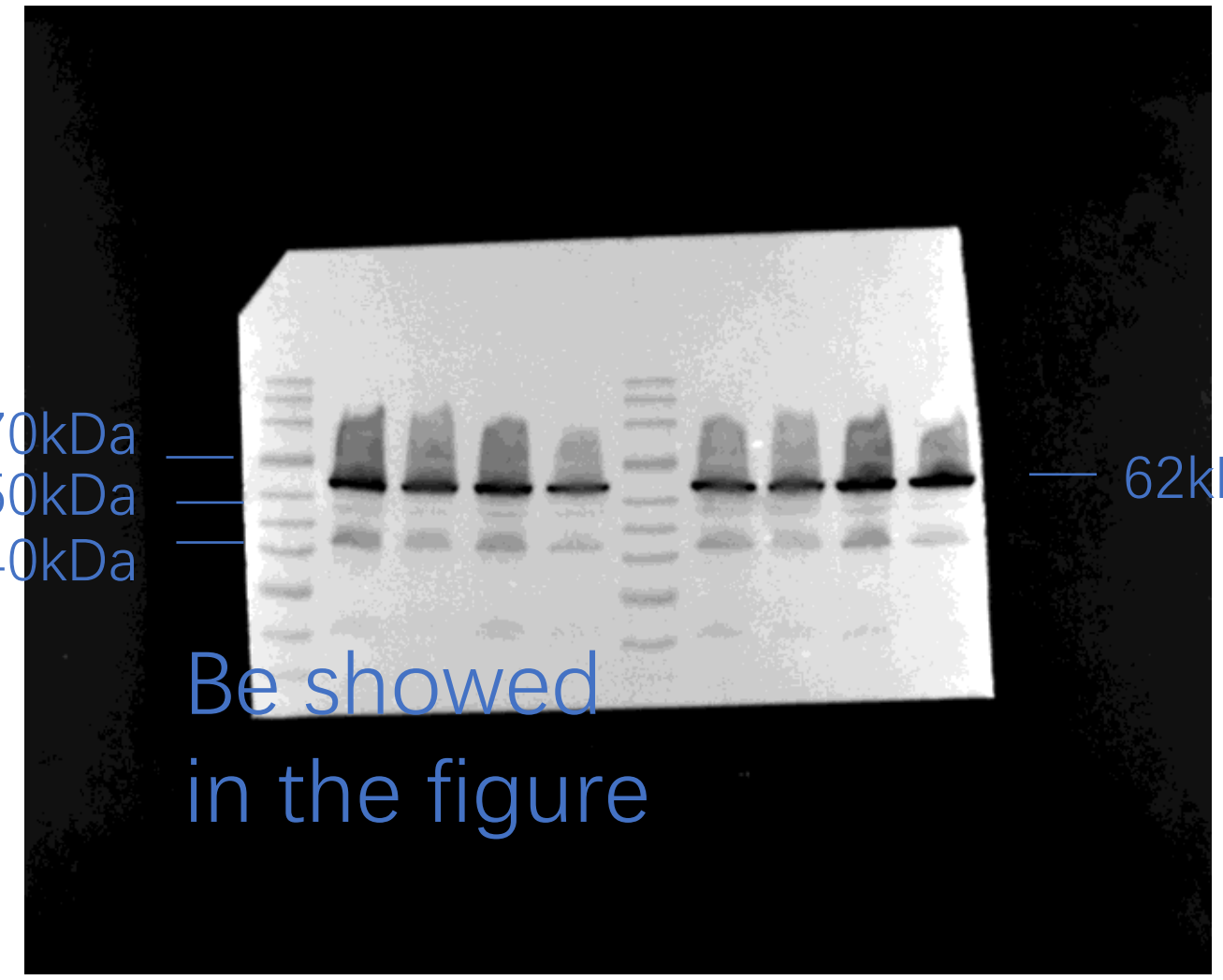

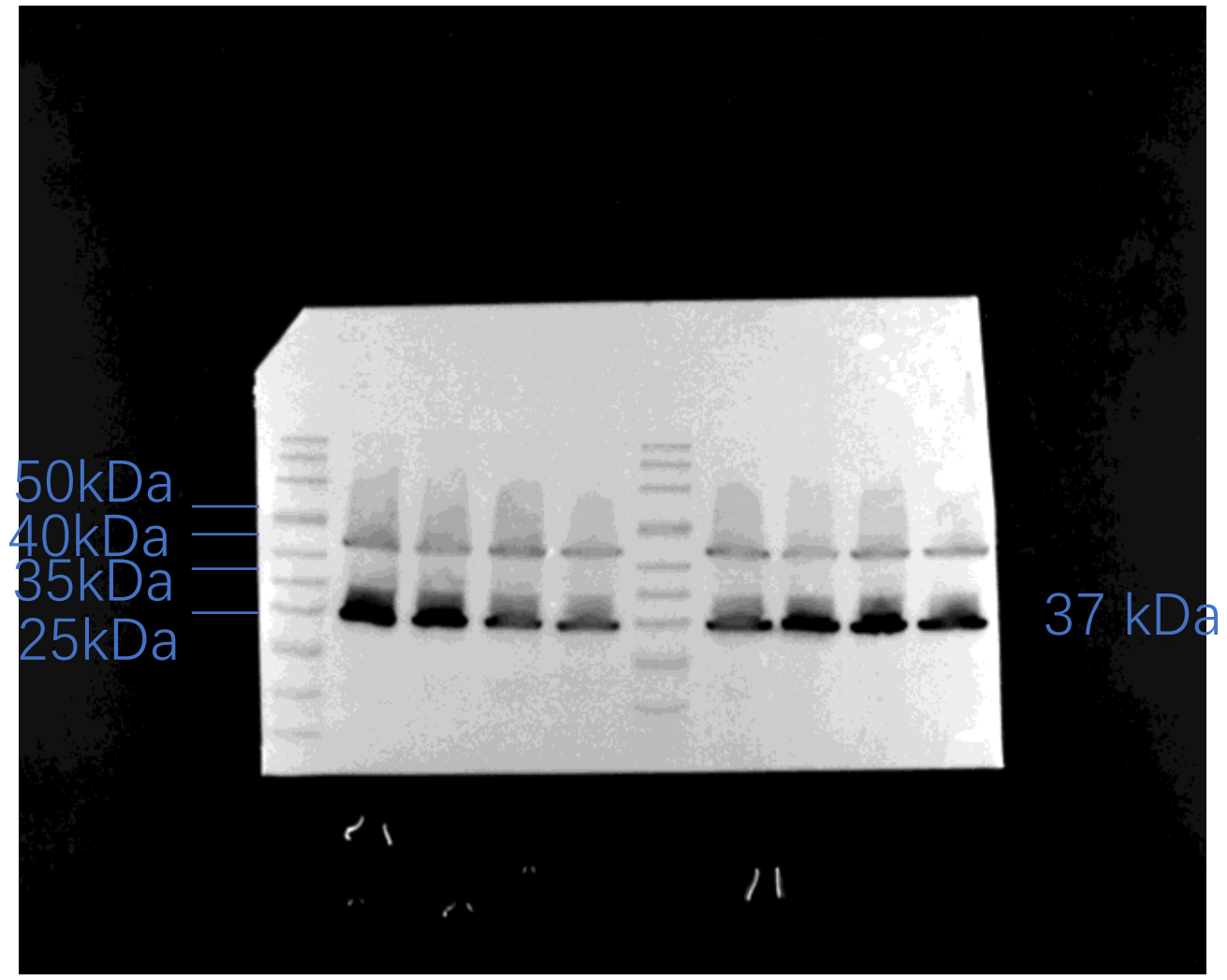

Figure5 I n = 3 independent replicates

replicates 1 replicates 2

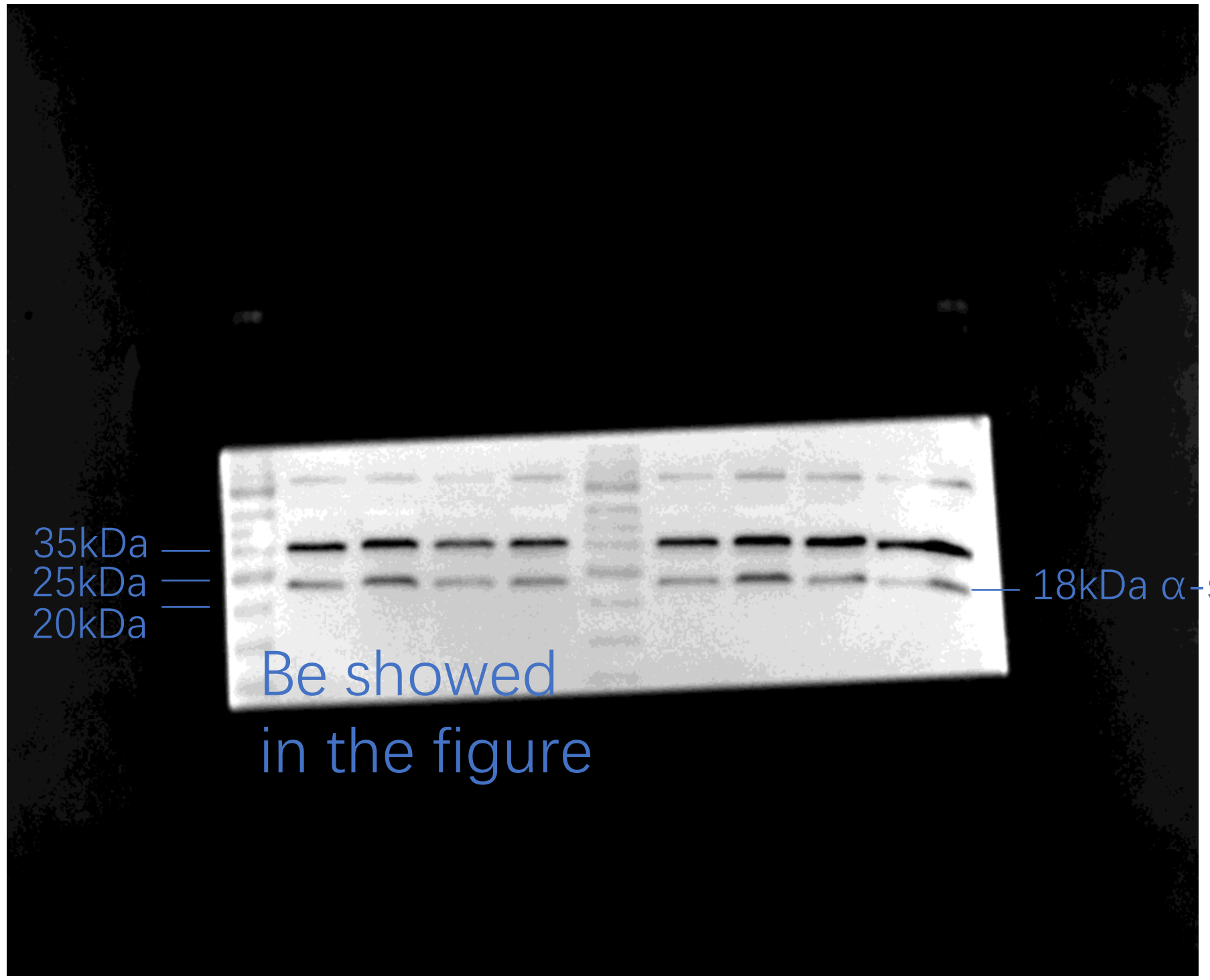

replicates 1 replicates 2

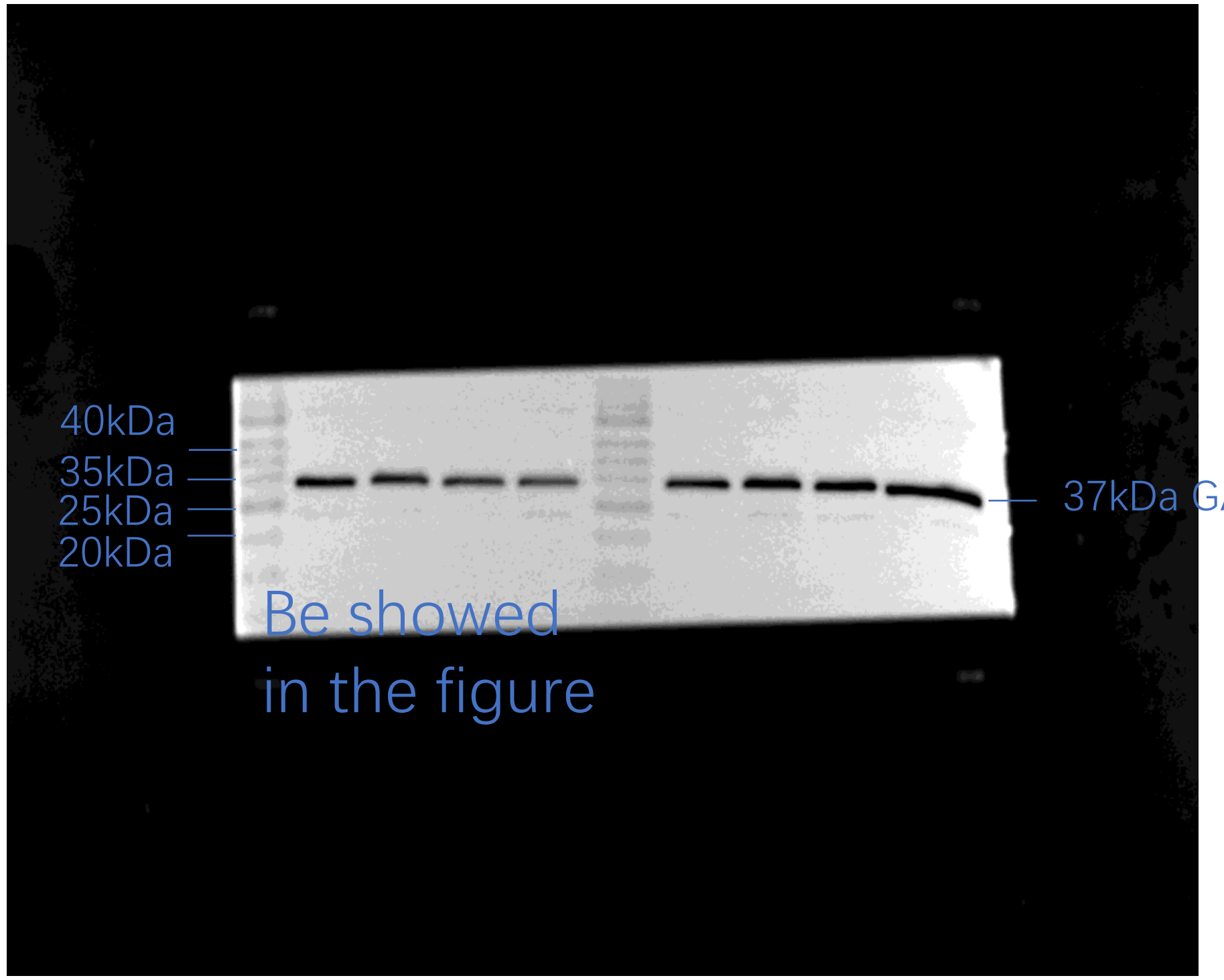

Figure7 A n = 3 independent replicates

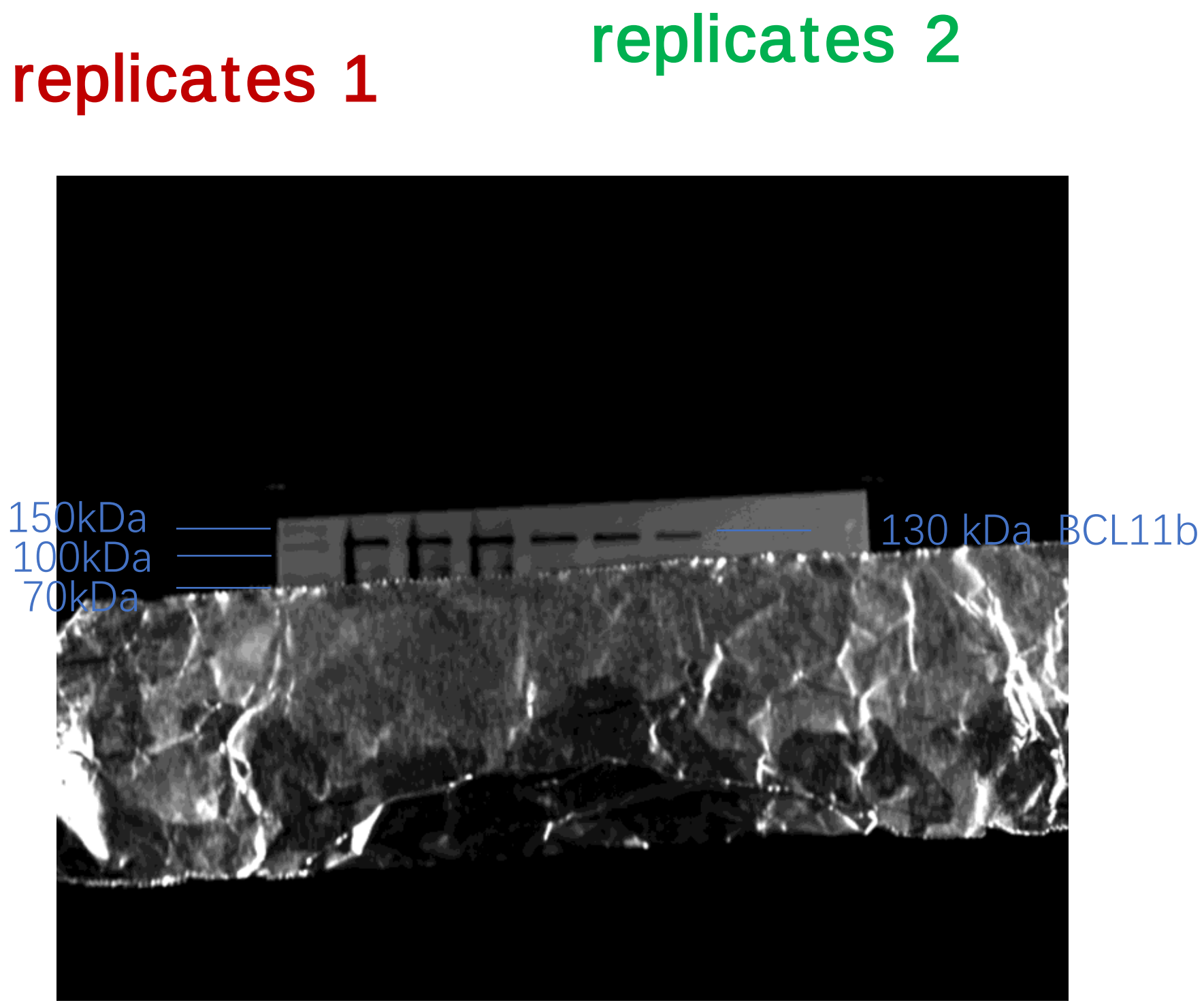

Be showed in the figure

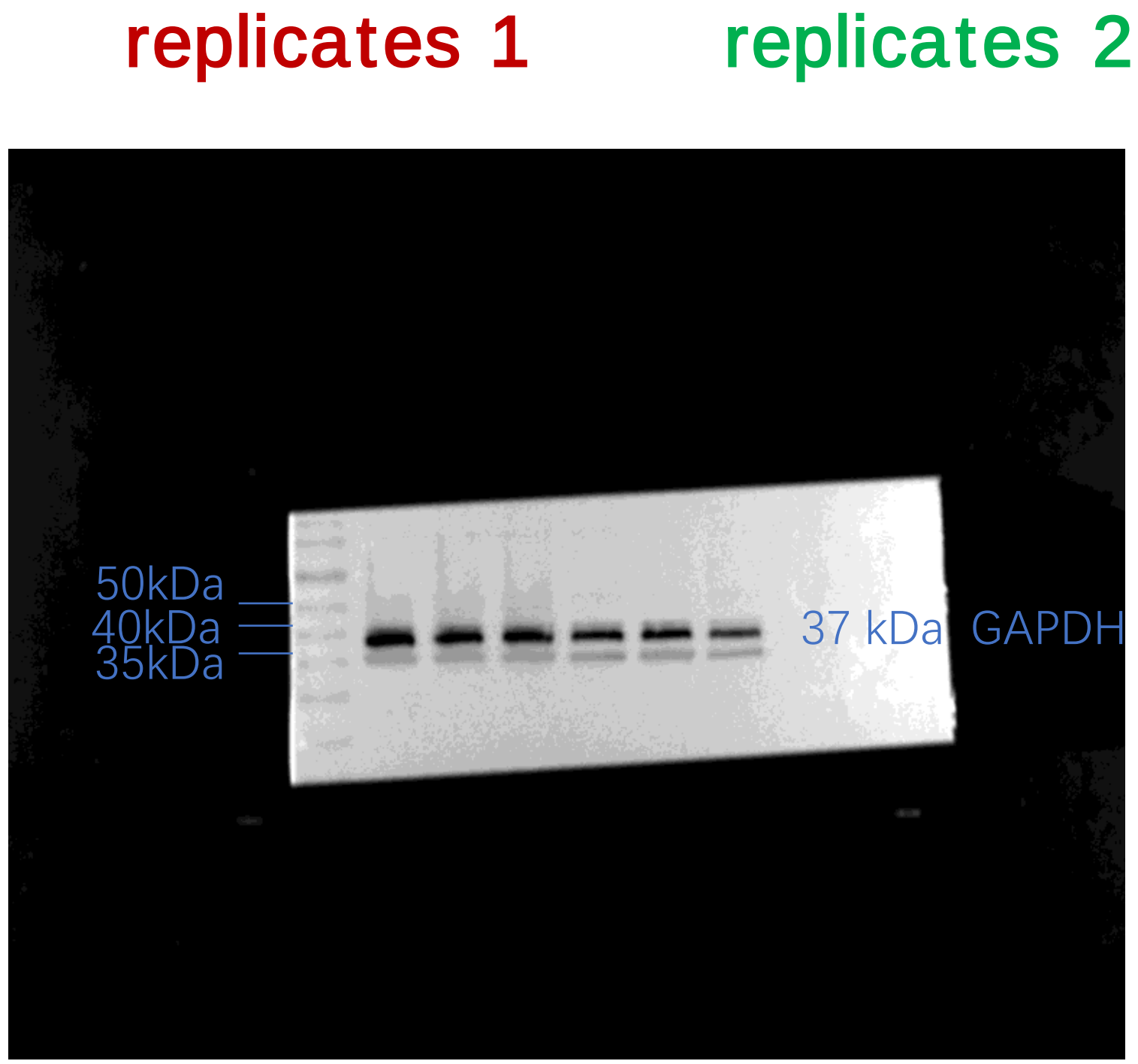

Be showed in the figure

Figure7 F n = 3 independent replicates

replicates 1

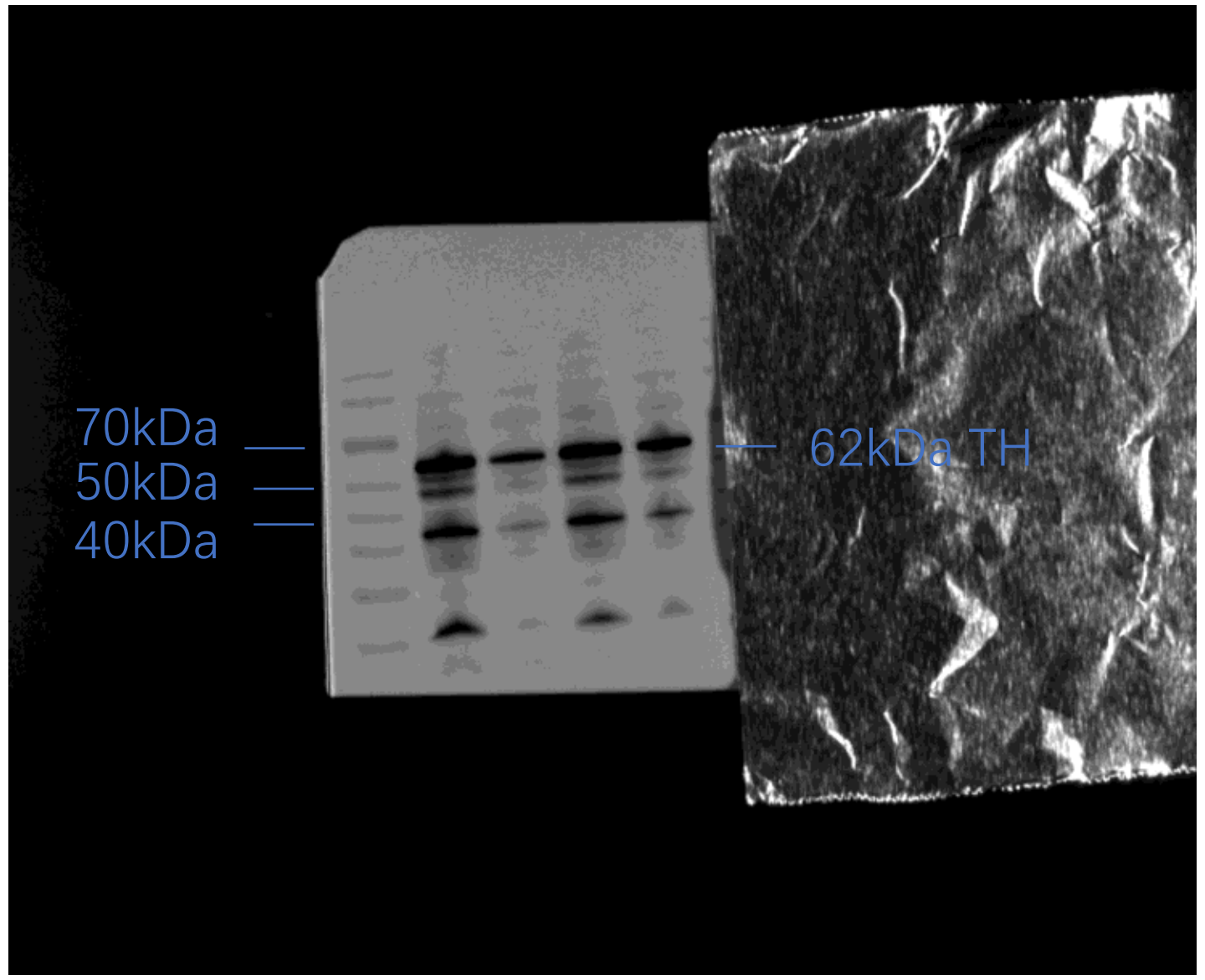

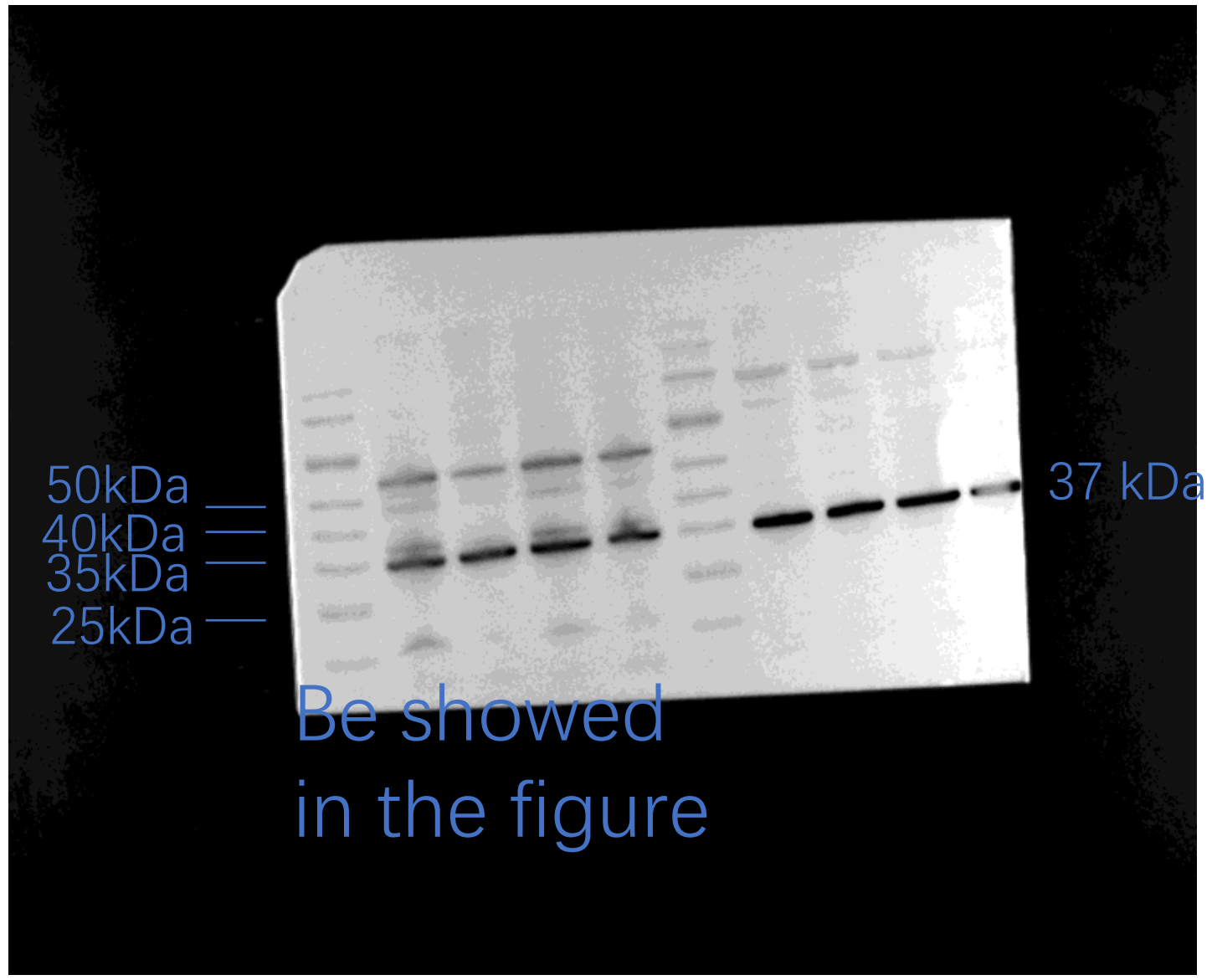

Be showed in the figure

replicates 2

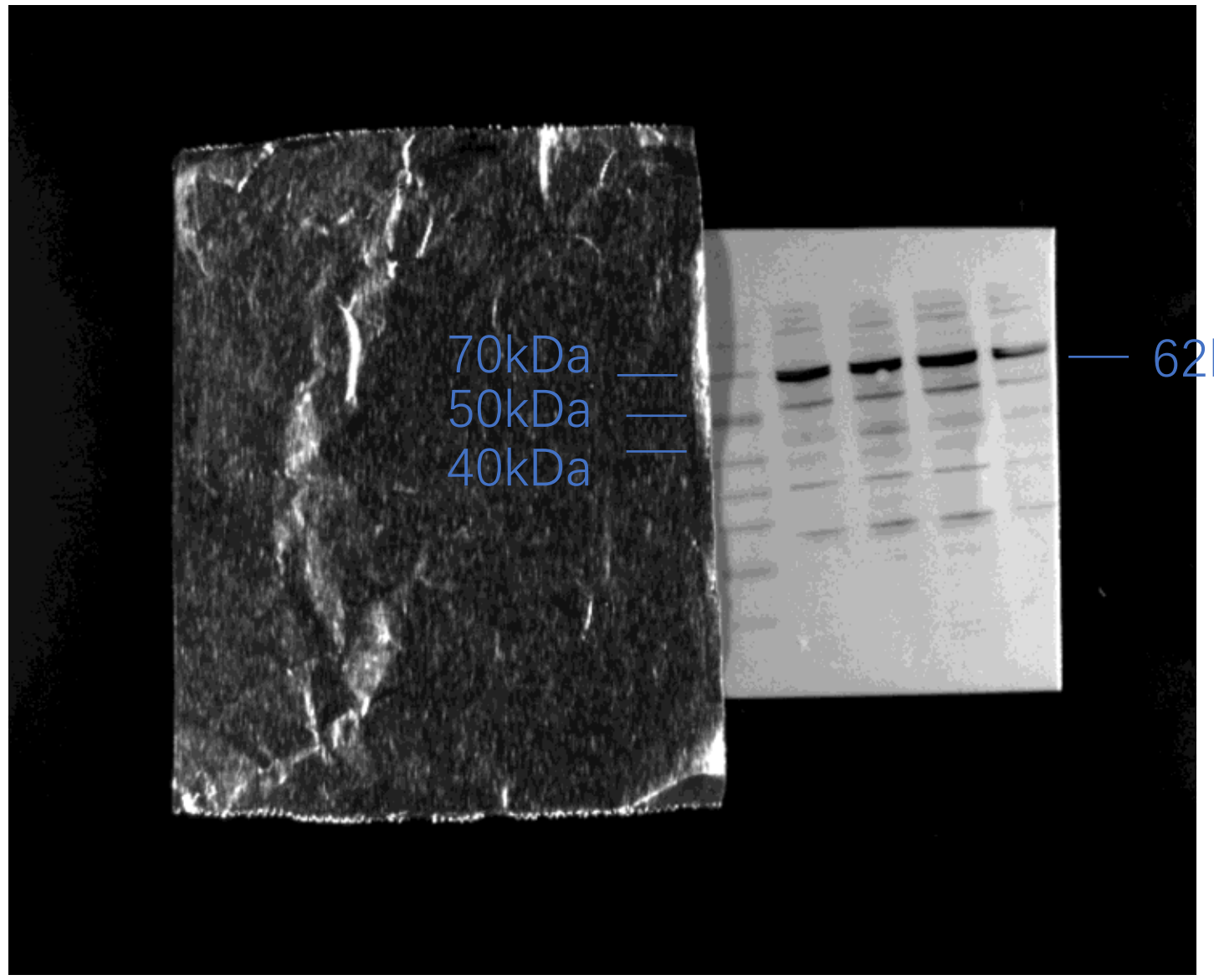

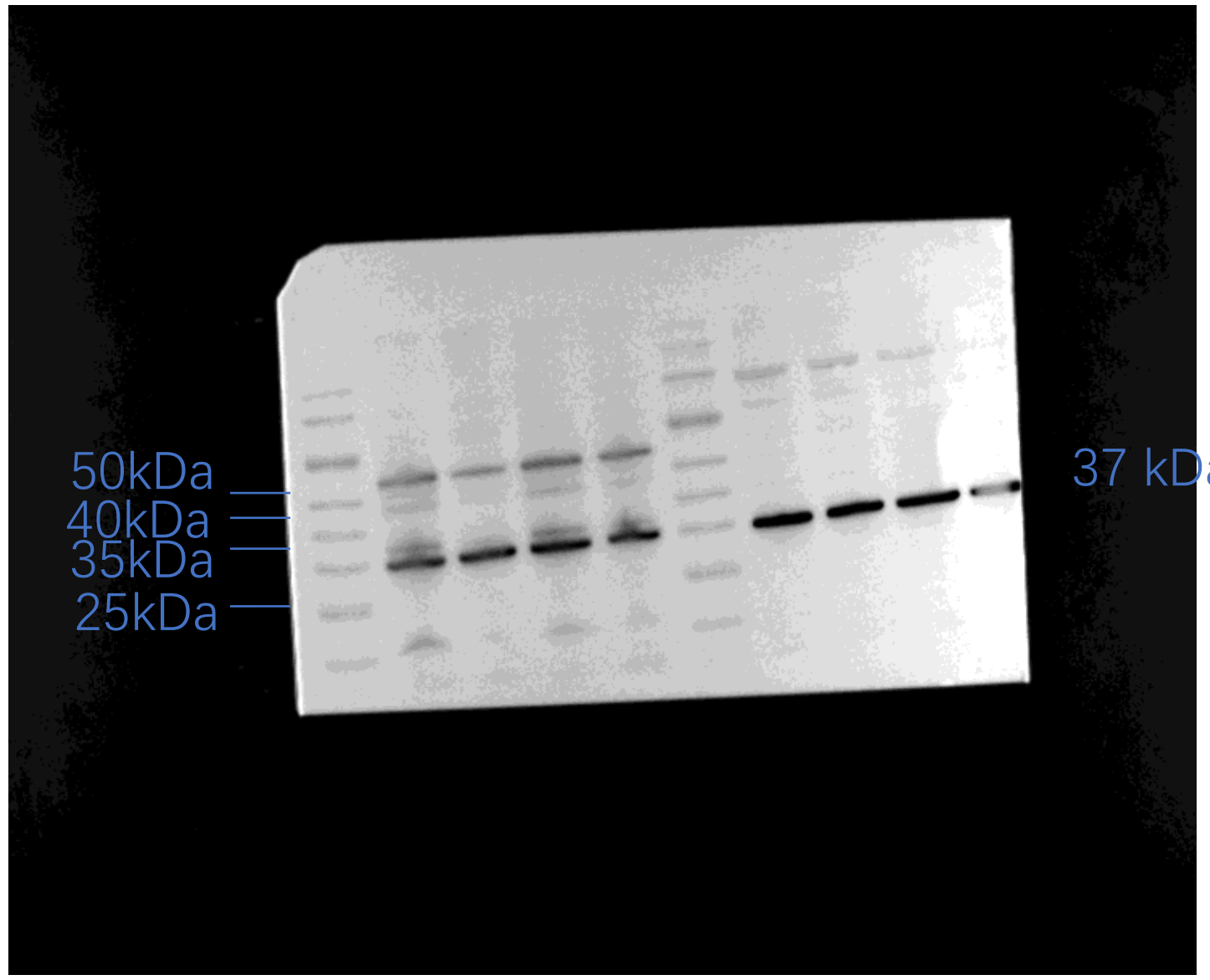

Figure7 F n = 3 independent replicates

replicates 1 replicates 2

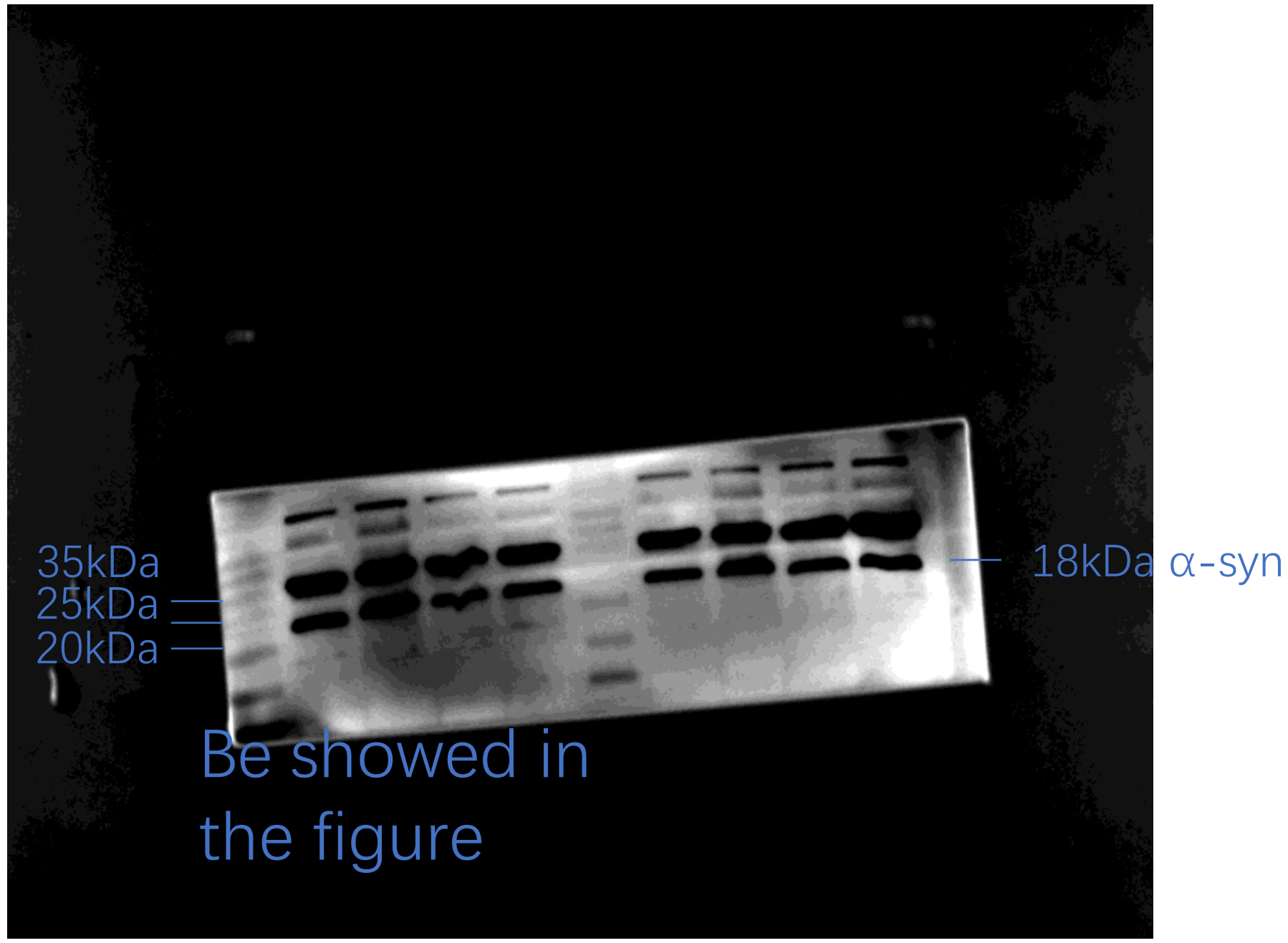

replicates 1 replicates 2

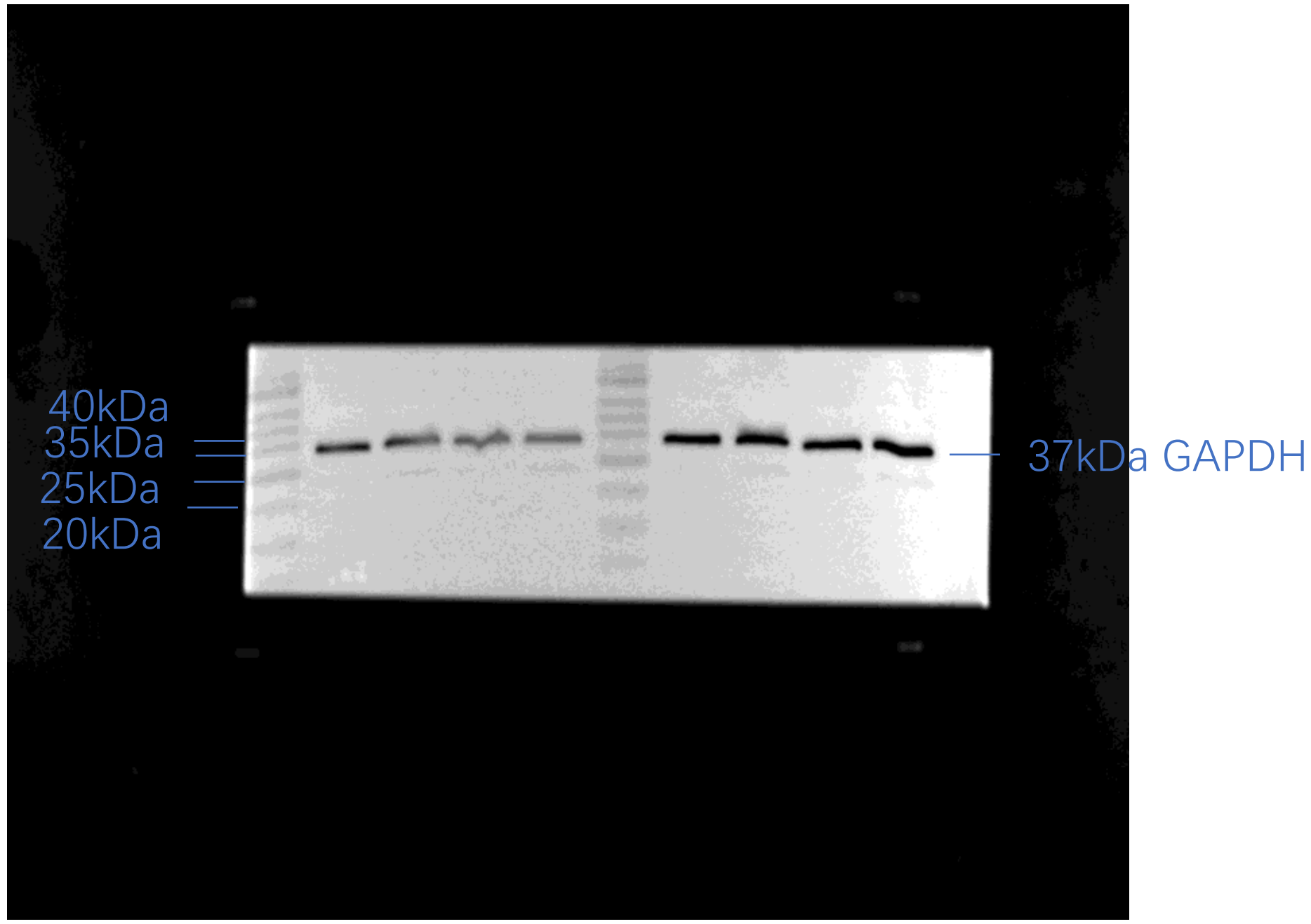

Figure8 C

n = 3 independent replicates

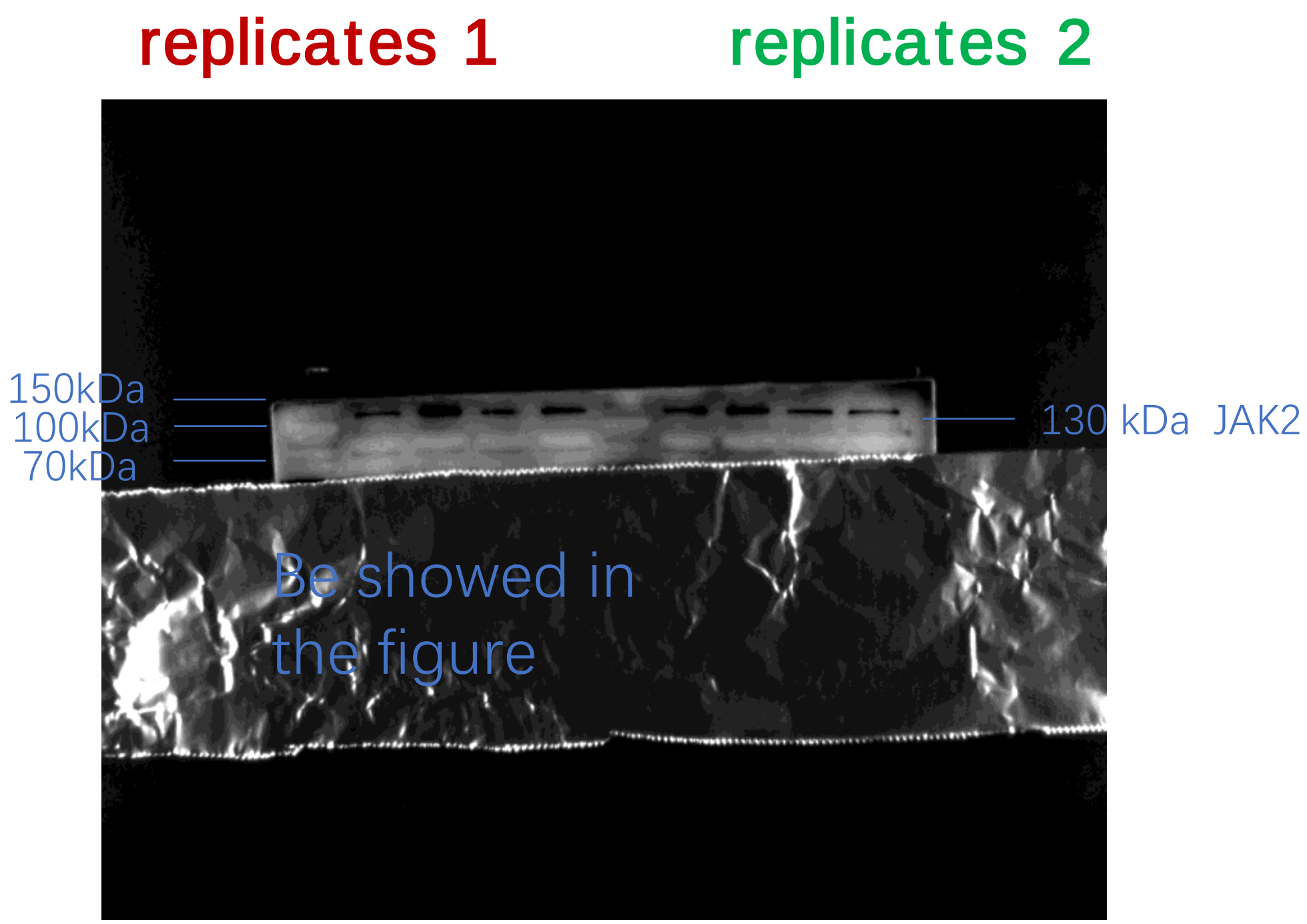

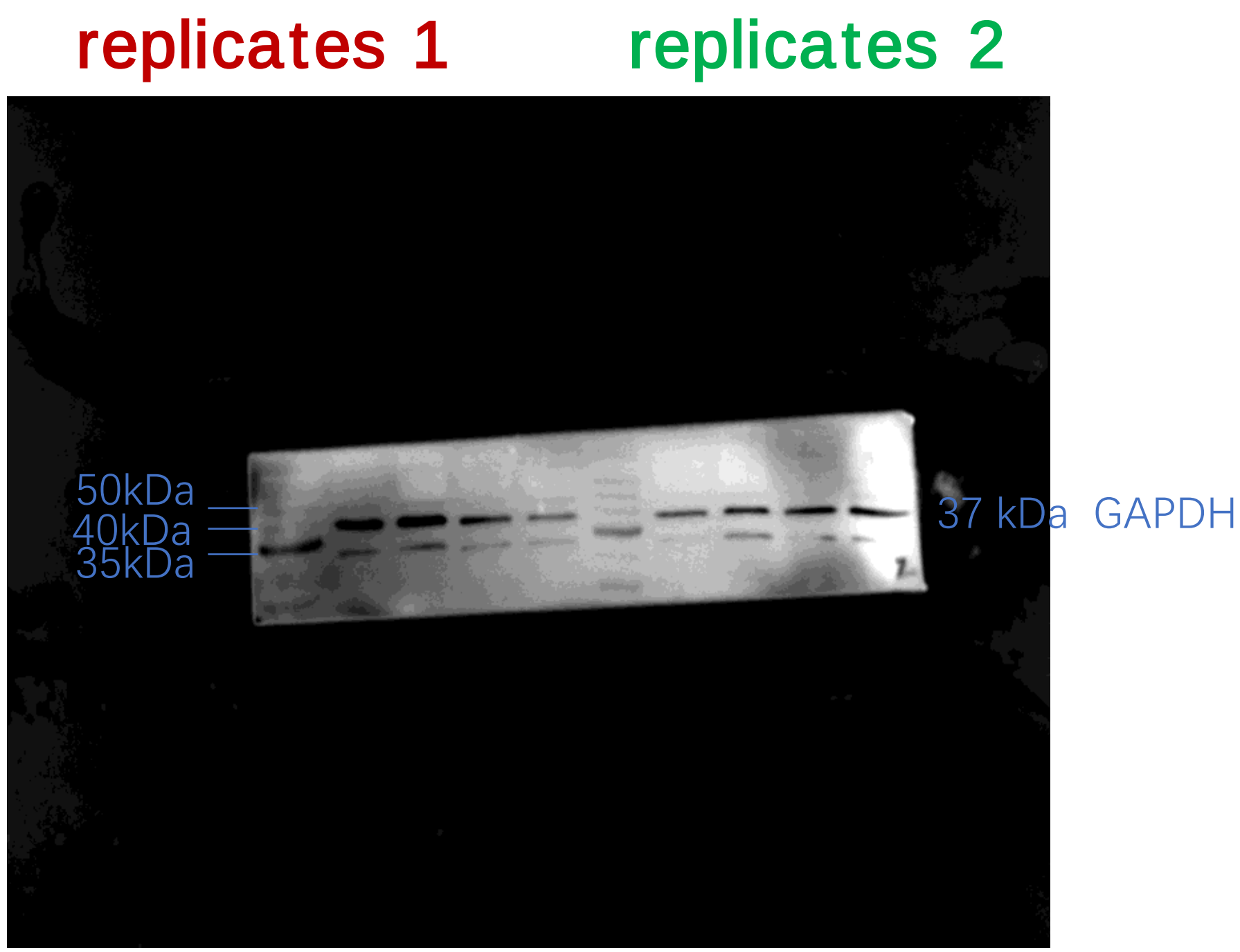

Figure8 C n = 3 independent replicates

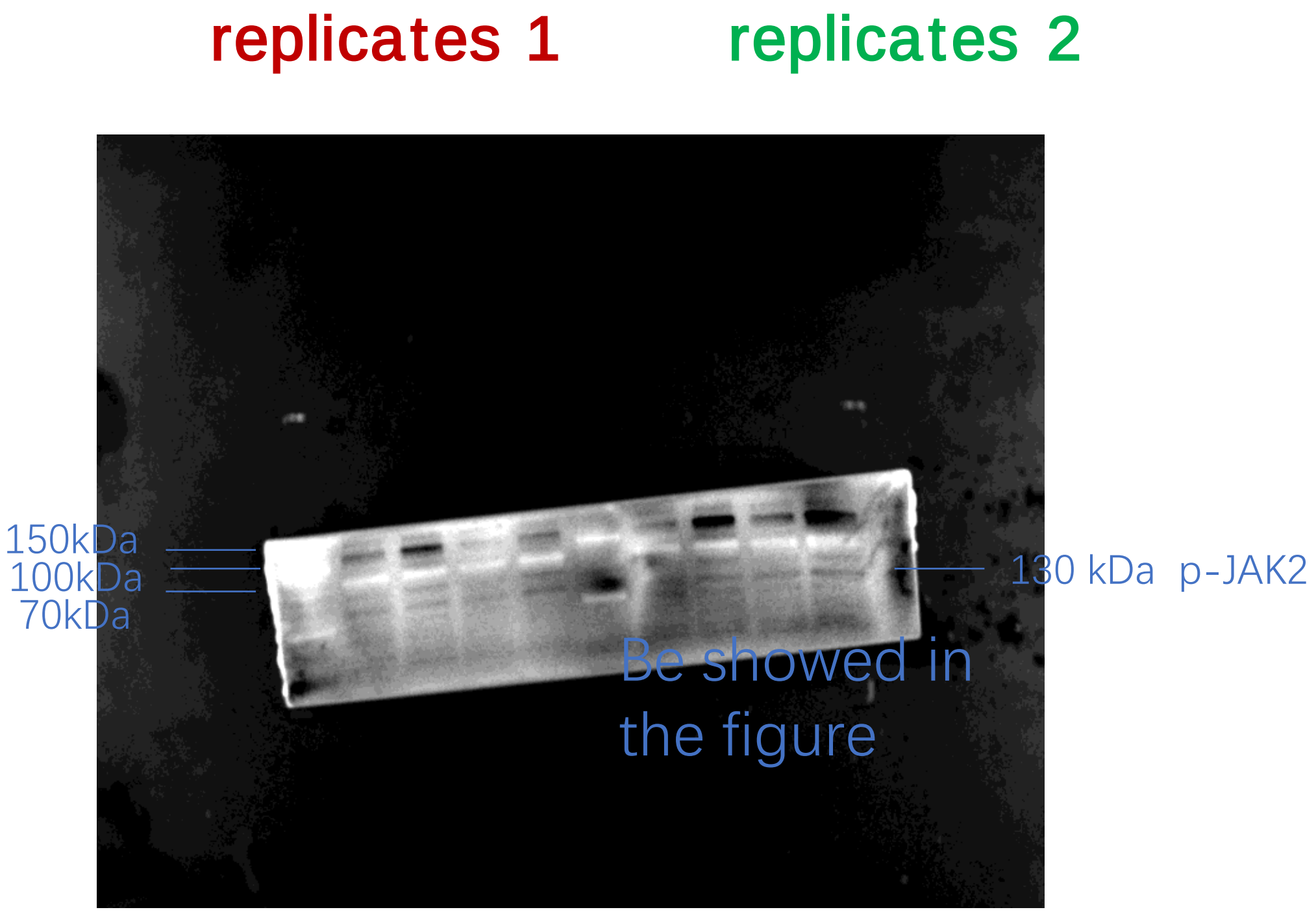

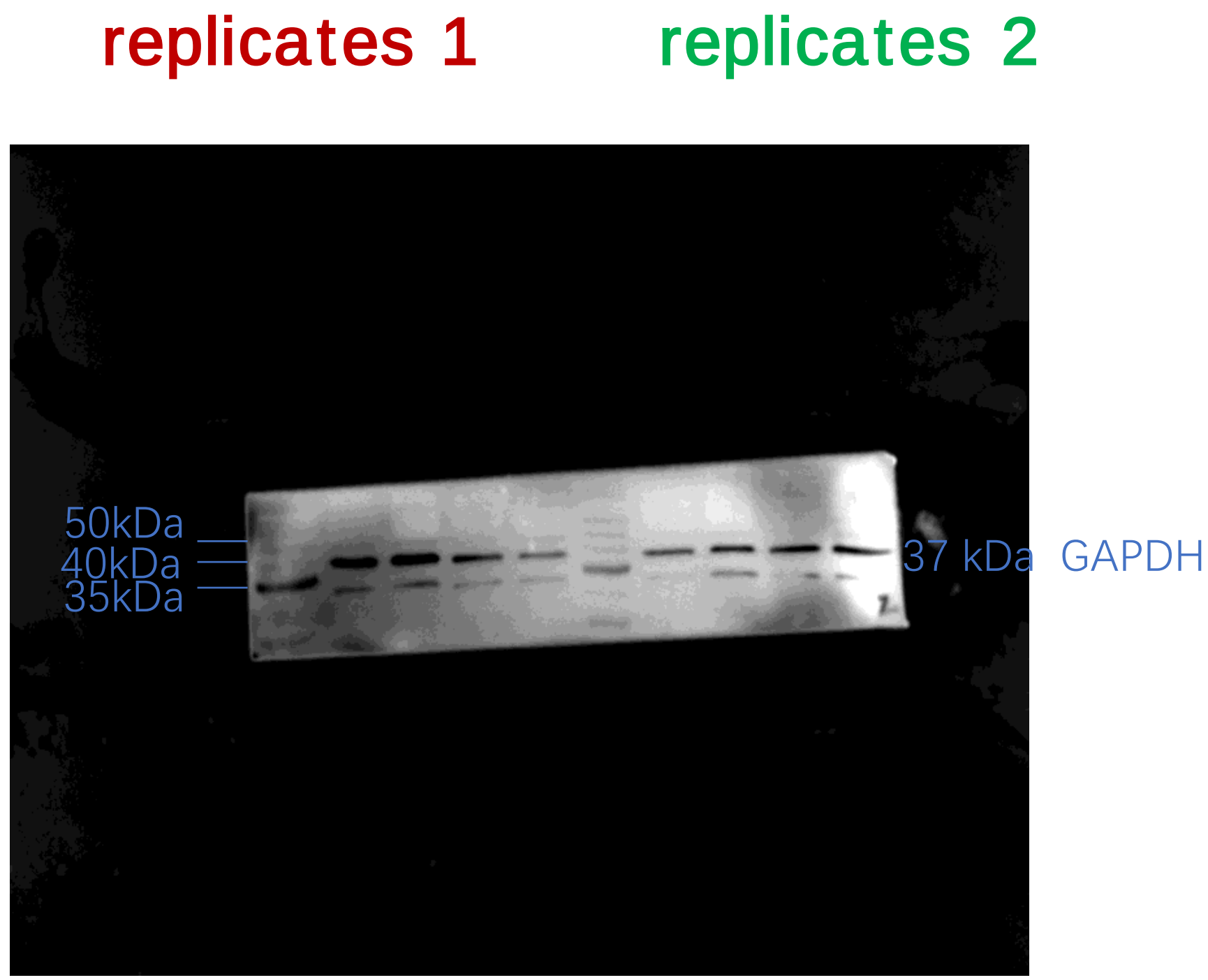

n = 3 independent replicates

Figure8 C

replicates 1

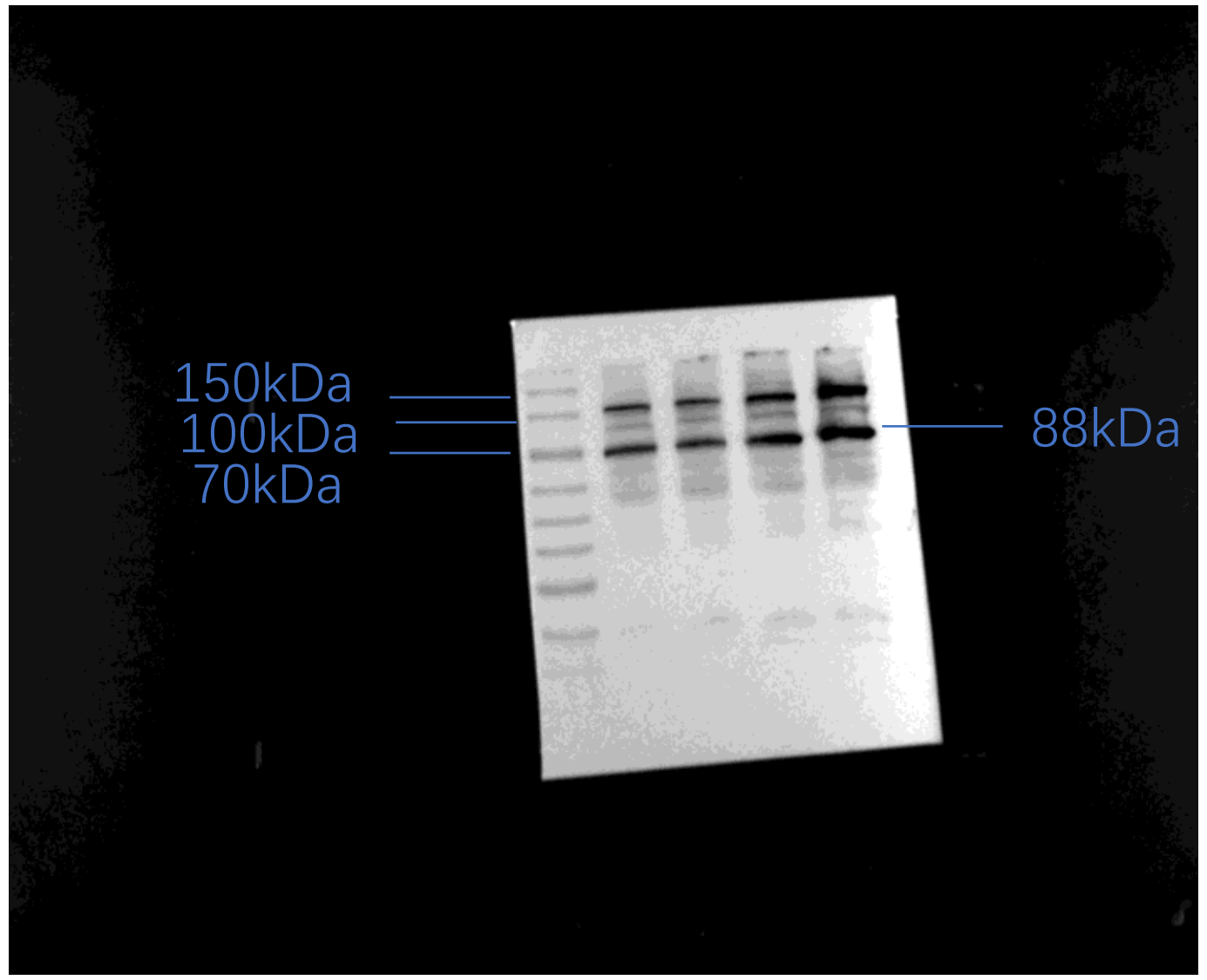

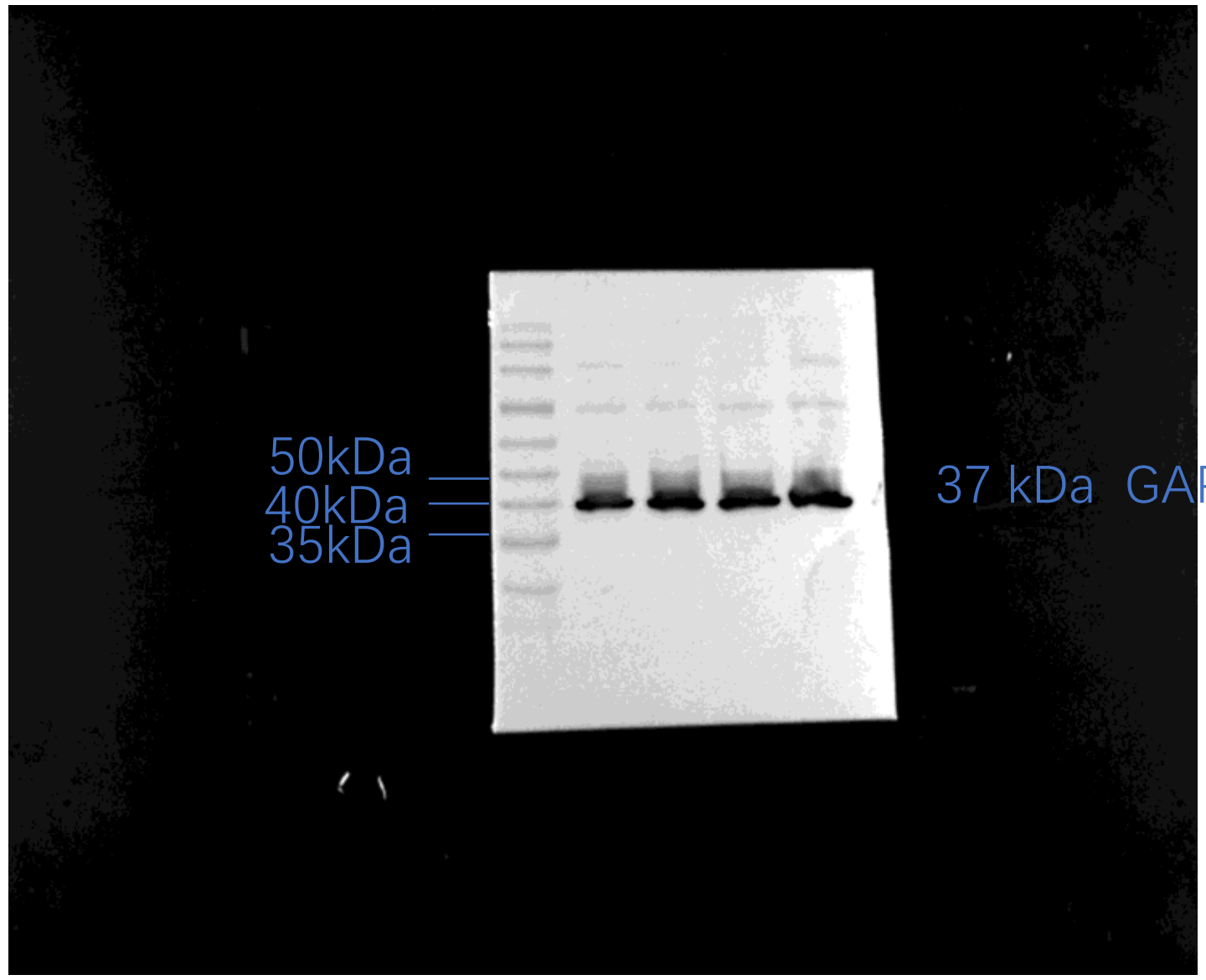

Be showed in the figure

Be showed in the figure

replicates 2

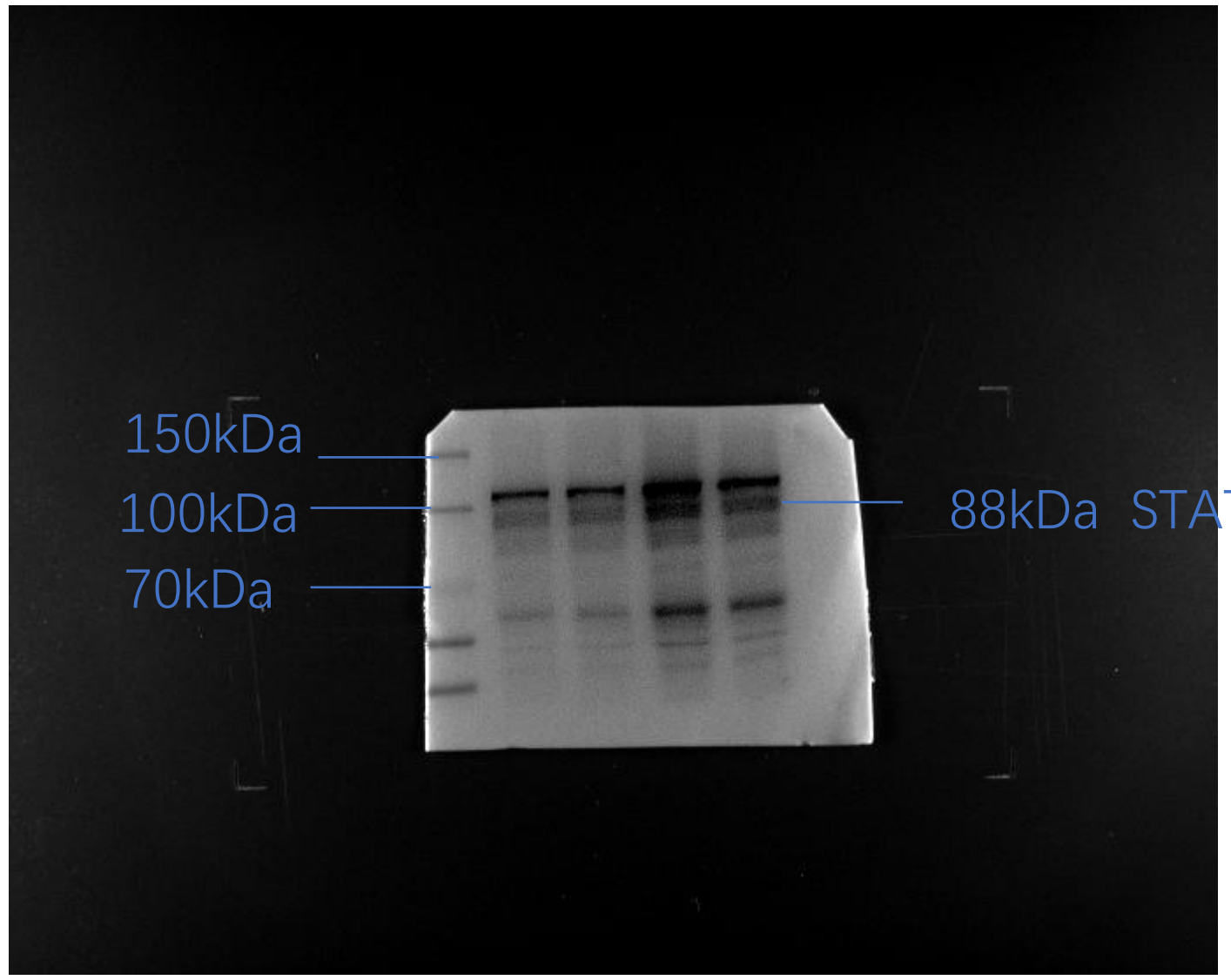

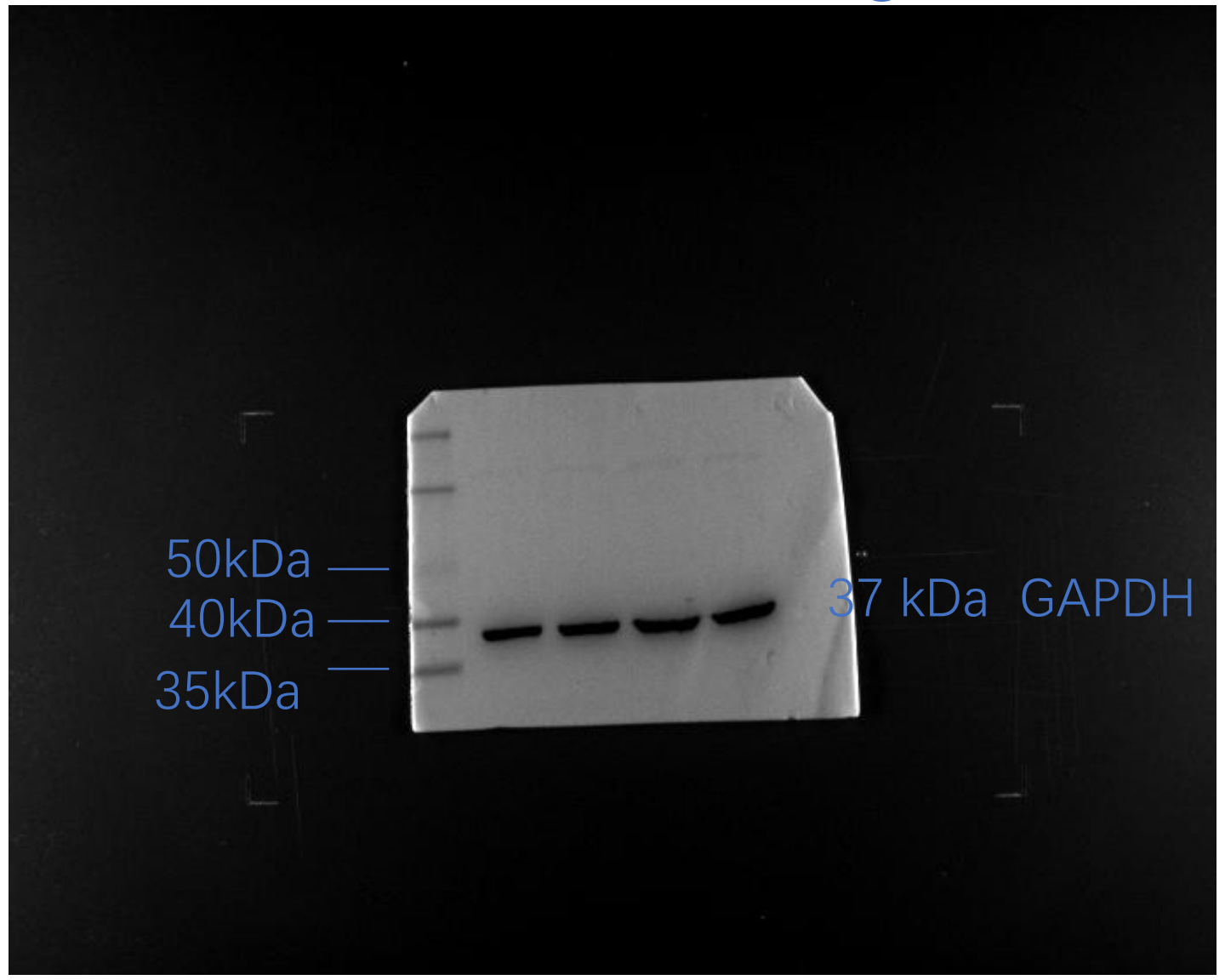

Figure8 C n = 3 independent replicates

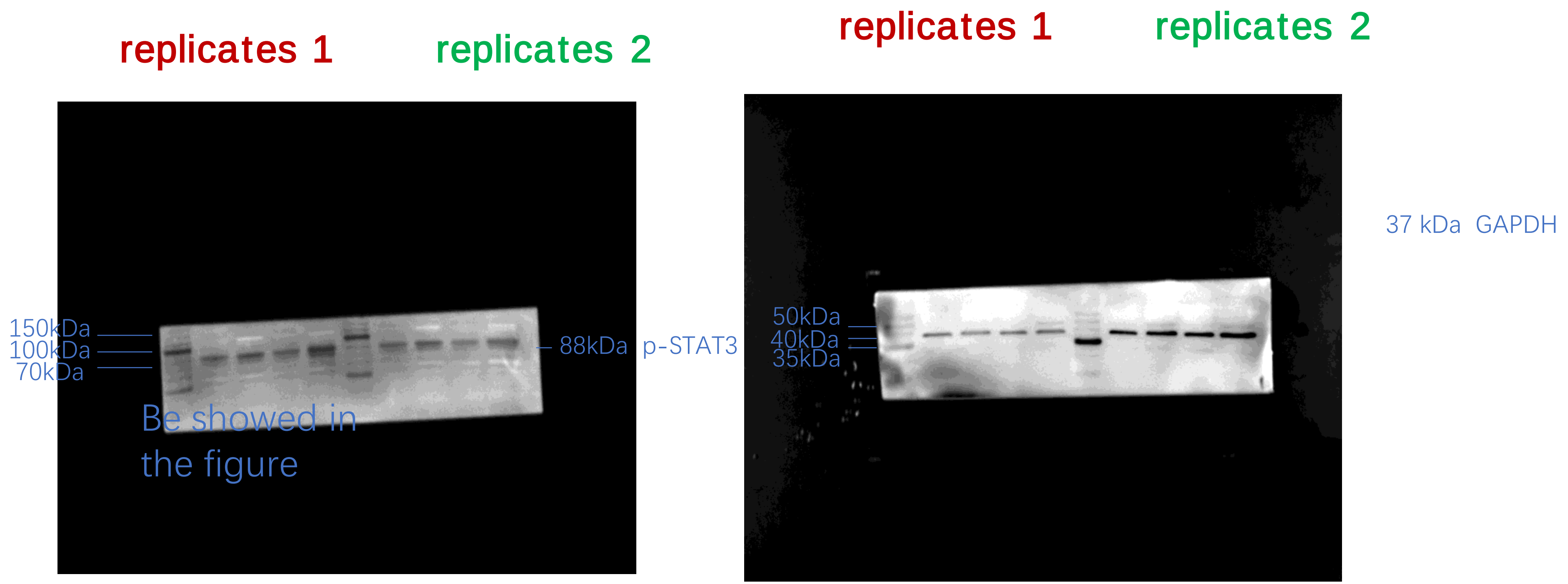

Figure8 F n = 3 independent replicates

replicates 1

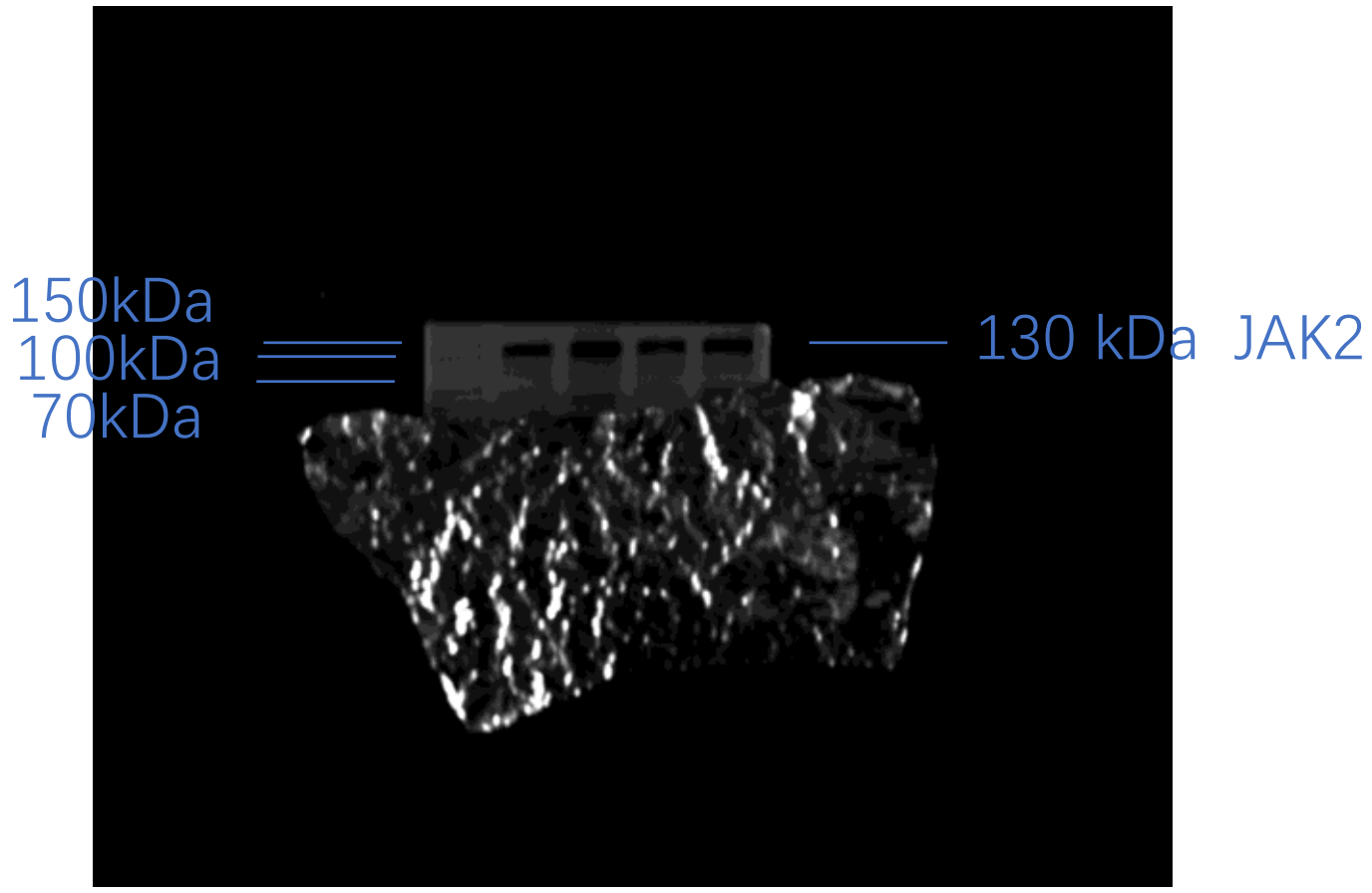

Be showed in the figure

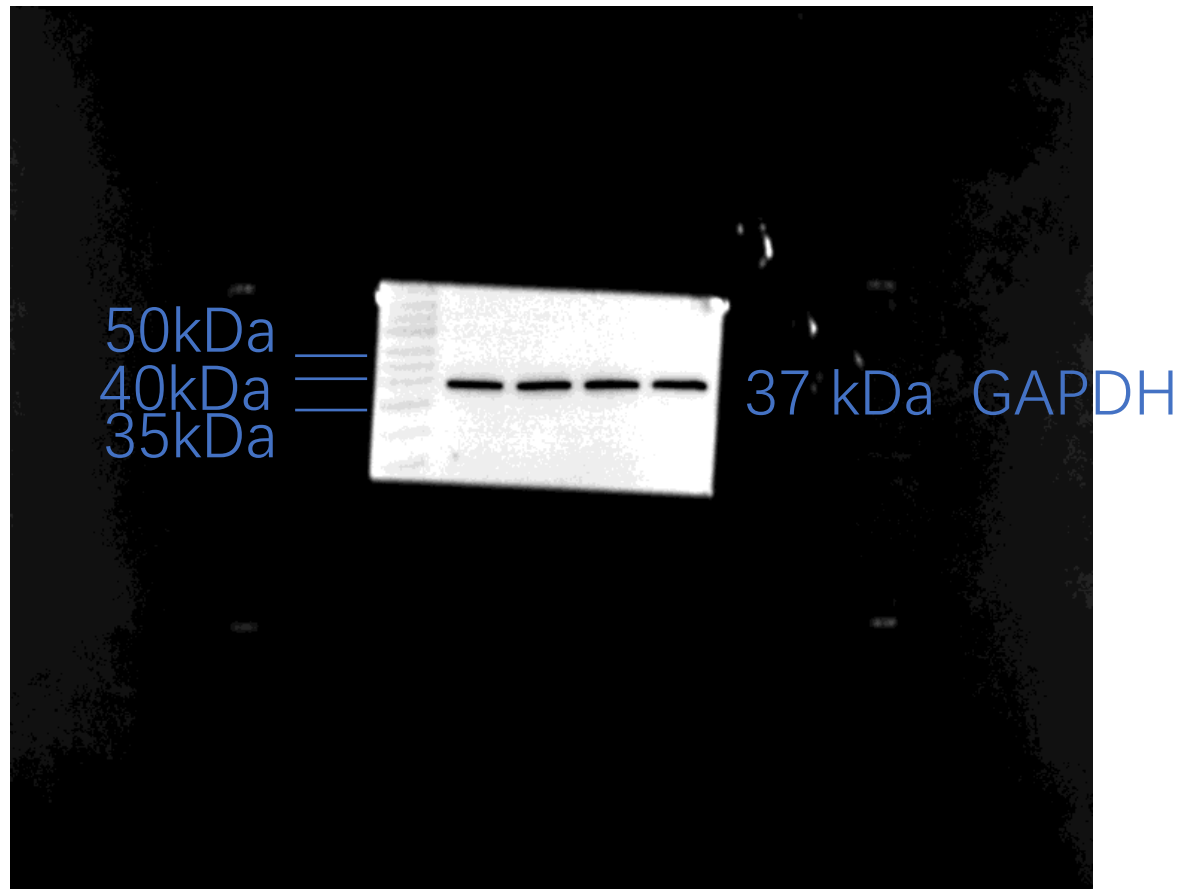

Be showed in the figure

replicates 2

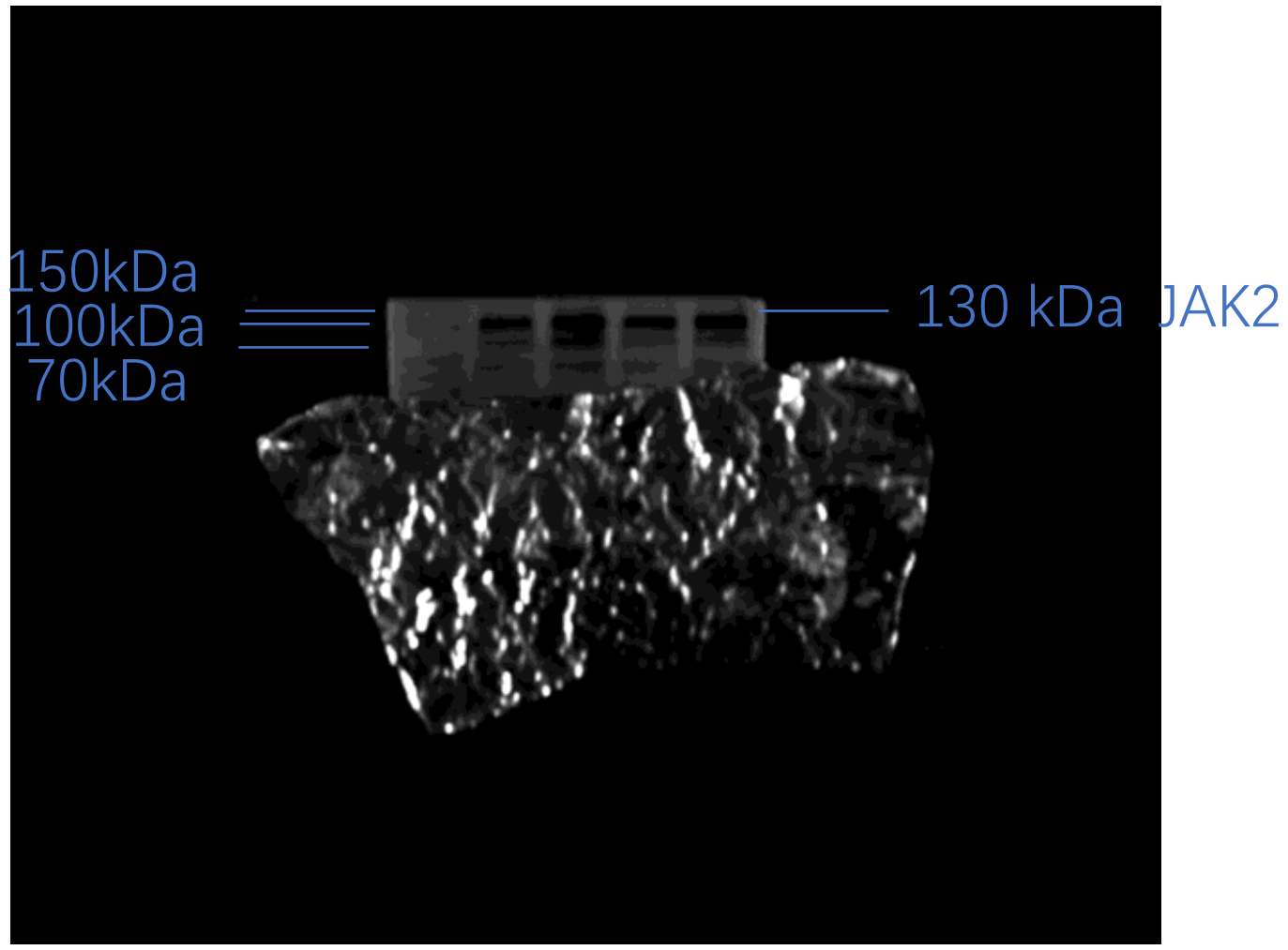

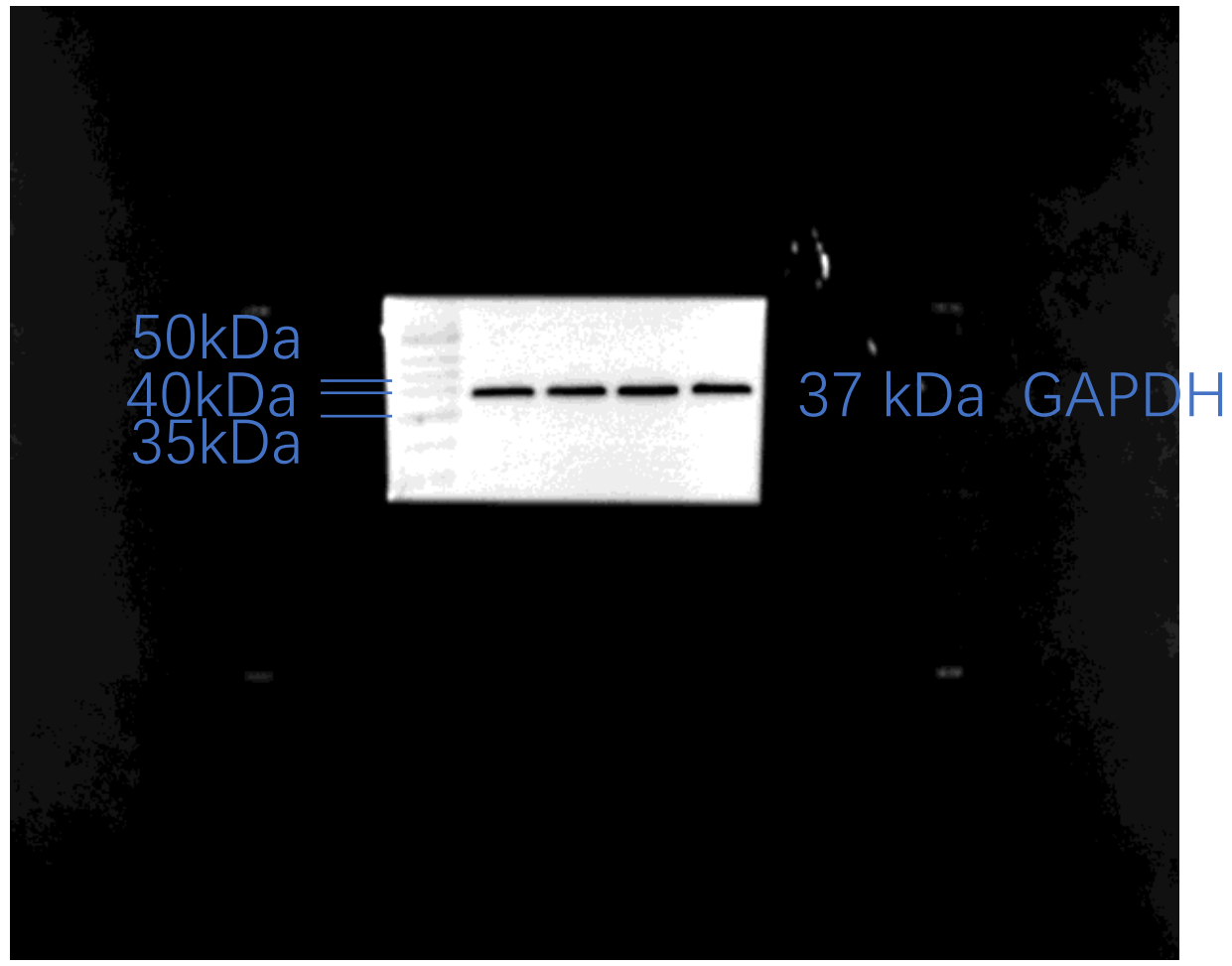

Figure8 F n = 3 independent replicates

replicates 1

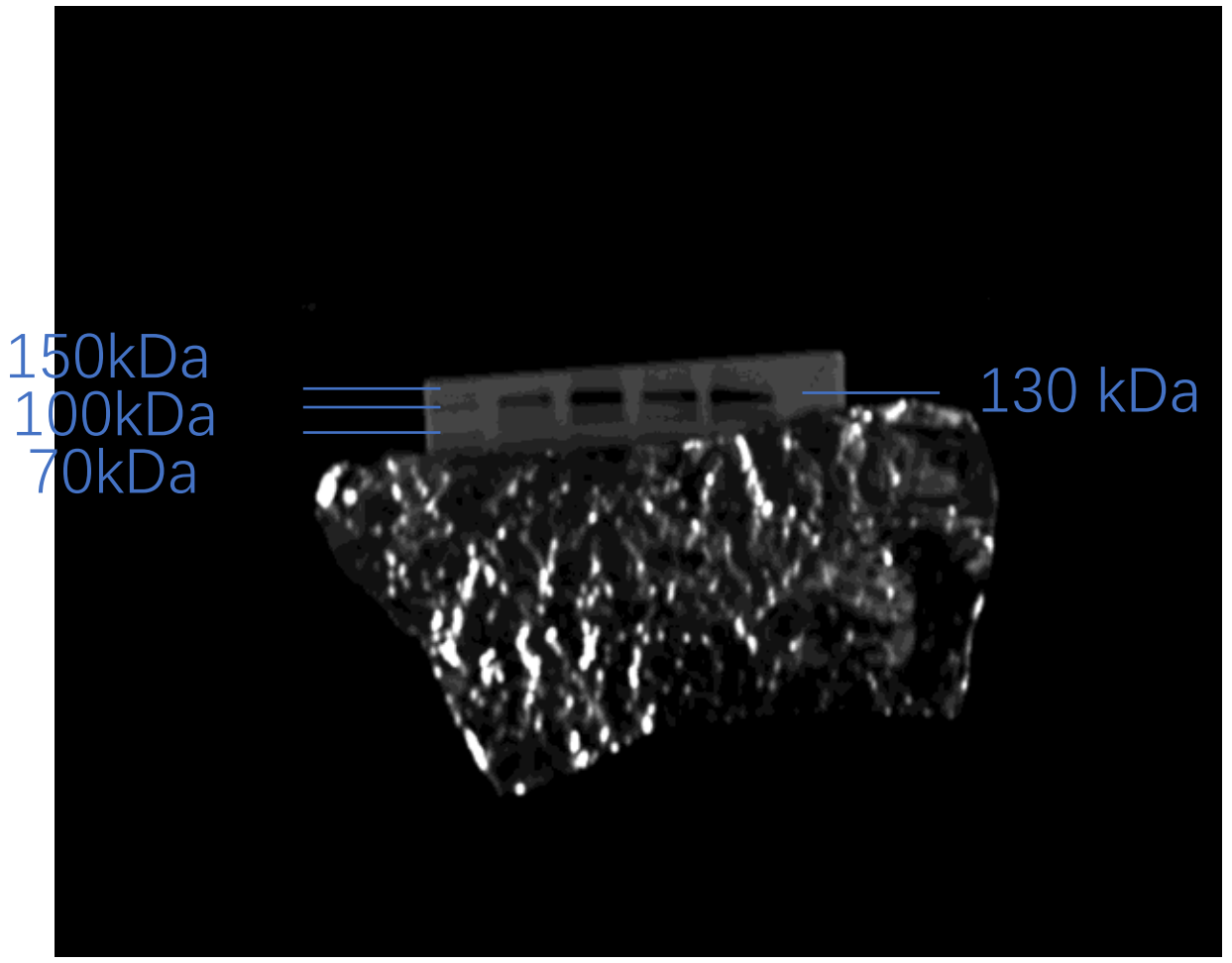

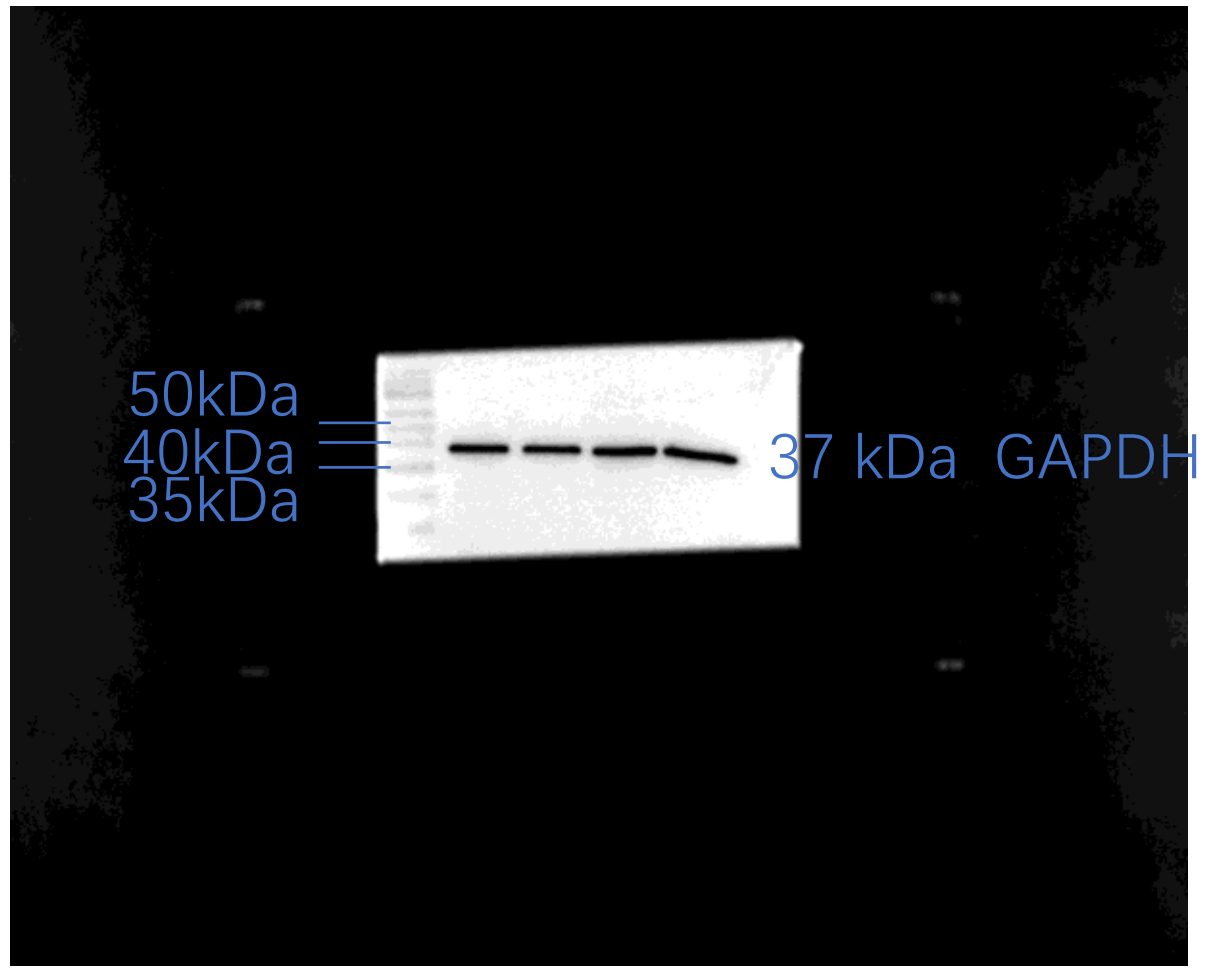

Be showed in the figure

replicates 2

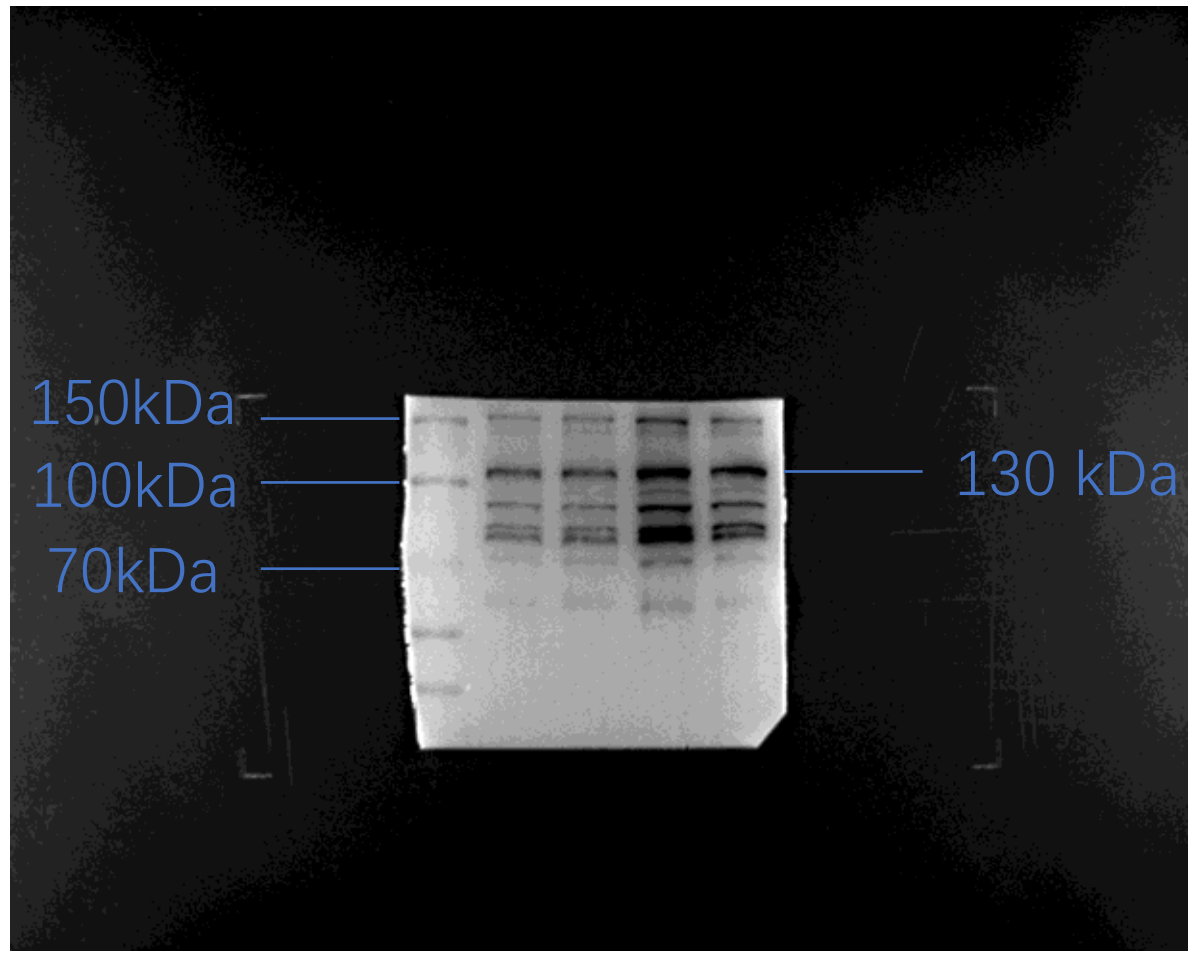

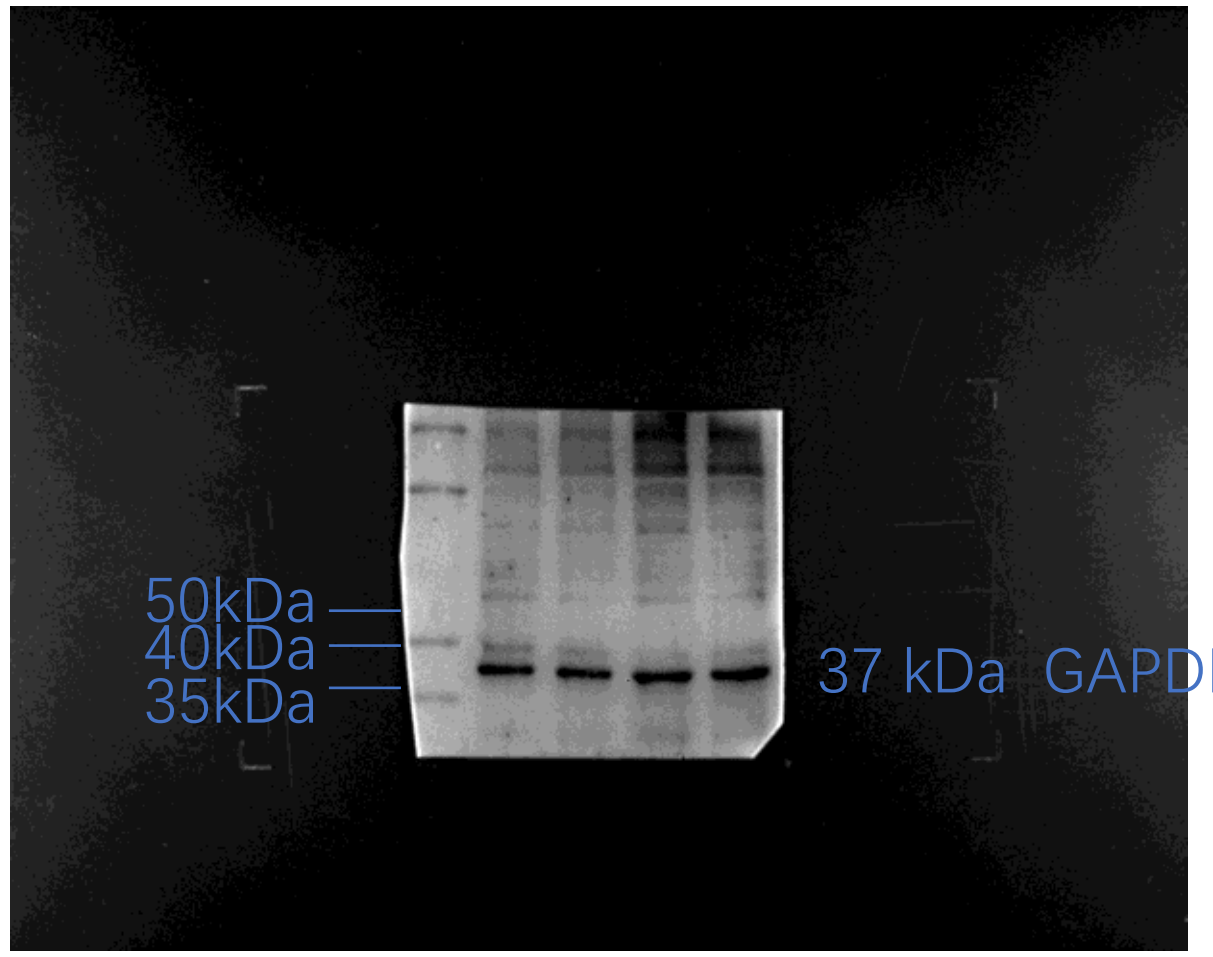

Figure8 F n = 3 independent replicates

replicates 1

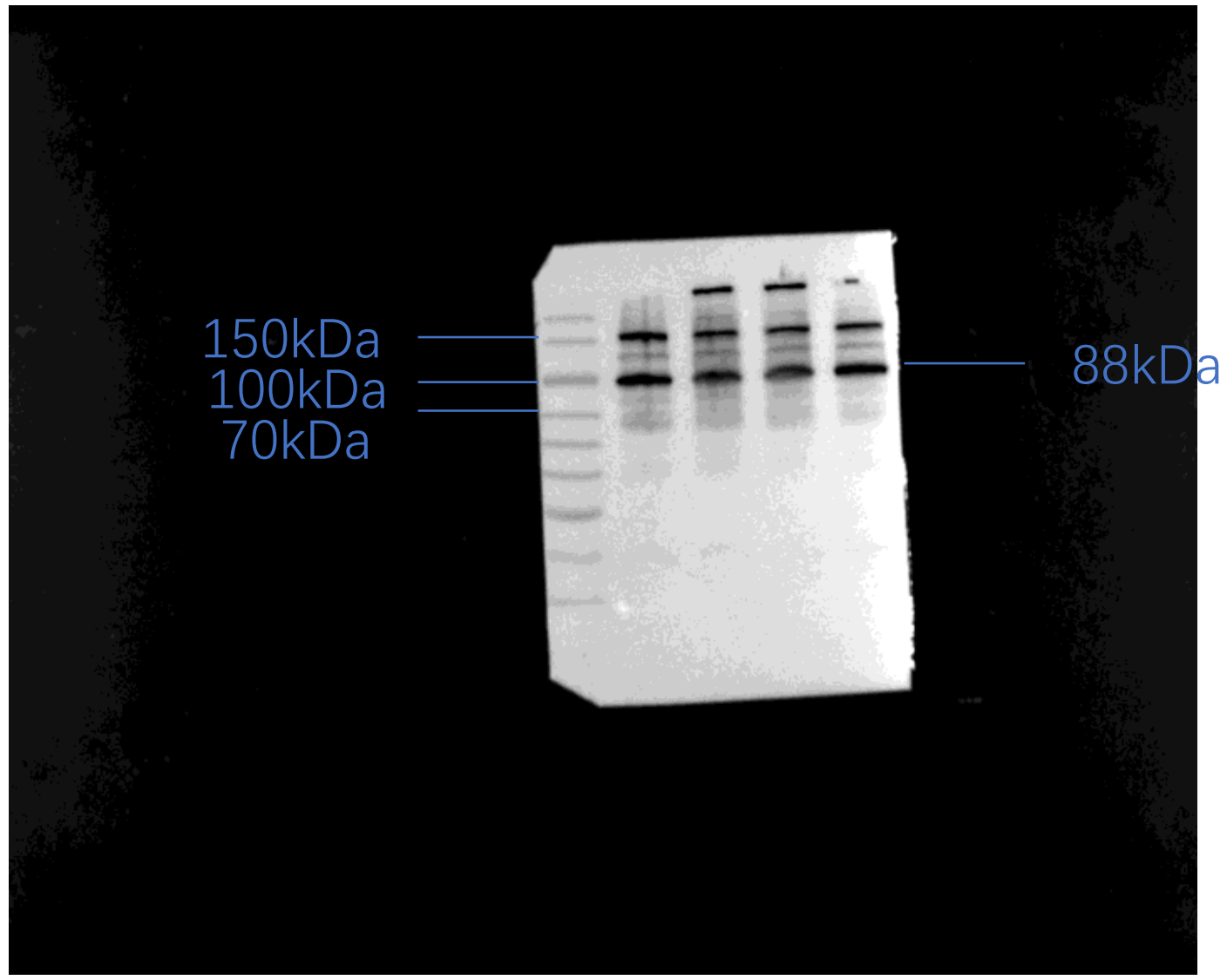

Be showed in the figure

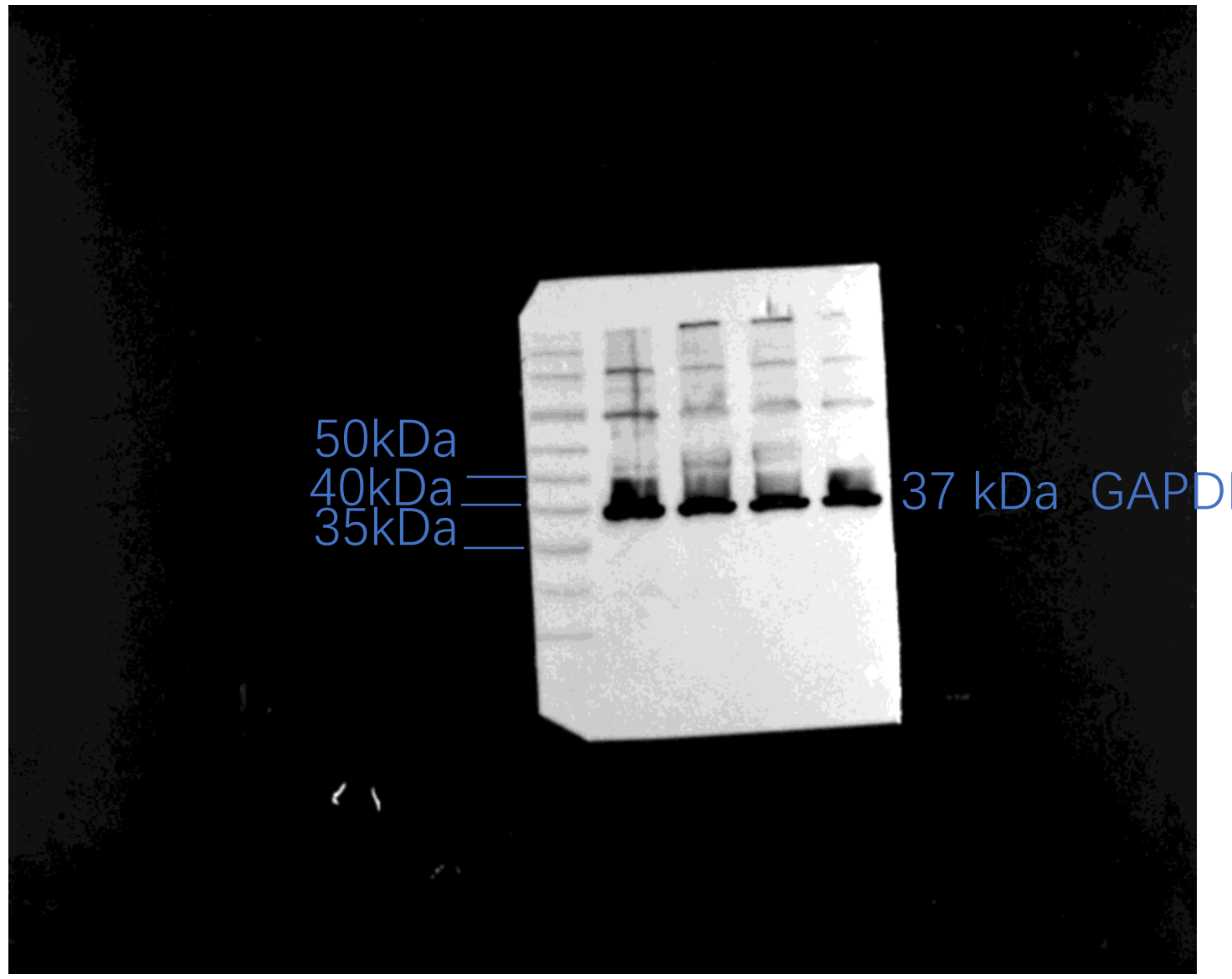

replicates 2

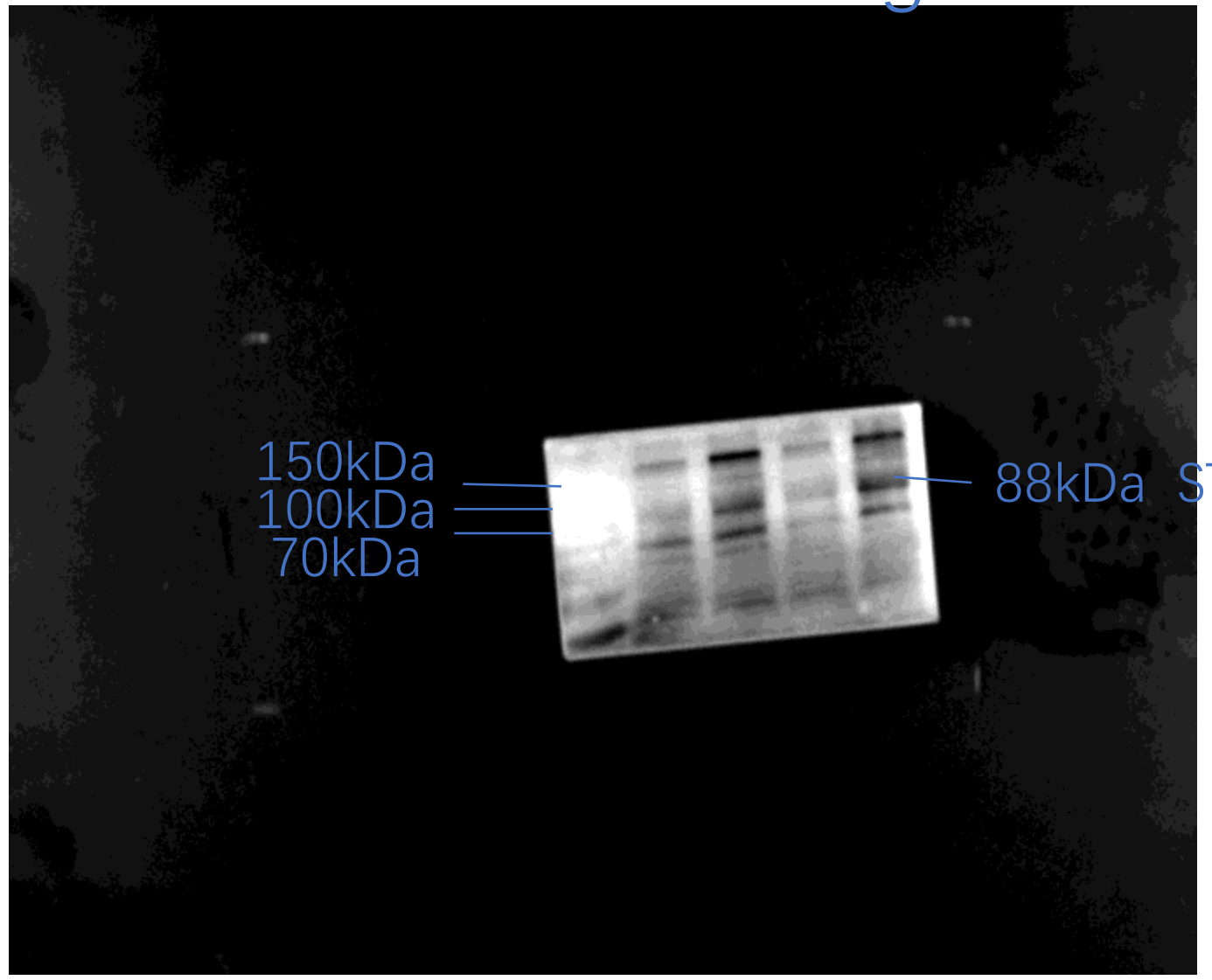

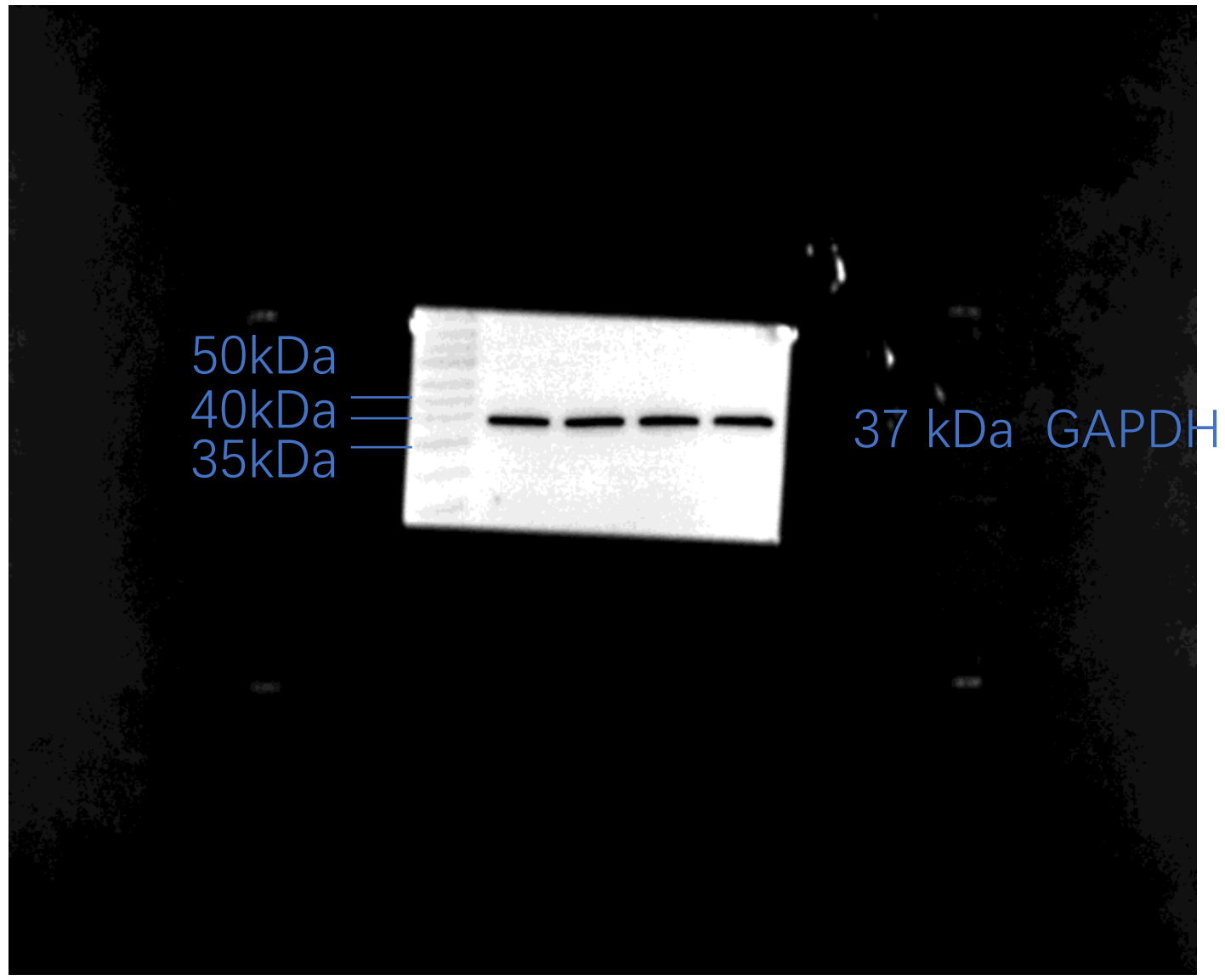

Figure8 F n = 3 independent replicates

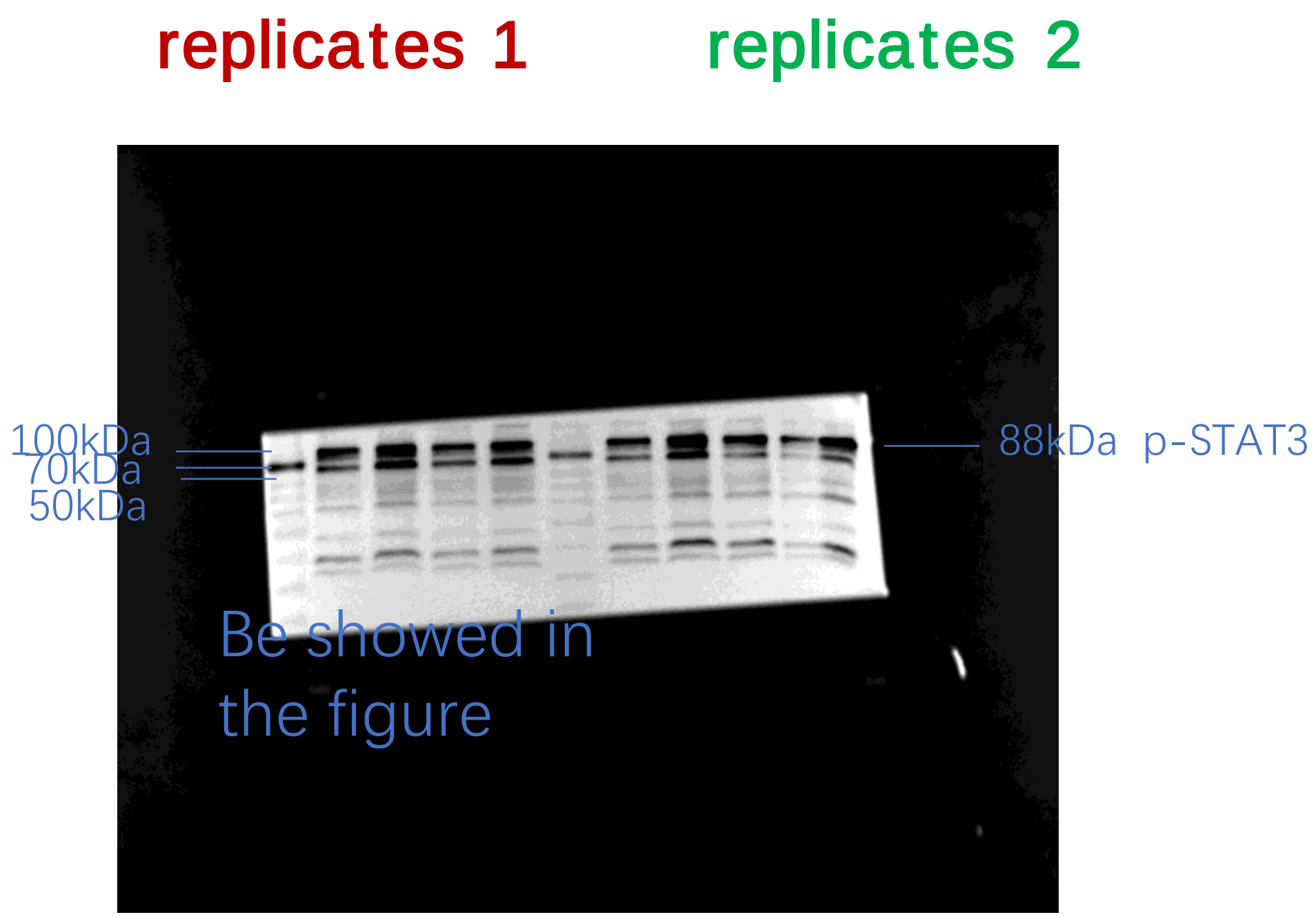

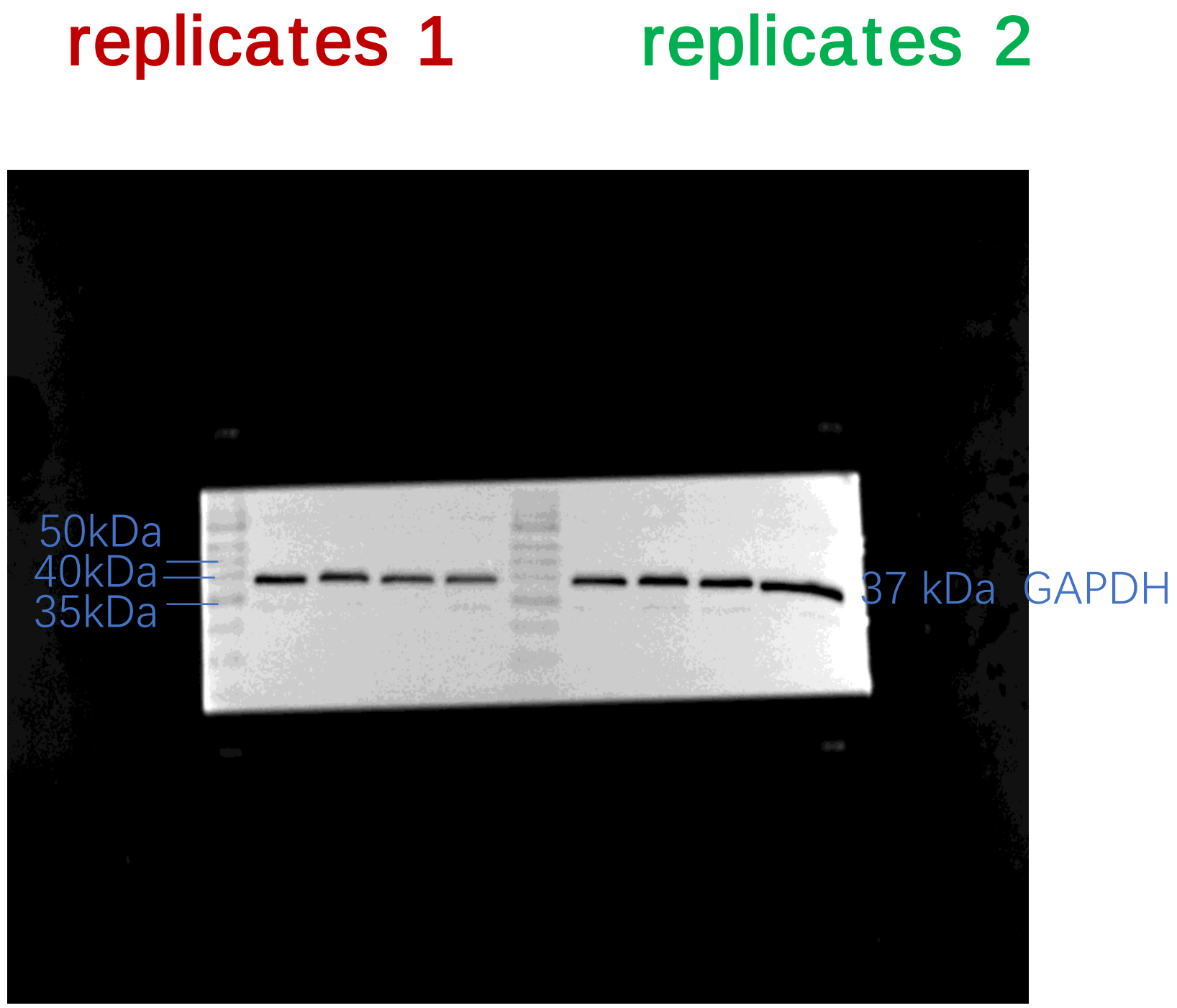

Supplement: Supplementary file 2 [file mmc2.pdf]
